# Multiomics analysis of umbilical cord hematopoietic stem cells from a multiethnic cohort of Hawaii reveals the intergenerational effect of maternal prepregnancy obesity and risks for cancers

**DOI:** 10.1093/gigascience/giaf039

**Published:** 2025-05-19

**Authors:** Yuheng Du, Paula A Benny, Yuchen Shao, Ryan J Schlueter, Alexandra Gurary, Annette Lum-Jones, Cameron B Lassiter, Fadhl M AlAkwaa, Maarit Tiirikainen, Dena Towner, W Steven Ward, Lana X Garmire

**Affiliations:** Department of Computational Medicine and Bioinformatics, University of Michigan, Ann Arbor, MI 48109, USA; Department of Obstetrics and Gynecology, University of Hawaii, Honolulu, HI 96826, USA; Department of Electrical Engineering and Computer Science, University of Michigan, Ann Arbor, MI 48109, USA; Department of Obstetrics and Gynecology, University of Hawaii, Honolulu, HI 96826, USA; Department of Obstetrics and Gynecology, University of Hawaii, Honolulu, HI 96826, USA; University of Hawaii Cancer Center, Population Sciences of the Pacific Program-Epidemiology, Honolulu, HI 96813, USA; University of Hawaii Cancer Center, Population Sciences of the Pacific Program-Epidemiology, Honolulu, HI 96813, USA; Department of Neurology, University of Michigan, Ann Arbor, MI 48109, USA; University of Hawaii Cancer Center, Population Sciences of the Pacific Program-Epidemiology, Honolulu, HI 96813, USA; Department of Obstetrics and Gynecology, University of Hawaii, Honolulu, HI 96826, USA; Department of Obstetrics and Gynecology, University of Hawaii, Honolulu, HI 96826, USA; Department of Computational Medicine and Bioinformatics, University of Michigan, Ann Arbor, MI 48109, USA

**Keywords:** obesity, Native Hawaiian, hematopoietic stem cells, multiomics, cord blood, methylation, pregnancy

## Abstract

**Background:**

Maternal obesity is a health concern that may predispose newborns to a high risk of medical problems later in life. To understand the intergenerational effect of maternal obesity, we hypothesized that the maternal obesity effect is mediated by epigenetic changes in the CD34+/CD38−/Lin− hematopoietic stem cells (uHSCs) in the offspring. To investigate this, we conducted a DNA methylation centric multiomics study. We measured DNA methylation and gene expression of the CD34+/CD38−/Lin− uHSCs and metabolomics of the cord blood, all from a multiethnic cohort from Kapiolani Medical Center for Women and Children in Honolulu, Hawaii (n=72, collected between 2016 and 2018).

**Results:**

Differential methylation analysis unveiled a global hypermethylation pattern in the maternal prepregnancy obese group (BH adjusted *P* < 0.05), after adjusting for major clinical confounders. KEGG pathway enrichment, WGCNA, and PPI analyses revealed that hypermethylated CpG sites were involved in critical biological processes, including cell cycle, protein synthesis, immune signaling, and lipid metabolism. Utilizing Shannon entropy on uHSCs methylation, we discerned notably higher quiescence of uHSCs impacted by maternal obesity. Additionally, the integration of multiomics data—including methylation, gene expression, and metabolomics—provided further evidence of dysfunctions in adipogenesis, erythropoietin production, cell differentiation, and DNA repair, aligning with the findings at the epigenetic level. Furthermore, we trained a random forest classifier using the CpG sites in the genes of the top pathways associated with maternal obesity, and applied it to predict cancer versus adjacent normal sample labels in 14 Cancer Genome Atlas (TCGA) cancer types. Five of 14 cancers showed balanced accuracy of 0.6 or higher: LUSC (0.87), PAAD (0.83), KIRC (0.71), KIRP (0.63) and BRCA (0.60).

**Conclusions:**

This study revealed the significant correlation between prepregnancy maternal obesity and multiomics-level molecular changes in the uHSCs of offspring, particularly at the DNA methylation level. These maternal-obesity-associated epigenetic markers in uHSCs may contribute to increased risks in certain cancers of the offspring. Larger and multicenter cohort validation studies are warranted to follow up the current single-site study.

## Introduction

Maternal obesity has emerged as a primary health concern during pregnancy, with its prevalence alarmingly increasing. According to a study by the Centers for Disease Control and Prevention, the percentage of women experiencing prepregnancy obesity in the United States escalated from 26% to 29% between 2016 and 2019 [1]. The higher birth weight of babies born to mothers with obesity is associated with a higher incidence of childhood cancers such as leukemia and neuroblastoma [2, 3], as well as greater risks of prostate and testicular cancers in men [4–6] and breast cancer in women [7].

Moreover, maternal obesity may have a intergenerational effect and set the stage for increased chronic disease susceptibility later in the adulthood of offspring [8, 9]. The hypothesis of the in utero origin of diseases proposes that numerous chronic diseases have their origins in the fetal stage, the earliest phase of human development [10, 11]. Some researchers have speculated higher stem cell burdens in newborn babies born to obese mothers [12]. Altered hormonal environment and nutrient availability can induce critical changes in fetal stem cells [13], which may predispose these cells to malignant transformation, aligning with the idea of the cancer stem cell hypothesis that cancer cells have stem cell-like properties with an uncontrolled self-renewal program [14–16]. In particular, a study showed increases in cord blood CD34+CD38− stem cell and CD34+ progenitor cell concentrations with maternal obesity [17], suggesting that the higher proportions of stem cells in cord blood may make the babies more susceptible to obesity and cancer risks. However, so far little work provides direct molecular links as to how maternal obesity affects cellular function and increases the disease risk in offspring.

To seek answers in this area, we conducted an epigenome-centered multiomics study to directly pinpoint the effect of maternal obesity in umbilical cord blood hematopoietic stem cells (uHSCs). Epigenetics is chosen as the center of multiomics integration, as it is both inheritable and susceptible to modification by diseases. Thus, it may serve as a plausible mediator in the transmission of the effects of maternal obesity to offspring. We incorporate gene expression for cord blood stem cells and metabolomics data from the cord blood serum as the downstream readout of epigenetics changes. By elucidating these molecular connections, we provide a systematic understanding of how maternal obesity during pregnancy can influence the multiple types of molecular profiles of newborns. Such knowledge may ultimately help develop early therapeutic interventions at the molecular level to mitigate these intergenerational health risks due to maternal obesity.

## Methods

### Overview of the maternal prepregnancy cohort with baby cord blood

In this study, baby cord blood samples from 72 pregnant women (34 obese; 38 non-obese) who delivered at Kapiolani Medical Center for Women and Children in Honolulu, Hawaii (2015–2018) were collected. The study was approved by the Western IRB (WIRB Protocol #20151223). Patients meeting the inclusion criteria were identified from preadmission medical records with prepregnancy BMI ≥30.0 kg/m^2^ (maternal obesity) or 18.5–25.0 kg/m^2^ (normal weight). Pregnant women undergoing elected C-sections at ≥37 weeks gestation were included, to minimize confounding events during labor. Patient exclusion criteria included pregnant women with preterm rupture of membranes, labor, multiple gestations, pregestational diabetes, hypertensive disorders, cigarette smokers, infection of human immunodeficiency virus or hepatitis B virus, and chronic drug use. Demographic and phenotypic information was recorded, including maternal and paternal age, ethnicity, gestational weight gain, gestational age, parity, and gravidity. For newborns, Apgar scores were documented at 1 minute and 5 minutes postdelivery. The Apgar score serves as a comprehensive assessment of a newborn's health, with a normal range considered to be between 7 and 10 [18].

### Sample preparation and methylation profiling

Each baby cord blood sample was collected in the operating room under sterile conditions at the time of the C-section (Pall Medical Cord Blood Collection Kit containing 25 ml citrate phosphate dextrose). The umbilical cord was first cleansed with chlorhexidine swabs before cord blood collection. The total volume of collected blood was measured and recorded before aliquoting to conical tubes for centrifugation. The tubes were centrifuged at 200*g* for 10 min, and plasma was collected. The plasma volume was replaced with 2% FBS/PBS. Negative selection reagents were added to the blood and incubated for 20 min (Miltenyi Biotec, Auburn, CA). The cord blood was diluted with an equal volume of 2% FBS/PBS. A 20 ml aliquot of the diluted blood was layered over a density gradient (15 ml Lymphoprep) and centrifuged at 1,200*g* for 20 min. The top layer containing an enriched population of stem cells was collected, centrifuged at 300*g* for 8 min, and then washed in 2% FBS/PBS. Red blood cells were lysed using ammonium chloride (9:1) with incubation on ice for 10 min, washed twice, and then resuspended in 100 µl of 2% FBS/PBS. Cells were stained with Lineage FITC and CD34 APC for 45 min on ice, washed twice, and then sorted using a BD FacsAria III. Hematopoietic stem cells (CD34+/CD38−/Lin−) were collected and stored at −80°C until DNA/RNA extraction.

DNA and RNA were extracted simultaneously using an AllPrep DNA/RNA extraction kit (Qiagen). DNA purity and concentration were quantified using a Nanodrop 2000 and Picogreen assay. Bisulfite conversion of 500 ng DNA was performed using an EZ DNA Methylation kit (Zymo), followed by sample processing for Infinium HumanMethylation450 bead chips (Illumina) according to the manufacturer’s instructions. Bead chips were analyzed at the Genomics Shared Resource at the University of Hawaii Cancer Center.

### Bulk RNA sequencing

A total of 50 RNA samples were prepared for bulk RNA sequencing. RNA concentration and RIN score were assayed using a Nanodrop 2000 and an Agilent Bioanalyzer. A total of 200 ng of high-quality RNA (RIN ≥7) was subjected to library construction (polyA) and sequenced on HS4000 (2 × 100) at the Yale Center for Genome Analysis, Connecticut to obtain at least 25 M paired reads per sample.

### Methylation data preprocessing

The overall preprocessing workflow is shown in Supplemental Fig. 1. R version 3.6.3 was used for all analyses. As the first step of quality assessment, sex chromosome methylation patterns were analyzed to check for potential sex mismatches between reported and inferred sex using the getSex() function in minfi [19]. No samples with discrepancies between reported and inferred sex were identified or flagged for exclusion Supplemental Fig. 2a. Raw intensity data (.idat) were extracted using the ChAMP package (version 2.16.2) in R with champ.load() function [19–22]. For the filtration step, background controls were subtracted from the data, and raw data that did not pass detection *P*-value of 0.05 were removed. CpG sites whose probes had known underlying SNPs and association with XY chromosomes were removed from analysis due to potential confounding. The quality controls included checking the raw density distribution, multidimensional scaling (MDS) plot, and median intensity values of methylated and unmethylated probes to identify potential outliers or poorly performing samples (Supplemental Fig. 2b–d). After BMIQ normalization using the champ.norm() function [23], the batch effect (including slide and array) due to nonbiological technical variation caused by experiment handling was removed using the ComBat function in the ChAMP package, confirmed by the singular value decomposition (SVD) plot (Supplemental Fig. 2e). A total of 1,992 cross-hybridizing probes were removed using the probe list from ExperimentHub (query id “EH3129”) as reported in Chen et al. [24]. For each CpG site, the methylation score was initially calculated as the β-value, a fluorescence intensity ratio between 0 and 1. To reduce the heteroskedasticity for downstream statistical analysis, the M-values were transformed from β-values using lumi (v.3.1.4) in R [25–28]. A total of 408,773 CpG sites remained for downstream analysis after probe filtering, quality control, normalization, batch correction, and cross-hybridizing probe removal.

### Source of variation analysis and confounding adjustment

To eliminate potential confounding factors of prepregnant maternal obesity among the 13 clinical factors, we conducted a source-of-variation analysis with a collection of ANOVA tests to identify the clinical factors that significantly contribute to the methylation level variation, as done before [29, 30]. The variables with *F* statistics >1 (the error value) were determined as confounders and subjected to confounding adjustment. These factors include the baby’s sex, net weight gain during pregnancy, maternal age, maternal ethnicity, paternal ethnicity, gravidity, and gestational age. To adjust for confounding effects, a multivariate regression model is built using the “limma” package to fit methylation M-values of each CpG site, using the confounding factors above. The remaining residuals on the M-values were considered to be confounding adjusted for the subsequent bioinformatics analysis of DNA methylation. To assess the bias and inflation in the differential methylation (DM) findings, we used Bayesian method “bacon” to calculate the genomic inflation (λ) values before and after the confounder adjustment [31]. Additional surrogate variable analysis (SVA) and randomly shuffled null λ calculations were performed to determine the need for inflation adjustment [32]. The null model λ was 0.96. No surrogate variables were identified for correction in the adjusted model. Thus the observed inflation reflects the true biological signal rather than systematic bias, and no further inflation correction was performed on this confounder adjusted model.

### Bioinformatics analysis of DM

A moderated *t*-test from the “limma” R package (v. 3.42.2) [33] was used to detect DM CpG sites between healthy controls and cases with M-values. The *P*-values were adjusted for multiple hypotheses testing using Benjamini–Hochberg (BH) FDR. CpG sites with FDR <0.05 were considered statistically significant. To minimize the effect of gestational age, CpG sites located within the gestational-age-related differentially methylated regions (DMRs) were removed. A total of 130 DMRs related to gestational age were identified using linear regression analysis performed with bumphunter [34] across eight public datasets including a total of 248 patients: GSE31781 [35], GSE36829 [36], GSE59274 [35, 37], GSE44667 [38], GSE74738 [39], GSE49343 [40], GSE69502 [41], and GSE98224 [42, 43]. A complete list of DMRs is included in Supplemental Table 1. Hypermethylation and hypomethylation states were defined by the values of log_2_ fold change (log_2_FC) of M-values in cases compared to controls: hypermethylation if bigger than 0, and hypomethylation if less than 0. Corresponding genes and feature locations of these differential CpG sites were annotated using IlluminaHumanMethylation450kanno.ilmn12.hg19 (v. 0.6.0) [44].

### KEGG pathway enrichment analysis

The “gometh” function from the R package “missMethyl” (v. 1.26.1) [45–48] was used for KEGG pathway enrichment (RRID:SCR_012773)[49–51] with DNA methylation data. DM sites were used for pathway enrichment within five supergroups from KEGG pathways: Metabolism, Genetic Information Processing, Environmental Information Processing, Cellular Processes, and Organismal Systems. Pathways with adjusted *P*-values less than 0.05 were considered significant. Pathway scores for protein pathways (KEGG: Transcription, Translation, Folding, sorting and degradation) and immune pathways (KEGG: Immune system) were calculated with averaged β-values from the promoter region CpG sites. To validate the enrichment of significant CpGs in specific pathways, we used the hypergeometric test, which calculates the probability of observing *k* or more significant CpGs in a pathway by chance, given the total CpGs on the Illumina array (*N*), the total CpGs in the pathway (*K*), and the total significant CpGs identified in our study (*n*). The formula is: $$P(X \ge k) = 1 - \sum\limits_{i = 0}^{k - 1} {\frac{{\bigg( {\begin{array}{@{}*{1}{c}@{}} k\\ i \end{array}} \bigg)\bigg( {\begin{array}{@{}*{1}{c}@{}} {N - K}\\ {n - i} \end{array}} \bigg)}}{{\begin{array}{@{}*{1}{c}@{}} N\\ n \end{array}}}} $$ where $\frac{a}{b}$ represents the binomial coefficient.

### Weighted coexpression network analysis

Firstly, we adjusted all β-values with clinical confounders, then summarized the DM CpG sites at the gene level by averaging the β-values in the promoter regions (those in the TSS200 and TSS1500 promoter regions). Next, we transformed adjusted β-values to adjusted M-values for the downstream adjacency matrix construction. We used adjusted M-values for the weighted gene coexpression network analysis (WGCNA) with the R package “WGCNA” (v. 1.70–3) [52, 53]. The soft threshold for the weighted adjacency matrix with an adjusted *R*^2^ > 0.8 was 7. The topological overlap matrix was constructed for hierarchical clustering. Modules were identified by the dynamic tree-cut algorithm. The networks were exported to Cytoscape with an edge weight >0.03 in each module. The genes with the highest betweenness and degree in the WGCNA network were identified as the hub genes for different modules.

### Protein–protein interaction network analysis

For the protein–protein interaction (PPI) network analysis, DM genes are used as the inputs and were mapped on the STRING database (v. 10) [54]. Significantly functionally associated protein pairs were identified using PANDA (Preferential Attachment based common Neighbor Distribution derived Associations) (v. 0.9.9) [55]. KEGG pathways associated with these protein pairs (in terms of genes) were found using PANDA. The bipartite network graph was visualized using Cytoscape (v. 3.8.1) [56].

### Stemness score computation

The stemness score was based on Shannon entropy and scaled plasticity, as proposed previously [57]. Shannon entropy has been widely applied in developmental biology, particularly in stem cell research [58–60]. The formulas are shown below:


\begin{eqnarray*}
\begin{array}{@{}*{1}{c}@{}} {\textit{Entropy} = \sum\limits_{i = 1}^N {\frac{{ - \frac{{Cp{G_i}}}{{\sum\limits_{i = 1}^N {Cp{G_i}} }}log\left( {\frac{{Cp{G_i}}}{{\sum\limits_{i = 1}^N {Cp{G_i}} }}} \right)}}{{log(N)}}} }\\
{\rm {\it Stemness\,Score} = \frac{{\mathrm{\it Entropy} - min(\mathrm{\it Entropy})}}{{max(\mathrm{\it Entropy}) - min(\mathrm{\it Entropy})}}} \end{array}
\end{eqnarray*}

*N* is the total number of CpG sites. CpG is represented by the β-value on each CpG probe. The stemness score was calculated for all samples using all remaining 408,773 CpG sites after the preprocessing. A Wilcoxon rank test was performed between the stemness scores of the healthy and maternally obese groups

### Bulk RNA-seq data processing

The Illumina universal adapter regions of raw RNA-seq data were first trimmed using BBMap (v. 38.91, RRID:SCR_016965) [61]. All raw sequences passed the quality control using fastqc (v. 0.11.8, RRID:SCR_014583) [62]. The trimmed .fastq files were aligned by STAR (v. 2.7.0f, RRID:SCR_004463) [63] to the human Ensembl genome (Homo_sapiens.GRCh38.dna.primary_assembly.fa) and Ensembl annotation (Homo_sapiens.GRCh38.94.gtf). The gene expression counts were calculated using featureCount [64] from Subread (v. 1.6.4, RRID:SCR_009803) [65].

### Differential expression (DE) of RNA-seq data

The limma voom transformation was used on RNA-seq data to model the mean-variance relationship of the log_2_ counts [66], supporting the empirical Bayes analysis pipeline in limma (Supplemental Fig. 3a). Source-of-variance analysis was performed to find the clinical confounders with ANOVA tests. The significant confounders included: Maternal_Age, baby sex, hemoglobin, sample group, net weight gain, maternal ethnicity, gravidity, and parity (Supplemental Fig. 3b). The statistically significant DE genes between healthy controls and maternally obese cases were found with confounder adjustment using the “DESeq2” (v. 1.26.0) [67] and “limma-voom” function from the “limma” package [33]. *P*-values were adjusted for multiple hypotheses testing using BH adjustment. No significant differential genes were found with adjusted *P*-values <0.05.

### Correlation analysis between bulk RNA-seq and methylation data

Both methylation and RNA-seq assays were performed on a subset of 47 patients have done . Pearson correlation coefficients (PCCs) were calculated between gene expression and methylation β-values from the promoter regions, among the same patients. As mostly a negative correlation between gene expression and DNA methylation in the promoter region is expected, genes with a high negative correlation (PCC←0.2) were used for pathway enrichment using TOPPFUN [68–70]. Top genes of interest were selected with the absolute value fold change >1.5 in gene expression and gene-methyl correlation ←0.3 for hyper- and hypomethylated CpG sites.

### Metabolomics analysis

Metabolomics data were acquired from a previously published study involving 87 patients in the same cohort from three batches (metabolomics workbench study ID ST001114) [71]. Targeted metabolites were generated with LC-MS, and untargeted metabolites were generated with GC-MS. After the removal of compounds missing in more than 10% of samples, a total of 185 metabolites remained, including 10 amino acids (AA), 40 acylcarnitines (C), 35 acyl/acyl phosphatidylcholines (PC aa), 38 acyl/alkyl phosphatidylcholines (PC ae), and 62 untargeted metabolites. The raw metabolite data were log-transformed, standardized, normalized using variance stabilization normalization (VSN), and batch-corrected with ComBat function in the sva pacakge [72]. Differential metabolites were identified by limma, with clinical confounder adjustment.

### Multiomics integration on metabolomics, epigenomics, and transcriptomics

Matched methylation, gene expression, and metabolomics data are available for a subset of 42 patients. We applied multiomics integration with Data Integration Analysis for Biomarker discovery using Latent cOmponents (DIABLO) implemented in the mixOmics package [73]. DIABLO finds the correlated consensus latent variables among different omics in the supervised manner. Top DIABLO features for each omic were selected based on the loading values. We integrated the pathway-level methylation, gene, and metabolite interactions using pathview [74].

### Evaluation of maternal prepregnancy obesity biomarkers in cancer prediction

We collected Infinium HumanMethylation450 data for a total of 14 cancer datasets (adjacent normal samples >10): BLCA, BRCA, COAD, ESCA, HNSC, KIRC, KIRP, LIHC, LUAD, LUSC, PAAD, PRAD, THCA, UCEC from the Cancer Genome Atlas [75]. In total, 6,428 samples were obtained, consisting of 5,715 tumor samples and 713 adjacent normal tissues.

To build the obesity classification model with maternal obesity biomarkers, we selected 61 hypermethylated CpG sites from the promoter regions of the genes involved in the top five significant pathways based on the missMethyl KEGG enrichment results, namely the cell-cycle, ribosome, nucleocytoplasmic transport, ribosome biogenesis in eukaryotes, and mTOR signaling pathways. We split the 72 maternal obesity and control samples at a ratio of 80/20 with 5-fold cross-validation, then constructed a series of classification methods using the lilikoi R package, where random forest (RF) was the winning model [76, 77]. Next, we applied this RF obesity model to 14 TCGA datasets to perform cancer/normal sample prediction. We report the accuracy, balanced accuracy, and *F*1 score for the model performance as done before [76].

## Results

### Overview of study design and cohort characteristics

**Figure 1:**
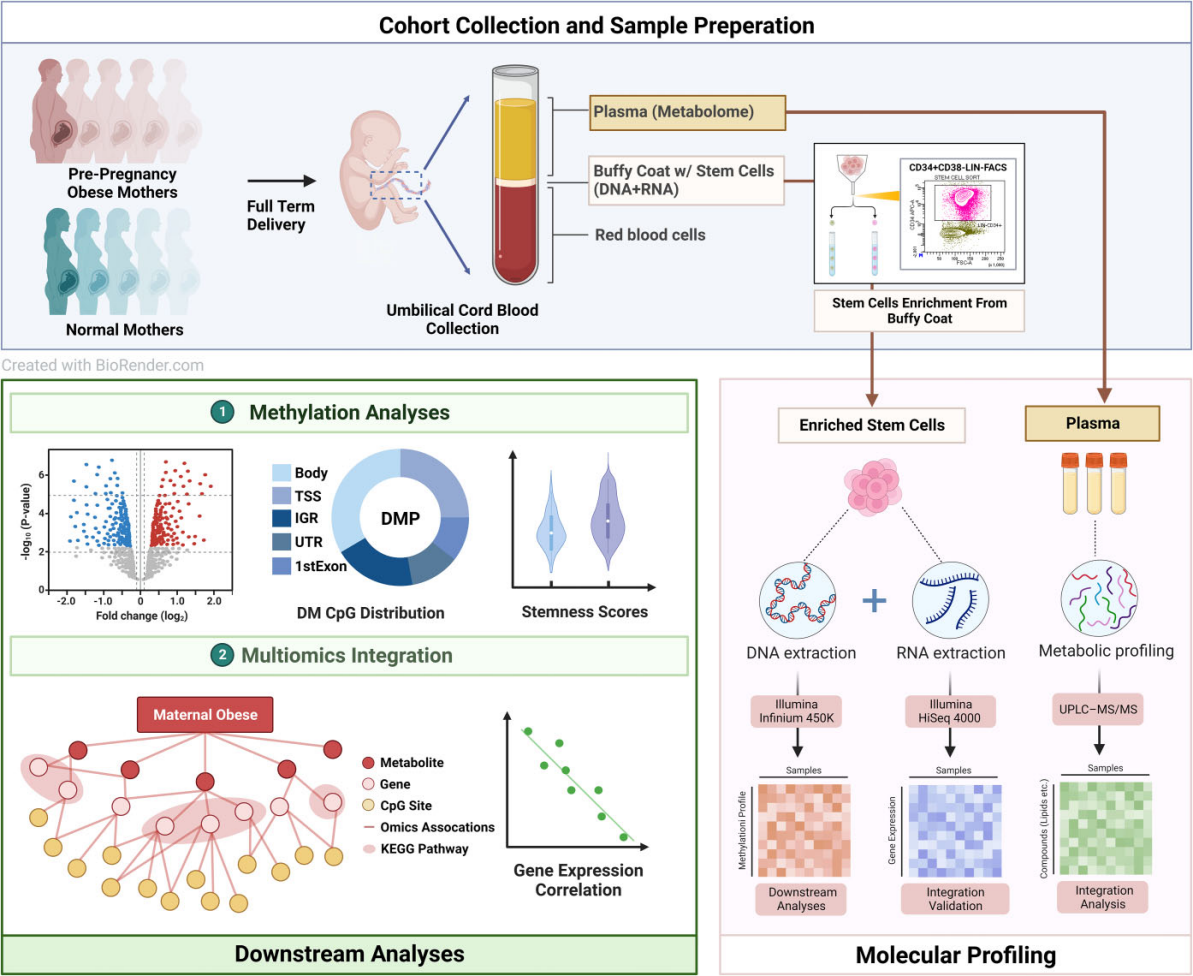
Overview of the study design and analysis. In the preparation step, cord blood plasma samples are collected for metabolome profiling and stem cell sorting. DNA and RNA extraction assays are performed on the enriched stem cells for the methylation and RNA-seq analyses. Downstream analyses are mainly focused on the methylation data. Bulk RNA-seq data were used for validations for methylation discoveries. (Created with BioRender.com)

This study aims to investigate the intergenerational effect of prepregnancy maternal obesity on offspring. A total of 72 patients who elected to deliver full-term babies through C-sections were recruited from Kapiolani Medical Center for Women and Children in Honolulu, Hawaii from 2016 to 2018. This cohort reflects the multiethnic population character of Hawaii, including Asian (*n* = 29), Caucasian (*n* = 15), and Native Hawaiian and Pacific Islanders or NHPIs (*n* = 28). Among them, 38 deliveries are in the healthy control group and 34 are cases with prepregnancy maternal obesity. We excluded natural virginal births, to avoid its potential confounding effect on multiomics profiles. We also carried out stringent recruitment selection criteria, including matching the mothers’ ages as much as possible, as well as similar net gestational weight gain to minimize its confounding effect over maternal prepregnancy maternal obesity. The overall study design is shown in Fig. 1. Briefly, upon collecting the blood samples, umbilical cord blood hematopoietic stem cells (uHSCs) were enriched by FACS sorting with CD34+CD35−LIN− markers (see **Methods**). We extracted DNA and RNA from these uHSCs for Illumina 450k-array-based DNA methylation and bulk RNA-seq sequencing, respectively. The plasma from these cord blood samples was subjected to untargeted metabolomics assays using GC-MS and targeted metabolomics assays using LC-MS [71]. Given the rationale that DNA methylation could be the mediator for exerting the intergenerational effect of maternal obesity [78, 79], we carried out multiomics data integration analysis in the DNA methylation-centric manner.

**Figure 2:**
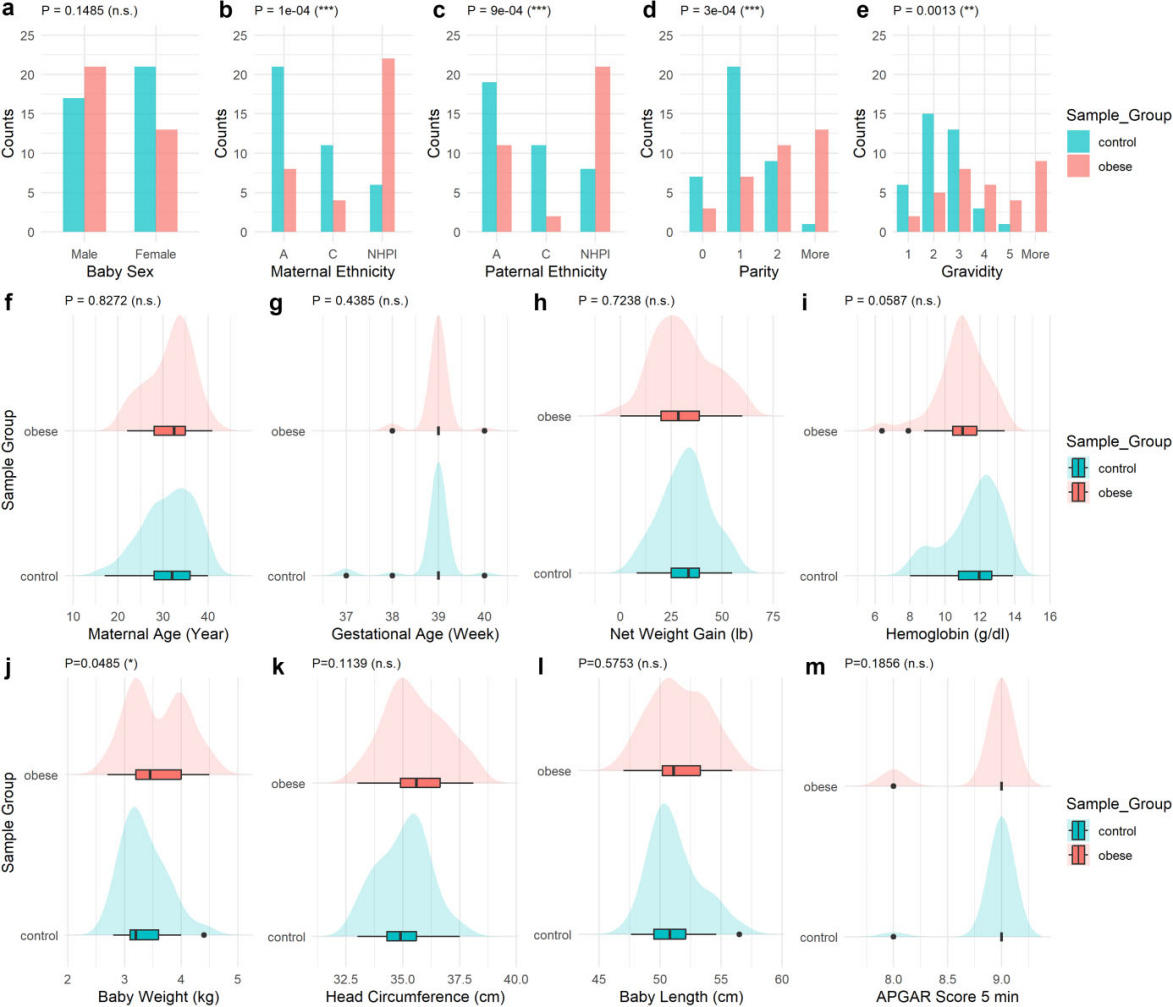
Statistics for mothers and newborns of the multiethnic cohort from Hawaii. (a–e) Categorical variables including baby sex, maternal ethnicity, paternal ethnicity, parity, and gravidity between control and obese groups are shown in the barplots. *P*-values using the chi-square test are annotated comparing control and obese groups. (f–i) The distributions of maternal age, gestation age, maternal net weight gain during pregnancy, and maternal hemoglobin between control and obese groups are compared. Mean and standard deviation are shown in the boxplots. *P*-values using a *t*-test are annotated. (j–m) The distributions of baby weight, baby head circumference, baby length, and APGAR score after 5 minutes of delivery between control and obese groups are compared. Mean and standard deviation are shown in boxplot. *P*-values using *t*-test are annotated.

The demographic details and clinical information of these patients are summarized in Table 1. The distributions of the most representative variables are shown in Fig. 2. Among categorical demographic variables, the distribution of baby sex had no statistical difference between obese and health groups, whereas the ethnicity distributions among mothers and fathers, and parity and gravidity are statistically different (*P* < 0.05) between the two groups (Fig. 2a–e). Besides maternal prepregnancy BMI, other maternal parameters such as maternal age, gestational week, net weight gain, and hemoglobin are also not statistically significantly different between the two groups per study design (Fig. 2f–i, Table 1). While mothers of Asian ethnicity are the majority in the control group, NHPIs account for the majority of the maternal-obese group, revealing the health disparity issue known in the state of Hawaii [80]. Moreover, the control group has lower parities and gravidities, compared with the cases. Babies born to obese mothers show significantly higher (*P* < 0.05) body weights compared with the control group, as expected [81]. Other parameters, including the baby gender, head circumference, body length, and APGAR score at 5 min after birth, are not statistically significantly different between case and control groups (Fig. 2j–m).

**Table 1:** Summary statistics of the study cohort.

	Control (*n* = 38)	Case (*n* = 34)	
Maternal age (years)		31.3 ± 5.6	31.6 ± 4.9
Gestational week		38.9 ± 0.5	39.0 ± 0.3
Net weight gain		32.0 ± 11.6	30.9 ± 14.6
Hemoglobin		11.6 ± 1.6	11.0 ± 1.4
Maternal ethnicity	Asian	21	8
	Caucasian	11	4
	NHPI	6	22
Paternal ethnicity	Asian	19	11
	Caucasian	11	2
	NHPI	8	21
Baby sex	Female	17	21
	Male	21	13
Parity	0	7	3
	1	21	7
	2	9	11
	More	1	13
Gravidity	1	6	2
	2	15	5
	3	13	8
	4	3	6
	5	1	4
	More	0	9

Demographic and clinical statistics are reported for the control and maternally obese groups.

### Global hypermethylation pattern revealed by CpG-level methylation analysis

**Figure 3:**
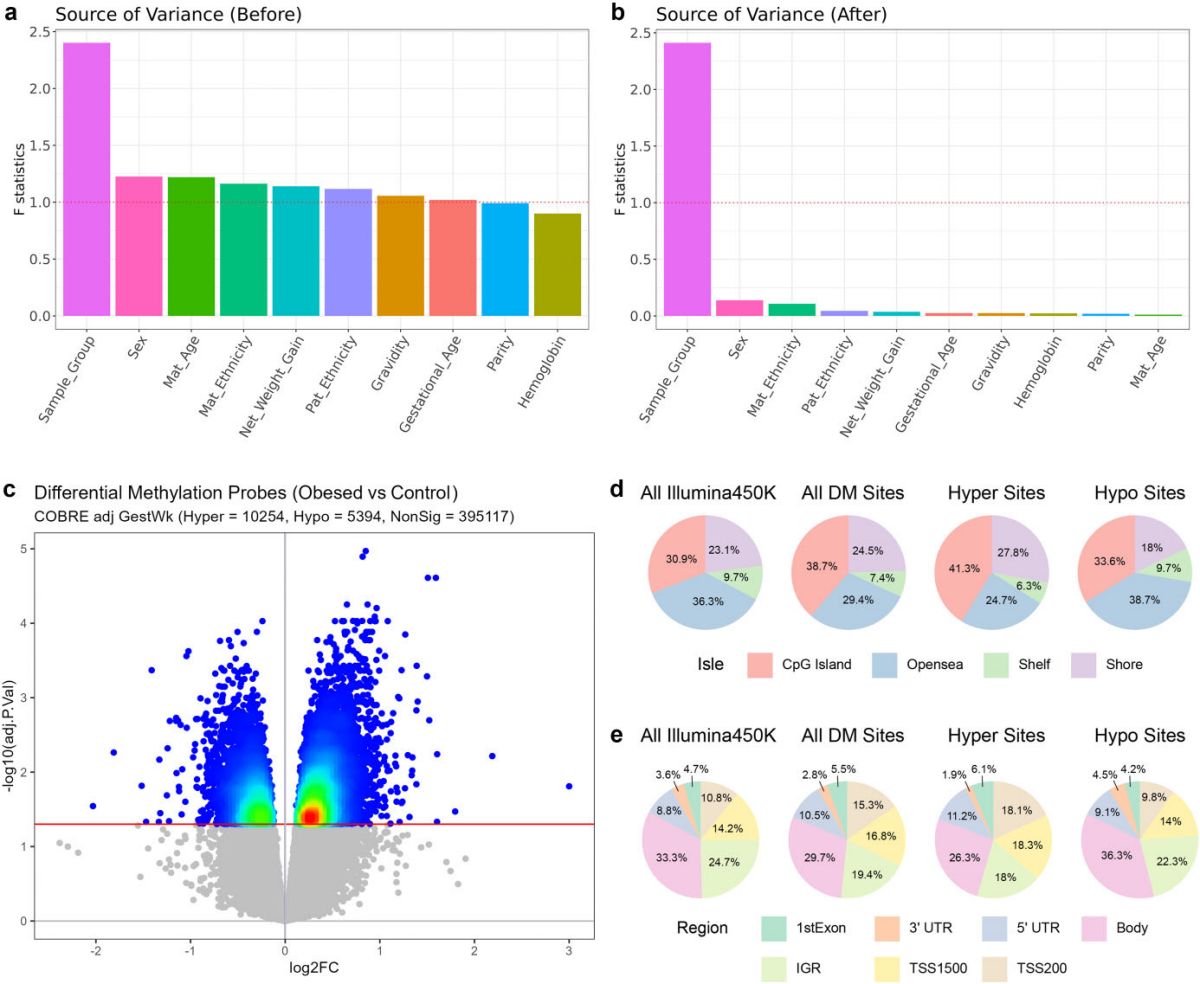
DNA methylation analysis on uHSCs. (a,b) Source-of-variance plot before and after confounding adjustment. *F*-statistics are reported for each clinical factor. *F*-statistics >1 are considered to have confounding effects in addition to the case–control difference due to prepregnancy maternal obesity. (c) Volcano plot of −log(BH adjusted *P*-values) against log FC. The cut-off line for adjusted *P*-value <0.05 is shown as the red horizontal line. The hyper/hypo threshold is shown as a blue vertical line where log FC = 0. Nonsignificant methylation CpG sites after the differential analysis were shown in gray. Significant CpG sites are coloured. (d,e) Normalized location distribution of differentially methylated CpG sites according to their CpG features in terms of isle regions and gene regions based on the chip annotation.

Quality control of methylation data showed no significant sample outliers and no remaining batch effect after ComBAT correction (Supplemental Fig. 2d). For scientific rigor, it is critical to adjust for confounding in DNA methylation association analysis [82]. Thus we performed source-of-variance (SOV) analysis on the β-values of the DNA methylation with respect to physiological and phenotypic information in order to assess potential confounding factors systematically [29, 30, 82]. As shown in Fig. 3a, marginal *F*-statistics in the SOV analysis show that the dominating contribution to DNA methylation variation is maternal prepregnancy obesity status, confirming the quality of the study design which aimed to minimize the effects of other confounders. The other minor confounding factors include baby sex, maternal age, maternal ethnicity, net weight gain during pregnancy, paternal ethnicity, gravidity, and gestational age (*F* > 1). After adjusting these factors by linear regression, all have reduced *F*-statistics of <0.5 (Fig. 3b) except maternal prepregnancy obesity, confirming the success of confounding removal. The quantile–quantile (QQ) plot and genomic inflation factor were used to assess the confounder adjusting model (Supplemental Fig. 4). A decrease in genomic inflation factor (λ) was observed with adjustment of confounders. Although the adjusted model had a λ of 1.28, no surrogate variables were identified for correction in the adjusted model. Therefore the observed inflation reflects mostly the true biological signal, and no further inflation correction was performed.

Next, we conducted DM analysis on the confounding adjusted DNA methylation data (Methods). We observed a global hypermethylation pattern in prepregnancy obese samples, with 10,211 hypermethylated versus 5,362 hypomethylated CpG sites (Fig. 3c). The top 20 differentially hypermethylated and hypomethylated CpG sites are reported in Table 2. These CpG sites are related to a wide variety of biological functions, including inflammation (CD69, ADAM12), transcription factors (ZNF222, HMGN4, LHX6, TAF3), and proliferation and apoptosis (HDAC4, DHRS4, LRCH3, SAFB2, CRADD, EBF3, PRKAR1B). Some top DM CpG sites are directly associated with obesity, including HDAC4 [83] and PLEC1 [84].

**Table 2:** Top 20 hypermethylated CpG sites and top 20 hypomethylated CpG sites.

CpG	Gene	Island	Group	log FC	*P*-value	adj. *P*-value	Type
cg12303247	SYT11	OpenSea	3′UTR	2.188	2.44E−05	6.08E−03	Hyper
cg16818768	PSMG1	Island	TSS1500	1.605	2.15E−05	5.74E−03	Hyper
cg05995465	HDAC4	OpenSea	5′UTR	1.604	1.64E−03	4.64E−02	Hyper
cg01937701	DHRS4	Island	TSS200	1.592	1.95E−10	2.43E−05	Hyper
cg22243583	DLEU1	S_Shore	Body	1.522	2.53E−06	2.00E−03	Hyper
cg16927136	RPL35A	OpenSea	TSS1500	1.507	2.38E−10	2.43E−05	Hyper
cg08899199	ST7	S_Shore	Body	1.4	7.32E−07	1.12E−03	Hyper
cg05054115	DHRS4	Island	TSS200	1.389	6.64E−08	3.71E−04	Hyper
cg12878710	LRCH3	Island	TSS200	1.387	1.26E−06	1.47E−03	Hyper
cg05130022	HMGN4	N_Shore	TSS200	1.386	1.51E−04	1.45E−02	Hyper
cg05643303	HOXC8	Island	TSS200	1.345	2.69E−05	6.34E−03	Hyper
cg07449543	CHORDC1	S_Shore	TSS200	1.342	6.31E−05	9.53E−03	Hyper
cg25016112	DENND3	OpenSea	Body	1.314	1.22E−03	4.00E−02	Hyper
cg09552166	MSL2	N_Shore	TSS200	1.296	2.29E−05	5.92E−03	Hyper
cg01003902	SAFB2	Island	TSS200	1.269	1.03E−08	1.41E−04	Hyper
cg11028445	FAM96A	N_Shore	TSS1500	1.265	1.97E−04	1.65E−02	Hyper
cg10317138	ADAM12	N_Shore	Body	1.229	5.05E−04	2.60E−02	Hyper
cg09757277	ZNF222	S_Shore	5′UTR	1.229	9.86E−08	4.24E−04	Hyper
cg04117338	CRADD	N_Shore	5′UTR	1.209	1.66E−03	4.67E−02	Hyper
cg07354583	CD69	OpenSea	Body	1.205	5.93E−07	1.01E−03	Hyper
cg04043455	EBF3	S_Shelf	Body	−2.031	6.11E−04	2.86E−02	Hypo
cg20784950	PLEC1	N_Shore	Body	−1.812	1.96E−05	5.45E−03	Hypo
cg09976051	AGA	N_Shore	Body	−1.516	1.67E−04	1.53E−02	Hypo
cg13862711	LHX6	Island	Body	−1.469	1.65E−03	4.65E−02	Hypo
cg16434331	SLC39A11	OpenSea	Body	−1.411	9.50E−08	4.24E−04	Hypo
cg05636467	EBF3	S_Shelf	Body	−1.335	1.65E−03	4.65E−02	Hypo
cg16858146	TAF3	S_Shelf	Body	−1.33	3.14E−05	6.80E−03	Hypo
cg24796644	MDGA1	Island	Body	−1.242	1.47E−05	4.79E−03	Hypo
cg11064039	PRKAR1B	Island	5′UTR	−1.227	1.58E−03	4.56E−02	Hypo
cg06833656	TBCD	OpenSea	Body	−1.219	2.67E−06	2.05E−03	Hypo
cg25430507	NXPH2	S_Shore	TSS1500	−1.152	2.08E−06	1.87E−03	Hypo
cg03485608	NXPH2	N_Shore	Body	−1.152	2.71E−06	2.05E−03	Hypo
cg00928596	MIR365−1	OpenSea	TSS200	−1.148	7.31E−05	1.03E−02	Hypo
cg12601963	NCRNA00200	Island	Body	−1.132	2.57E−06	2.02E−03	Hypo
cg22772691	SLC12A7	S_Shelf	Body	−1.123	1.79E−04	1.57E−02	Hypo
cg02584267	EBF3	OpenSea	Body	−1.121	2.38E−04	1.80E−02	Hypo
cg19282259	NCRNA00200	Island	TSS200	−1.104	3.48E−06	2.32E−03	Hypo
cg08010094	NXPH2	S_Shore	TSS1500	−1.094	1.04E−03	3.69E−02	Hypo
cg06916001	MIR365−1	OpenSea	TSS200	−1.088	5.73E−05	9.17E−03	Hypo
cg03721387	KRTAP24−1	OpenSea	3′UTR	−1.04	4.29E−06	2.53E−03	Hypo

log FC, *P*-values, BH adjusted *P*-values, and CpG annotations are reported for the top 20 differentially hypermethylated CpG sites ordered by the adjusted *P*-values by “limma” packages. Hypermethylated CpG sites are defined as log FC > 0, whereas hypomethylated CpG sites are defined as log FC < 0.

We further examined the distributions of these differentially methylated sites, relative to the CpG island regions and promoter proximity (Fig. 3d,e). A big fraction (42.2%) of the DM sites are located in CpG islands [85, 86], significantly higher than that from the Illumina 450 K annotation (*P* < 2E−16). CpG islands are more frequent in the hypermethylated sites (43.8%) than in the hypomethylated sites (39.1%), which is consistent with the global hypermethylation pattern. Relative to gene localization, DM sites are most frequent (39.3%) in the promoter regions (including 18.7% and 20.6% in TSS200 and TSS1500, respectively) as expected.

### Functional analyses reveal the association between maternal obesity and cell-cycle, immune function, and metabolic changes in the cord blood of offspring

To investigate the biological functions related to the epigenome alternation, we conducted systematic analysis of DM sites by multiple methods: KEGG pathway enrichment analysis, Weighted Gene Co-expression Network Analysis (WGCNA), and PPI network analysis.

**Figure 4:**
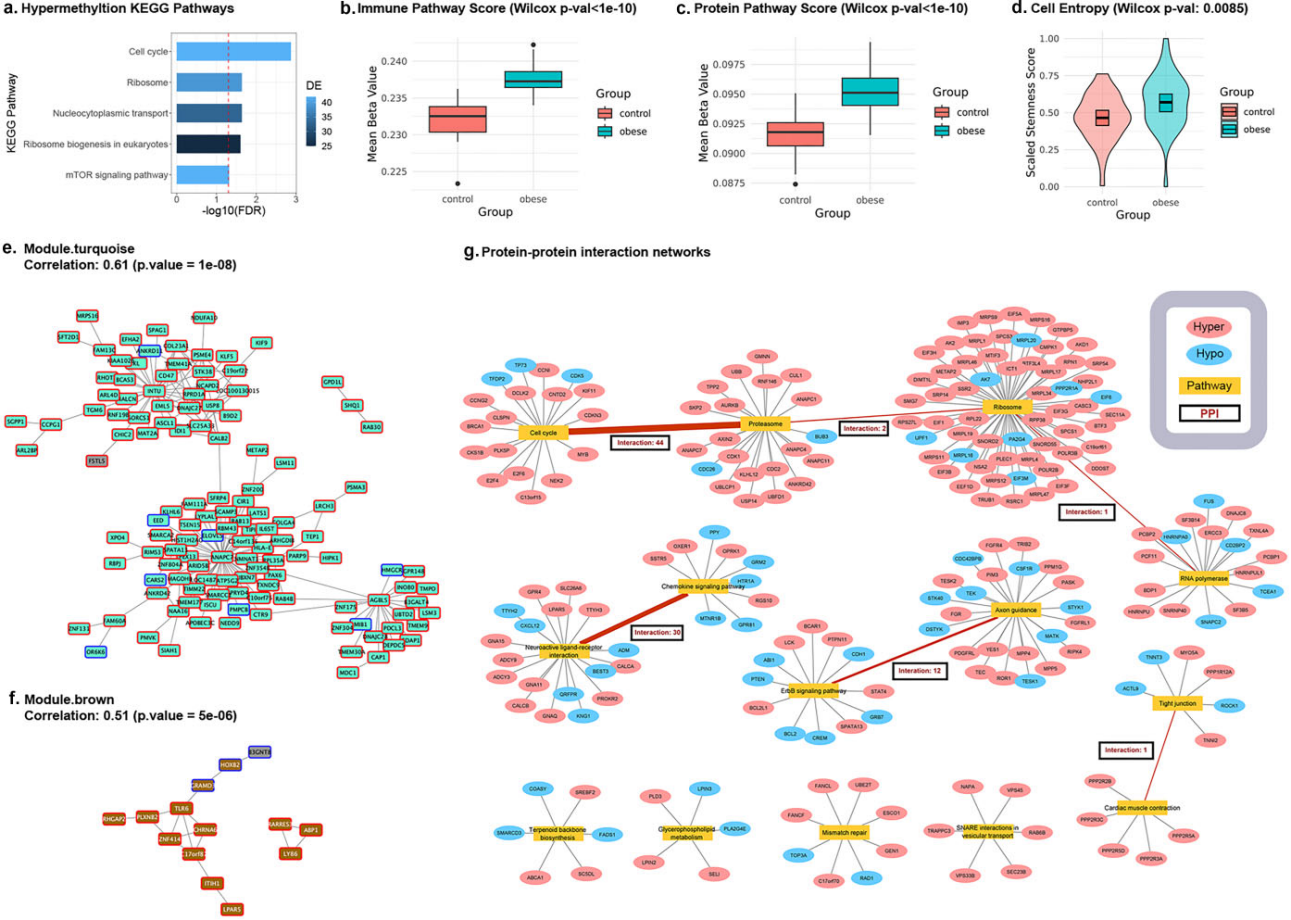
Pathway and network analysis.(a) KEGG pathway enrichment for hypermethylated CpG sites from the promotor region. Enriched KEGG pathway names, adjusted *P*-values (−log 10 transformed), and the size of enriched gene list are reported for CpG sites from TSS200+TSS1500 regions. The red dotted line shows the threshold cut-off for FDR at −log_10_(0.05). (b,c) Violin plots of averaged β-values for KEGG protein pathway collection and immune pathway collection with Wilcoxon *P*-values. (d) Violin plots of cell entropy scores between control and obese groups with Wilcoxon *P*-values. (e,f) WGCNA network analysis results. WGCNA modules are shown for both the control and the obese group. The top two modules with the largest degrees are turquoise and brown modules. Each node represents a gene. Genes coexpressed in each module are annotated. (g) Protein-protein interaction (PPI) network. Bipartite graphs represent enriched KEGG pathways and associated genes with significant PPIs. Red nodes represent genes with hypermethylated CpG sites. Blue nodes represent genes with hypomethylated CpG sites. Yellow nodes represented the enriched KEGG pathways. Number of interpathway PPIs are annotated in the rectangular boxes.

KEGG pathway enrichment analysis on hypermethylated CpG sites identified five significant pathways with hypergeometric FDR < 0.05 (Fig. 4a), namely, the cell-cycle, ribosome, nucleocytoplasmic transport, ribosome biogenesis in eukaryotes, and mTOR signaling pathways. The cell-cycle, ribosome, and nucleocytoplasmic transport pathways are essential to normal cell functioning. mTOR signaling pathway coordinates the nutrient-mediated metabolism, immune responses, and cell-cycle progression, and dysregulation of mTOR could lead to various diseases such as cancer and obesity [87]. There was no significantly enriched pathway emerging from hypomethylated CpG sites. The maternally obese group shows significantly higher methylation levels in KEGG protein synthesis and immune system pathway collections compared with the control group, indicating repression in immune response as well as translation and protein synthesis (Fig. 4b,c). Similarly, we further explored the differential potential, or stemness, of uHSCs. We first confirmed the homogeneity of uHSCs by single-cell RNA sequencing UMAP plot (Supplemental Fig. 5). We calculated the cell stemness scores using the DNA methylation β-values similar to others [88]. uHSCs derived from the maternally obese group exhibit significantly elevated stemness scores (*P* < 0.01) in comparison to the control group (Fig. 4d), confirming the results of the KEGG pathway analysis.

Next, we applied WGCNA to cluster coregulation of gene-level methylation, by averaging CpG sites to affiliated genes (see Methods). Five coexpression modules are identified, using the M-values adjusted for clinical confounders (Supplemental Fig. 6a), and all modules show positive correlations with maternal obesity except one. The largest turquoise module (Fig. 4e) is related to the cell-cycle, protein synthesis, and transport and vesicle trafficking pathways through pathway enrichment analysis. Some hub genes in this module are identified, including INTU, ANAPC7, and AGBL5. These genes were reported to be essential for maintaining cell polarity (INTU) [89], proliferation (ANAPC7) [90] and glycemic control (AGBL5) [91]. The brown module (Fig. 4f) is enriched with immune response pathways, in which TLR6 is identified as a hub gene. The other yellow module is related to ion homeostasis, and the gray module is related to the p53 pathway, apoptosis, cell senescence, and ER stress (Supplemental Fig. 6b). The only negatively correlated blue module is associated with axon guidance and the VEGF signaling pathway (Supplemental Fig. 6b).

Furthermore, we examined the PPI network, using the gene-level DNA methylation as surrogates (Fig. 4g). The PPI analysis identifies 14 unique pathways (FDR < 0.05) predominantly associated with hypermethylated CpG sites in the TSS200 and TSS1500 regions. The top five largest pathways included ribosome, proteasome, cell-cycle, axon guidance, RNA polymerase, and neuroactive ligand–receptor interactions. Taken all three types of systematic analyses together, cell-cycle, immune function, and protein synthesis are ubiquitously highlighted, suggesting that these biological functions in cord blood stem cells are negatively impacted by maternal obesity.

### Multiomics analysis reveals disruptions in cell-cycle and metabolic pathways

**Figure 5:**
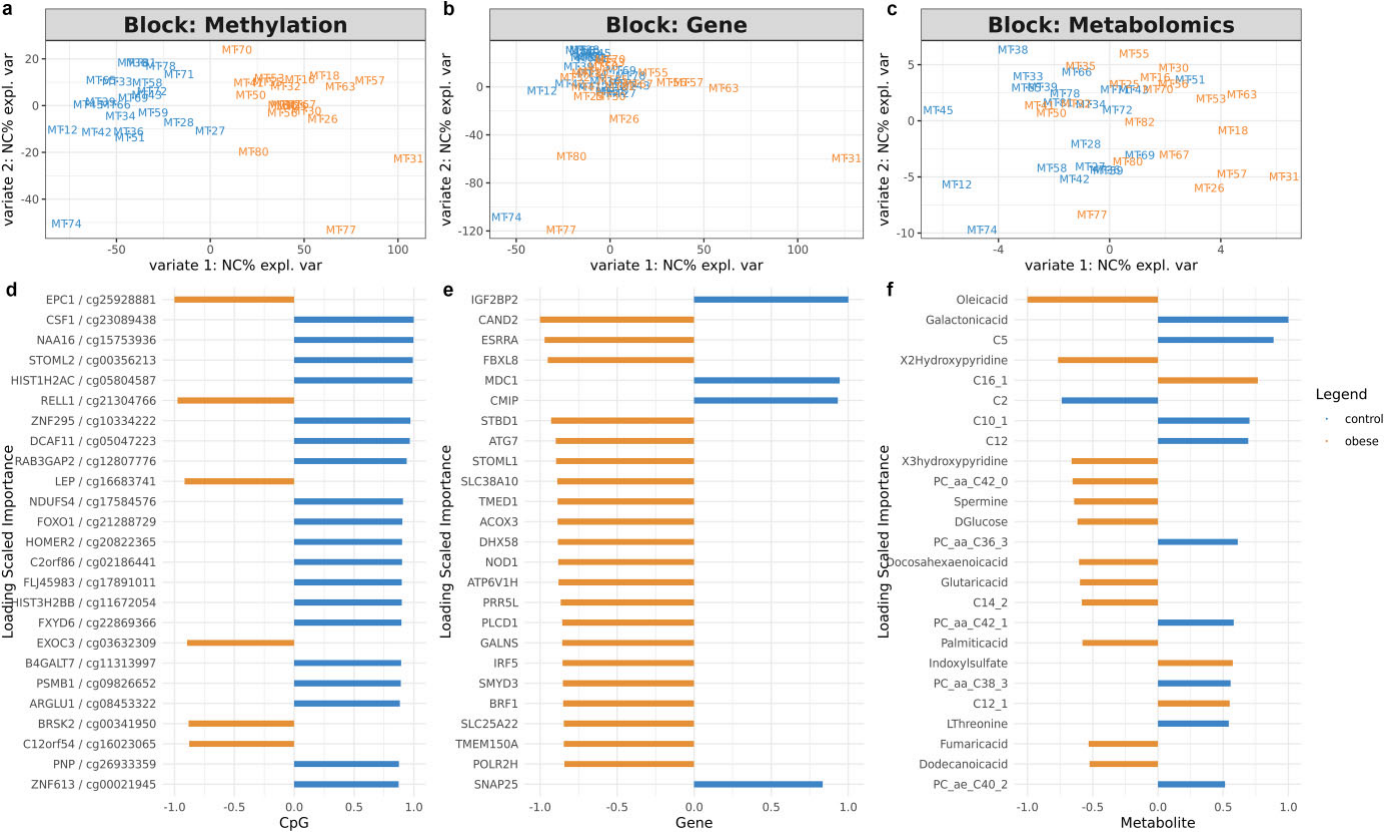
Multiomics integration analysis. (a–c) Omics-specific sample plots from DIABLO showing the separation of obese and control samples in methylation data, gene expression data, and metabolomics data, respectively. (d–f) Importance plot of top 25 features in methylation, gene expression, and metabolomics modalities with the highest loadings extracted from the embedding space. The color represents the condition whose features contribute the most.

To systematically investigate the epigenetic, transcriptomic, and metabolomic alterations induced by maternal obesity, we performed multiomics integration analysis on this cohort. We employed DIABLO, a supervised integration method that extracts features associated with maternal obesity, based on the correlations in the embedding space [73]. Figure 5a–c shows that methylation data provide the clearest separation between obese and control groups, confirming the value of the earlier DNA methylation-centered analysis.

The top 25 features from each omic with the highest feature weights (loadings) following integrated canonical correlation analysis are demonstrated in Fig. 5d–f. The methylation features with the highest weights related to maternal obesity include CpG sites involved in cell-cycle control, glucose metabolism, and adipogenesis (FOXO1 [92]), DNA repair (LIG3, SMUG1), erythropoietin pathway and differentiation (EPO, CSNK2A1, CSF1), which are hypermethylated in the obese group. Hypomethylation of LEP (encoding leptin) was also observed as a top feature, aligning with previous findings that maternal obesity is associated with elevated maternal leptin levels, a known marker of adipose tissue [93]. These featured CpG sites indicate repression in fat metabolism and DNA repair and reduced differentiation potential. In the transcriptomic space, many genes related to mRNA splicing (SRRM1, IGF2BP1, IGF2BP2, CNOT4) have increased expression levels due to maternal obesity. Among the metabolite features, essential sugars (glucose, xylose), polyunsaturated fatty acids (oleic acids, DHA, arachidonic acid), and phosphatidylcholine (PCs) are mostly decreased in the obese group, whereas most acylcarnitines (C) are elevated. The metabolic changes show an overall accumulation of saturated fatty acid, but repression of fat breakdown, glucose, and unsaturated fatty acid generation. As polyunsaturated fatty acids (e.g., arachidonic acid) have important anti-inflammatory effects, the results indicate a proinflammatory environment in offspring born of prepregnant obese mothers.

### The maternal obesity classification model is predictive of KIRC, LUSC, and PAAD cancers in TCGA

**Figure 6:**
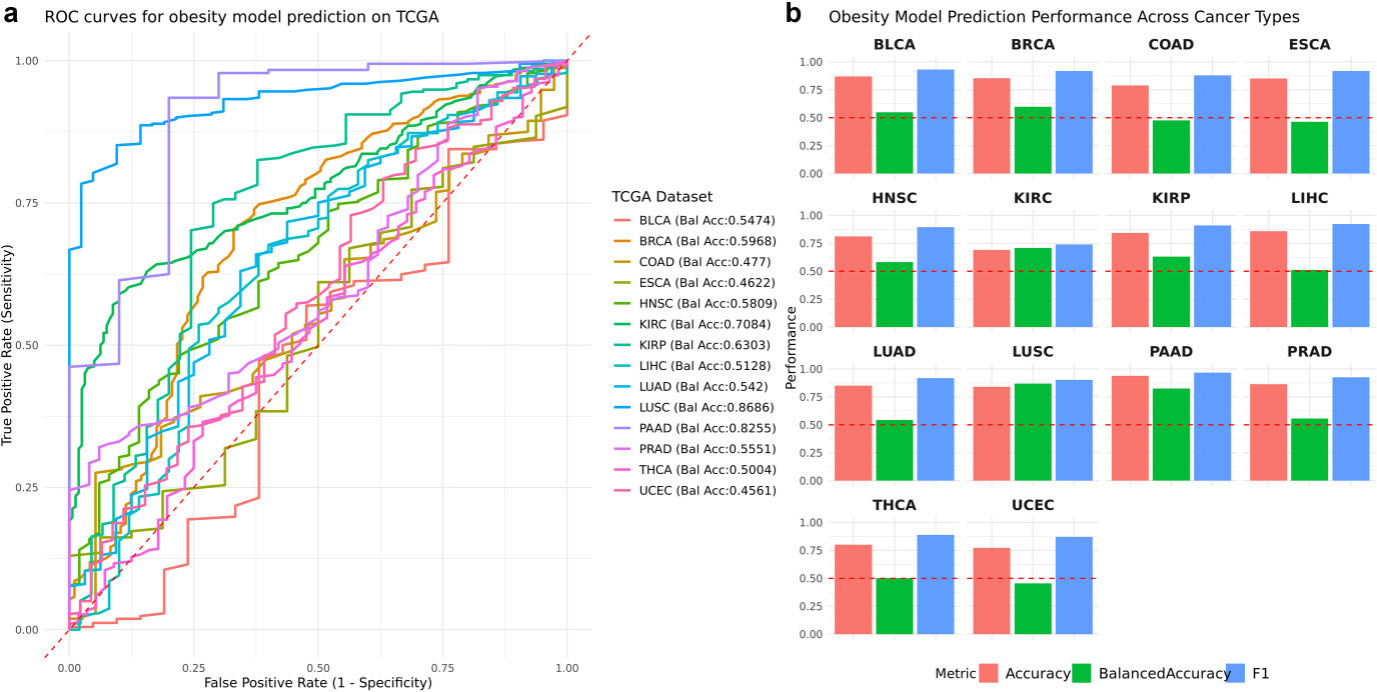
TCGA cancer classification by the maternal obesity classification model. (a) Receiver operating characteristic (ROC) curves on the 14 TCGA cancers, using the random forest classification model built with the CpG sites of genes in the top 5 pathways associated with maternal prepregnancy obesity. Balanced accuracy is shown for each cancer type. (b) Barplots showing the prediction performances on these TCGA datasets, based on AUC, balanced accuracy, and *F*1 score.

Maternal prepregnancy obesity may predispose a higher risk of cancer and other diseases in babies’ later life, via epigenetic modification [12, 17]. To check this assumption, we built maternal obesity random forest classification models using a total of 63 hypermethylated promoter region marker CpG sites obtained from the top KEGG pathways which overlapped with the 14 TCGA cancer data that had sufficient numbers (*n* > 10) of adjacent normal samples (Supplemental Table 2). The maternal obesity random forest model resulted in balanced accuracy of 0.93 on the obesity training data. We then applied this obesity classification model to predict the known adjacent normal and tumor tissue labels from DNA methylation data of 14 TCGA cancers, each of which has sufficient (*n* > 10) tumor-adjacent normal samples (Fig. 6). This allows us to assess if the maternal obesity DNA methylation markers are associated with cancers. As shown in Fig. 6a, three cancer types have good prediction balanced accuracy (Bal acc) of at least 0.7: LUSC (0.87), PAAD (0.83), and KIRC (0.71); and two additional cancers reached 0.6: BRCA (0.60) and KIRP (0.63). The other metrics, including overall accuracy and *F*1 scores are shown in Fig. 6b. Thus, these results show that the epigenetic markers of CpGs associated with maternal obesity are also potentially associated with tumorigenesis in lung, breast, pancreas, and kidney. This finding gives a preliminary indication that maternal prepregnancy obesity may predispose offspring to increased risks in certain cancers later in life through epigenetic modifications.

## Discussion

Maternal obesity is one of the most urgent health concerns worldwide. Prepregnancy maternal obesity can cause various pregnancy-related complications and predispose offspring to cardiometabolic complications and chronic diseases in the long term [9]. Multiple cross-continental large-cohort meta-analyses have shown that maternal obesity is directly associated with the offspring’s risk of obesity, coronary heart disease, insulin resistance, and adverse neurodevelopmental outcomes based on longitudinal observational studies [9, 94, 95]. To directly pinpoint the molecular level changes in offspring by maternal prepregnancy obesity, we used cord blood stem cells as the study material, which serve as an excellent surrogate to reveal the newborn's metabolism and immune system changes at the time of birth [96]. The current study expands on previous effects and investigates the direct impact of maternal obesity on uHSC programming, the progenitor of the immune cell population, using a multiomics (epigenetic, gene expression, and metabolite) analysis approach from a unique multiethnic cohort.

Centered around methylation changes, three complimentary functional analysis approaches (KEGG, WGCNA, and PPI) consistently demonstrated that maternal obesity impacts multiple biological functions including hypermethylation in promoters of genes involved in the cell-cycle, ribosome biogenesis, and mTOR signaling pathways. Moreover, the mTOR signaling pathway also plays a crucial role in metabolism and cell-cycle regulation; disruption of this pathway leads to insulin resistance and long-term diseases [97]. We observed a significant increase in stemness scores among uHSCs affected by maternal obesity, aligning with the expected downregulation in cell-cycle gene expression due to observed hypermethylation in the promoters of these genes. Higher stemness scores indicate enhanced quiescence, shifting the balance between stem cell maintenance and differentiation towards the former. Unlike adult HSCs, fetal/neonatal HSCs typically exhibit higher proliferation and self-renewal capabilities, crucial for blood cell regeneration and innate immune system development [98]. Our findings provide strong epigenetic evidence that maternal obesity compromises the maturation processes in neonatal uHSCs, which may predispose newborns to immunological disorders.

**Figure 7:**
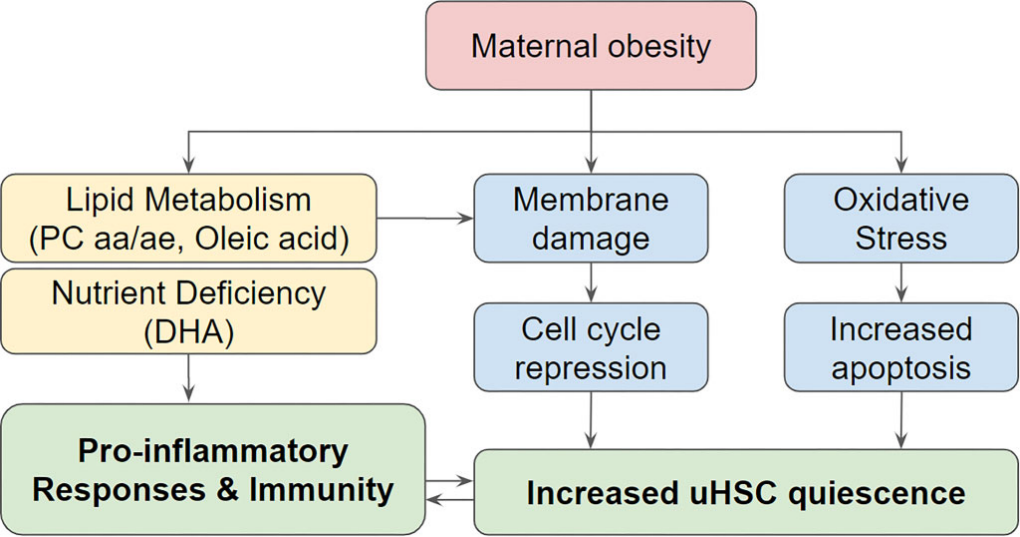
A proposed conceptual model of the impact of maternal obesity on neonatal development.

The subsequent multiomics integration analysis expanded conclusions from methylation analysis to additional metabolomics readouts that are also linked to biological functions, e.g., cell-cycle and inflammatory pathways. We thus propose a conceptual model to illustrate the effect of maternal prepregnancy obesity (Fig. 7). Maternal obesity leads to nutrient deficiency with lower levels of essential amino acids and fatty acids in the newborn blood and disrupts the lipid metabolism homeosis in offspring. These metabolite changes further induce cell membrane instability and repress cell-cycle progression and cell proliferation [99], enhancing the dysregulation of these functions pre-existing at the methylation level. Lipid dysregulation may also enhance the proinflammatory environment, which in turn induces complications in offspring later in life, such as cardiovascular disease. Such a proposed model is also consistent with and further strengthens previous studies at the metabolomics or epigenome levels. For example, previous metabolomics studies of cord blood showed that metabolic derangement predisposes newborns to cardiometabolic and endocrine diseases, and disrupts the normal hormone function and neonatal adiposity [93, 100]. A previous epigenome-wide association study (EWAS) with cord blood found a strong association between DNA methylation pattern and postnatal BMI trajectory until adolescence [101].

We also tested if maternal prepregnancy obesity can provide quantitative support to the long-speculated theory of the in utero origin of cancers [10, 11]. In particular, some researchers have hypothesized that there exist higher stem cell burdens in newborn babies born to obese mothers [12]. Here we provide evidence that such stem cell burden is highly likely due to intergenerational DNA methylation modification on some key biological functions (cell cycle, ribosome function, and immune response) in the uHSCs. We built a random forest model trained on 61 maternal-obesity-associated CpG markers in uHSCs and applied it to predict tumor and normal tissues across 14 TCGA cancer types, without previous cancer-specific training. This model achieved decent balanced accuracy above 0.6 for 5 out of 14 TCGA cancers investigated: LUSC, PAAD, BRCA, KIRP, and KIRC, reflecting its cross-context predictive potential. These cancers, characterized by inflammation, immune dysregulation, and epigenetic disruption, align with pathways enriched in the obesity-associated markers [102, 103]. Uncontrolled cell division, immune evasion, and chronic inflammation are well-established hallmarks of cancer [104], and these featured 61 CpG sites were implicated in relevant biological pathways that were intimately connected with cancer development. Previous results revealed a significant increase in stemness scores among uHSCs affected by maternal obesity, which aligns with the observed hypermethylation and subsequent downregulation of key cell-cycle genes. This heightened stemness may predispose these cells to malignant transformation if these epigenetic modifications persist, leading to an elevated stem cell burden, disrupting normal cell-cycle control, weakening immune surveillance, and ultimately increasing susceptibility to cancer. While performance was lower for other cancers, likely due to the small sample size limiting the detection of additional CpG biomarkers, tissue-specific methylation variability, and microenvironment differences [105], the maternal obesity model implicates its biological relevance across diverse cancers.

This study is the first to directly examine the granular changes in the stem cell population of cord blood from babies of prepregnant obese mothers using a multiomics approach. Previously, the association between maternal obesity and epigenetic modifications has been investigated across various tissue types (e.g., adipose tissue, liver, cord blood) and species (e.g., human, mouse) [106]. We cross-checked our findings with these reports, many of which align, providing further validation of their biological significance. For example, 33 CpGs across 20 genes, such as those in TAPBP (cg17621507, cg23922433, cg27385940), TNFAIP8 (cg18689486, cg07376834, cg03723497,cg21130861), and AGPAT1 (cg09043226, cg25733934, cg08049198, cg18191873) in our study are consistent with the cord blood leukocyte DNA methylation study from Martin et al. with the same study objective [107]. TAPBP, TNFAIP8, and AGPAT1 play roles in immune function, transcriptional regulation, and lipid metabolism, respectively. Additionally, our analysis also identified different CpG probes within the same genes previously associated with maternal obesity, offering additional insights into their epigenetic regulation. For instance, we observed different CpGs in HLA-E (cg01462744, cg02942965, cg26175526), ALPK1 (cg04779144, cg10855342), and PTEN (cg09472211). These genes were also reported from the Boston Birth Cohort study [108]. We identified different hypermethylation sites on MYT1L (cg05786278, cg17519749, cg21239227) and IGF1R (cg01284192, cg06596307, cg08138544, cg16918683, cg26577252) genes; these genes also showed high methylation levels in the cord blood (on different CpG sites) reported by Josefson et al. [109]. Additionally, in our gene expression and methylation correlation analysis of uHSC, HOXA9 and HOXA5 emerged as the top genes (Supplemental Fig. 3d), displaying strong correlations between expression and methylation levels. These hypomethylated genes (HOX family genes), along with 25 additional commonly identified genes, are consistent with the finding in the DNA methylation study on leukocytes of cord blood samples [110], linking them to maternal lipid and cholesterol levels. In our study, HDAC4 and PLEC1 stand out for their strong associations with obesity-related traits among the top differentially methylated CpG sites. Hypermethylation of cg05995464 in HDAC4 was previously reported to be associated with childhood obesity [111]. PLEC1 is a critical gene for extracellular matrix remodeling in adipose tissue, and hypomethylation of cg20784950 in PLEC1 is evident in our study. Lower PLEC1 methylation was previously correlated with higher BMI and obesity status [84, 112]. Together, these comparisons underscore the robust and overlapping epigenetic patterns associated with maternal prepregnancy obesity.

There are some caveats of this study related to the study design. First, this is a single-site study with a relatively small sample size, and along with some genomic inflation, the statistical power of the findings is limited. This is especially the case for the gene expression omic layer, where individual DE genes are lacking. This may have limited maternal obesity CpG biomarker identification, which resulted in positive risk associations in some, but not all, of the 14 TCGA cancers in the classification model (Fig. 6). When the budget allows, a large-scale multisite population study would be desirable. Secondly, we use the stem cell population in the cord blood as the surrogate for “stemness” property investigation, to link the offspring’s disease with maternal obesity. It is most feasible and practical to collect cord blood cells, and the painstaking measurements of the uHSC population avoid blood cell type heterogeneity issues, which may confound the cord blood DNA methylation result significantly [82]. However, this approach may very well be simplified and biased, as stem cells exist in many body parts of babies. Therefore, extrapolations from uHSCs need to be cautioned. Further, our phenotypic data collection focused on the physiological and demographic information and missed socioeconomic data. Thirdly, environmental, lifestyle, or social determinants may act as confounders and influence the observed outcomes, which are not measured or adjustable in the study, per the protocol. Some of these measurements, such as lifestyle and health insurance, can be mitigated by incorporating electronic health record data, similar to what we have done [113, 114]. Additionally, an important aspect of a maternal-offspring study is to longitudinally follow up these offspring for health outcomes later in life. The IRB approval for this study was unfortunately not designed for such an investigation. Nevertheless, this uHSC multiomics study provides a critical initial lens through which to investigate immunometabolic mechanisms, and could serve as the foundation for possible future work mentioned above.

## Conclusion

This study of newborns demonstrates the direct impact of maternal prepregnancy obesity on newborn blood at the multiomics level, which includes increased cell-cycle arrest, impairment in the uHSC differentiation capacity, increased inflammation, and disruption of lipid metabolism. We also showed that maternal-obesity-associated epigenetic modifications are closely related to cancer markers, which could potentially help mitigate intergenerational health risks.

## Disclosure of use of AI-assisted tools including generative AI

During the preparation of this work the authors used GPT-4.0 to improve the readability. Prompts used in GPT-4.0 include “help me improve my writing in a more logical and professional way” and “help me correct the grammar” along with a paragraph of the authors’ own writing. After using this tool/service, the authors reviewed and edited the content thoroughly and take full responsibility for the content of the publication.

## Availability of Source Code and Requirements

Project name: COBRE Hawaii Maternal Obesity Study

Project home page: https://github.com/lanagarmire/COBRE_methyl

Operating system(s): Windows, macOS, Linux

Programming language: R, Python

Other requirements: R ≥ 4.1.0

License: GNU-GPL-3.0

Code to produce the analyses in this manuscript is also available through the project homepage on github.

## Supplementary Material

giaf039_Supplemental_Files

giaf039_Authors_Response_To_Reviewer_Comments_Original_Submission

giaf039_GIGA-D-24-00597_original_submission

giaf039_GIGA-D-24-00597_Revision_1

giaf039_Reviewer_1_Report_Original_Submission Anne Bozack -- 1/16/2025

giaf039_Reviewer_2_Report_Original_Submission Christian M Page, Ph.D. -- 1/30/2025

## Data Availability

DNA methylation data and bulk RNA-seq data generated in this study have been submitted and are available through the National Institutes of Health Gene Expression Omnibus (GEO) with the accession number GSE273075. Other datasets used in this project for the analysis and validation purpose are publicly available. The placenta datasets used in this article are available in the GEO repository with accession numbers GSE31781, GSE36829, GSE59274, GSE44667, GSE74738, GSE49343, GSE69502, and GSE98224. Cord blood metabolomics data used in this article are available in the Metabolomics Workbench with study ID ST001114. Cancer methylation datasets for BLCA, BRCA, COAD, ESCA, HNSC, KIRC, KIRP, LIHC, LUAD, LUSC, PAAD, PRAD, THCA, and UCEC are available in The Cancer Genome Atlas [119]. Supporting data, including gestational DMR regions, obesity classification marker list, TCGA clinical information, and an archival copy of the code, are also available via the GigaScience database GigaDB [115].

## Additional Files

**Supplemental Figure 1**. Data processing workflow. The complete data preprocessing procedures consisted of sex-mismatch check, filtration, quality-control checks, normalization, batch correction, singular value decomposition analysis, and conversion of β-values to M-values. Created with BioRender.

**Supplemental Figure 2**. Data quality control. (a) Sex mismatch check. (b) Median intensity plot. (c) MDS plot for top 1,000 variable positions. (d) Raw density plot. (e) Singular value decomposition (SVD) plot after the removal of batch effects.

**Supplemental Figure 3**. Differential expression and gene-methylation correlation analyses. (a) “limma-voom” mean variance trend plot. (b) Source-of-variance analysis to build the differential expression confounder adjustment model. In the differential expression test, no significantly differentially expressed genes were found based on BH-adjusted *P*-values, so we checked the correlation between gene expression and DNA methylation levels for CpG sites from the promoter region (TSS200 and TSS1500) to find out if the different methylation signatures could be reflected on the gene level. (c) Scatter plot showing the correlation between methylation level and gene expression and the fold change of the corresponding hypermethylated CpG sites between normal and obese patients. CpG sites of interest are annotated with gene symbols. (d) Scatter plot showing the correlation between methylation level and gene expression and the fold change of the corresponding hypomethylated CpG sites between normal and obese patients. CpG sites of interest are annotated with gene symbols. Several genes are associated with cell apoptosis, adiposity, and metabolism with high negative Pearson correlations (PCC < −0.2) between bulk RNA-seq expression and methylation levels. Among these, SAFB2 stands out with a correlation of −0.306 and a significant fold change of 2.410, highlighting its critical roles in cell-cycle control, differentiation, stress response, and the modulation of genes essential for apoptosis and immune functions [114–118]. Furthermore, the functional enrichment of highly correlated genes with hypermethylated CpG sites reports multiple related pathways in Supplementary Table 3, including terpenoid backbone biosynthesis, endocrine resistance, and cell cycles. Genes related to cell proliferation and differentiation are also observed with high negative correlations to hypomethylated sites, including homeobox genes HOXA5 and HOXA9. These findings from the bulk RNA-seq analysis corroborate previous observations at the methylation level, suggesting that uHSCs from infants born to mothers with prepregnancy maternal obesity exhibit increased stemness due to alterations in cell-cycle regulation.

**Supplemental Figure 4**. Confounder adjustment quantile–quantile (Q–Q) plot. (a) Q–Q plot before confounder adjustment, only modeled with sample groups. (b) Q–Q plot after confounder adjustment. Genomic inflation scores before and after adjustment are reported as λ-values. The red line denotes the diagonal *y* = *x* line.

**Supplemental Figure 5**. Validation on stem cell homogeneity. UMAP plot of single-cell profiles of two Barnyard controls (B1 and D1), non-maternally-obese control (C1, green), and maternally obese case (A1, red).

**Supplemental Figure 6**. WGCNA modules. (a) Module-trait association heatmap using β-values adjusted for confounding effects. (b) Complete gene networks including non-major WGCNA modules.

**Supplemental Table 1**. Gestational age associated with differentially methylated regions. Gestational age associated with differentially methylated regions (DMRs) were reported from public pregnancy-related methylation datasets. Starting and ending positions of the DMRs are listed with *P*-values < 0.05.

**Supplemental Table 2**. List of CpG feature used in maternal obesity classification model for TCGA cancer prediction. CpG features that are used for maternal obesity classification model building, gene annotation, promotor regions, and related KEGG pathways are listed.

**Supplemental Table 3**. Pathway enrichment for genes with high negative DNAm and gene expression correlation. Pathway databases, pathway names, and adjusted *P*-values are reported in the table. Pathways with FDR-adjusted *P*-values <0.05 are statistically significant.

## Abbreviations

AA: amino Acid; BH: Benjamini–Hochberg; BMI: body mass index; C: acylcarnitines; DE: differential expression; DIABLO: Data Integration Analysis for Biomarker discovery using Latent cOmponents; DM: differential methylation; DMR: differentially methylated regions; DOHaD: Developmental Origins of Health and Disease; EWAS: epigenome-wide association studies; FC: fold change; FDR: false-positive results; KEGG: Kyoto Encyclopedia of Genes and Genomes; LG: logistic regression; MDS: multidimensional scaling; NHPI: Native Hawaiian and Pacific Islander; PANDA: Preferential Attachment-based common Neighbor Distribution derived Associations; PC aa: diacyl phosphatidylcholine; PC ae: acyl-alkylphosphatidylcholine; PCC: Pearson correlation coefficient; PPI: protein–protein interaction; RF: random forest; SOV: source of variance; SVD: singular value decomposition; SVA: surrogate variable analysis; TSS: transcription start site; TCGA: The Cancer Genome Atlas; uHSC: umbilical cord blood hematopoietic stem cell; UMAP: Uniform Manifold Approximation and Projection; VSN: variance stabilization normalization; WGCNA: Weighted Gene Co-expression Network Analysis
