# Supplementary material for: Multiomics analysis of umbilical cord hematopoietic stem cells from a multiethnic cohort of Hawaii reveals the intergenerational effect of maternal prepregnancy obesity and risks for cancers
Source: Gigascience. 2025 May 19;14:giaf039. doi: 10.1093/gigascience/giaf039 (PMC12087453; doi:10.1093/gigascience/giaf039)
Supplement: giaf039_GIGA-D-24-00597_original_submission [file giaf039_giga-d-24-00597_original_submission.pdf]

GigaScience

Multi-omics Analysis of Umbilical Cord Hematopoietic Stem Cells from a Multi-ethnic Cohort of Hawaii Reveals the Intergenerational Effect of Maternal Pre-Pregnancy Obesity and Risk Prediction for Cancers

--Manuscript Draft--

**Manuscript Number:**	GIGA-D-24-00597
**Full Title:**	Multi-omics Analysis of Umbilical Cord Hematopoietic Stem Cells from a Multi-ethnic Cohort of Hawaii Reveals the Intergenerational Effect of Maternal Pre-Pregnancy Obesity and Risk Prediction for Cancers
**Article Type:**	Research
**Funding Information:**	

U.S. National Library of Medicine (R01 LM012373)	Dr Lana Garmire
U.S. National Library of Medicine (R01 LM012907)	Dr Lana Garmire
Eunice Kennedy Shriver National Institute of Child Health and Human Development (R01 HD084633)	Dr Lana Garmire
National Cancer Institute (P30 CA071789)	Not applicable
National Institutes of Health (T32 GM141746)	MS Yuheng Du
National Cancer Institute (T32 CA140044)	MS Yuheng Du

Abstract:

Background

Maternal obesity is a health concern that may predispose newborns to a high risk of medical problems later in life. To understand the intergenerational effect of maternal obesity, we hypothesized that the maternal obesity effect is mediated by epigenetic changes in the CD34+/CD38-/Lin- hematopoietic stem cells (uHSCs) in the offspring. Towards this, we conducted a DNA methylation centric multi-omics study. We measured the DNA methylation and gene expression in the CD34+/CD38-/Lin- uHSCs and metabolomics of the cord blood, all from a multi-ethnic cohort (n=72) from Kapiolani Medical Center for Women and Children in Honolulu, Hawaii (collected between 2016 and 2018).

Results

Differential methylation (DM) analysis unveiled a global hypermethylation pattern in the maternal pre-pregnancy obese group (BH adjusted p<0.05), after adjusting for major clinical confounders. KEGG pathway enrichment, WGCNA, and PPI analyses revealed hypermethylated CpG sites were involved in critical biological processes, including cell cycle, protein synthesis, immune signaling, and lipid metabolism. . Utilizing Shannon entropy on uHSCs methylation, we discerned notably higher quiescence of uHSCs impacted by maternal obesity. Additionally, the integration of multi-omics data-including methylation, gene expression, and metabolomics-provided further evidence of dysfunctions in adipogenesis, erythropoietin production, cell differentiation, and DNA repair, aligning with the findings at the epigenetic level. Furthermore, we trained a random forest classifier using the CpG sites in the genes of the top pathways associated with maternal obesity, and applied it to predict cancer vs. adjacent normal labels from samples in 14 Cancer Genome Atlas (TCGA) cancer types. Five of 14 cancers showed balanced accuracy of 0.6 or higher: LUSC (0.87), PAAD (0.83), KIRC (0.71), KIRP (0.63) and BRCA (0.60).

Conclusions

This study revealed the significant correlation between pre-pregnancy maternal obesity and multi-omics level molecular changes in the uHSCs of offspring, particularly in DNA methylation. Moreover, these maternal obesity epigenetic markers in uHSCs may predispose offspring to higher risks in certain cancers.

Powered by Editorial Manager® and ProduXion Manager® from Aries Systems Corporation

Corresponding Author:	Lana Garmire<br><br>UNITED STATES
Corresponding Author Secondary Information:	
Corresponding Author's Institution:	
Corresponding Author's Secondary Institution:	
First Author:	Yuheng Du
First Author Secondary Information:	
Order of Authors:	Yuheng Du
	Paula A. Benny
	Yuchen Shao
	Ryan J. Schlueter
	Alexandra Gurary
	Annette Lum-Jones
	Cameron B Lassiter
	Fadhl M. AlAkwaa
	Maarit Tiirikainen
	Dena Towner
	W. Steven Ward
	Lana Garmire
Order of Authors Secondary Information:	

Additional Information:	
Question	Response
Are you submitting this manuscript to a special series or article collection?	No
**Experimental design and statistics**<br><br>Full details of the experimental design and statistical methods used should be given in the Methods section, as detailed in our Minimum Standards Reporting Checklist. Information essential to interpreting the data presented should be made available in the figure legends.<br><br>Have you included all the information requested in your manuscript?	Yes
**Resources**	Yes

A description of all resources used, including antibodies, cell lines, animals and software tools, with enough information to allow them to be uniquely identified, should be included in the Methods section. Authors are strongly encouraged to cite Research Resource Identifiers (RRIDs) for antibodies, model organisms and tools, where possible.<br><br>Have you included the information requested as detailed in our Minimum Standards Reporting Checklist?	
**Availability of data and materials**<br><br>All datasets and code on which the conclusions of the paper rely must be either included in your submission or deposited in publicly available repositories (where available and ethically appropriate), referencing such data using a unique identifier in the references and in the "Availability of Data and Materials" section of your manuscript.<br><br>Have you have met the above requirement as detailed in our Minimum Standards Reporting Checklist?	Yes
GigaScience has policies and guidelines in place for the use of generative AI-writing tools such as ChatGPT. If you have used such writing tools to assist with writing the manuscript this must be declared and cited in the text. Authors should not list AI-writing tools and other AI-assisted technologies as an author or co-author and should acknowledge that they are fully responsible for text generated or refined by AI-writing tools.<p><br><br>A summary of use (particularly in the introduction or among methods) needs to be included at the end of the paper, and the outputs should also be included as a supplementary file hosted in GigaDB or other open repositories. Please read our guidelines for more information. <p>	Yes

Powered by Editorial Manager® and ProduXion Manager® from Aries Systems Corporation

By submitting to GigaScience, you are aware of the journal's AI-writing tools policy, and if you have declared use of such tools below, you have acknowledged this where appropriate in your manuscript and have made a summary of use and outputs available. <p> <b>AI-assisted writing tools have been used in the preparation of this manuscript?	

Powered by Editorial Manager® and ProduXion Manager® from Aries Systems Corporation

Manuscript

Click here to access/download;Manuscript;GigaScience_revision_manuscript_

Multi-omics Analysis of Umbilical Cord Hematopoietic Stem Cells from a Multi-ethnic Cohort of Hawaii Reveals the Intergenerational Effect of Maternal Pre-Pregnancy Obesity and Risk Prediction for Cancers

Yuheng Du[1], Paula A. Benny[2], Yuchen Shao[3], Ryan J. Schlueter[2], Alexandra Gurary[2], Annette Lum-Jones[4], Cameron B Lassiter[4], Fadhl M. AlAkwaa[5], Maarit Tiirikainen[4], Dena Towner[2], W. Steven Ward[2], Lana X Garmire[1*]

1. Department of Computational Medicine and Bioinformatics, University of Michigan, Ann Arbor, MI

2. Department of Obstetrics and Gynecology, University of Hawaii, Honolulu, HI

3. Department of Electrical Engineering and Computer Science, University of Michigan, Ann Arbor, MI

4. University of Hawaii Cancer Center, Population Sciences of the Pacific Program-Epidemiology, Honolulu, HI

5. Department of Neurology, University of Michigan, Ann Arbor, MI

*. Corresponding author email: lgarmire@med.umich.edu

Keywords: obesity, Native Hawaiian, Hematopoietic stem cells, Multi-omics, cord blood, methylation, pregnancy

Abbreviation:

AA: Amino Acid

BH: Benjamini-Hochberg

BMI: Body mass index

C: Acylcarnitines

DE: Differential expression

DIABLO: Data Integration Analysis for Biomarker discovery using Latent cOmponents

DM: Differential methylation

DMR: Differentially methylated regions

DOHaD: Developmental Origins of Health and Disease

EWAS: Epigenome-wide association studies

FC: Fold Change

FDR: False positive results

KEGG: Kyoto Encyclopedia of Genes and Genomes

LOG: Logistic regression

MDS: Multi-dimensional Scaling

NHPI: Native Hawaiian and Pacific Islander

PANDA: Preferential Attachment-based common Neighbor Distribution derived Associations

PC aa: Diacyl phosphatidylcholines

PC ae: Acyl-alkylphosphatidylcholines

PCC: Pearson correlation coefficients

PPI: Protein-Protein Interaction

RF: Random Forest

SOV: Source of variance

SVD: Singular value decomposition

SVA: Surrogate variable analysis

TSS: Transcription start site

TCGA: The Cancer Genome Atlas

uHSCs: Umbilical cord blood hematopoietic stem cells

UMAP: Uniform Manifold Approximation and Projection

VSN: Variance Stabilization Normalization

WGCNA: Weighted Gene Co-expression Network Analysis

Abstract

Background: Maternal obesity is a health concern that may predispose newborns to a high risk of medical problems later in life. To understand the intergenerational effect of maternal obesity, we hypothesized that the maternal obesity effect is mediated by epigenetic changes in the CD34+/CD38-/Lin- hematopoietic stem cells (uHSCs) in the offspring. Towards this, we conducted a DNA methylation centric multi-omics study. We measured the DNA methylation and gene expression in the CD34+/CD38-/Lin- uHSCs and metabolomics of the cord blood, all from a multi-ethnic cohort (n=72) from Kapiolani Medical Center for Women and Children in Honolulu, Hawaii (collected between 2016 and 2018).

Results: Differential methylation (DM) analysis unveiled a global hypermethylation pattern in the maternal pre-pregnancy obese group (BH adjusted $p<0.05$), after adjusting for major clinical confounders. KEGG pathway enrichment, WGCNA, and PPI analyses revealed hypermethylated CpG sites were involved in critical biological processes, including cell cycle, protein synthesis, immune signaling, and lipid metabolism. . Utilizing Shannon entropy on uHSCs methylation, we discerned notably higher quiescence of uHSCs impacted by maternal obesity. Additionally, the integration of multi-omics data-including methylation, gene expression, and metabolomics-provided further evidence of dysfunctions in adipogenesis, erythropoietin production, cell differentiation, and DNA repair, aligning with the findings at the epigenetic level. Furthermore, we trained a random forest classifier using the CpG sites in the genes of the top pathways associated with maternal obesity, and applied it to predict cancer vs. adjacent normal labels from samples in 14 Cancer Genome Atlas (TCGA) cancer types. Five of 14 cancers showed balanced accuracy of 0.6 or higher: LUSC (0.87), PAAD (0.83), KIRC (0.71), KIRP (0.63) and BRCA (0.60).

Conclusions: This study revealed the significant correlation between pre-pregnancy maternal obesity and multi-omics level molecular changes in the uHSCs of offspring, particularly in DNA methylation. Moreover, these maternal obesity epigenetic markers in uHSCs may predispose offspring to higher risks in certain cancers.

Introduction

Maternal obesity has emerged as a primary health concern during pregnancy, with its prevalence alarmingly increasing. According to a study by the Centers for Disease Control and Prevention, the percentage of women experiencing pre-pregnancy obesity in the United States escalated from 26% to 29% between 2016 and 2019 [1]. Born to mothers with obesity, higher birth weight is associated with a higher incidence of childhood cancers such as leukemia and neuroblastoma [2,3], as well as greater risks of prostate and testicular cancers in men [4–6] and breast cancer in women [7]. Moreover, maternal obesity may have a intergenerational effect and set the stage for increased chronic disease susceptibility later in the adulthood of offspring [8,9]. The hypothesis of the utero origin of diseases proposes that numerous chronic diseases have their origins in the fetal stage, the earliest phase of human development [10,11]. Some researchers have speculated higher stem cell burdens in newborn babies born from obese mothers [12]. Altered hormonal environment and nutrient availability can induce critical changes in fetal stem cells [13], which may predispose these cells to malignant transformation, aligning with the idea of the cancer stem cell hypothesis that cancer cells have stem cell-like properties with an uncontrolled self-renewal program [14–16]. In particular, a study showed increases in cord blood CD34+CD38- stem cell and CD34+ progenitor cell concentrations with maternal obesity [17], suggesting that the higher proportions of stem cells in cord blood may make the babies more susceptible to obesity and cancer risks.

However, so far little work provides direct molecular links as to how maternal obesity affects cellular function and increases the disease risk in offspring.

To seek answers in this area, we conducted an epigenome-centered multi-omics study to directly pinpoint the effect of maternal obesity in umbilical cord blood hematopoietic stem cells (uHSCs). Epigenetics is chosen as the center of multi-omics integration, as it is both inheritable and susceptible to modification by diseases. Thus, it may serve as a plausible mediator in the transmission of the effects of maternal obesity to offspring. We incorporate gene expression for cord blood stem cells and metabolomics data from the cord blood serum as the downstream readout of epigenetics changes. By elucidating these molecular connections, we provide a systematic understanding of how maternal obesity during pregnancy can influence the multiple types of molecular profiles of newborns. Such knowledge may ultimately help develop early therapeutic interventions at the molecular level to mitigate these intergenerational health risks due to maternal obesity.

Methods

Overview of the maternal pre-pregnancy cohort with baby cord blood

In this study, baby cord blood samples from 72 pregnant women (34 obese; 38 non-obese) who delivered at Kapiolani Medical Center for Women and Children in Honolulu, Hawaii (2015-2018) were collected. The study was approved by the Western IRB (WIRB Protocol #20151223). Patients meeting the inclusion criteria were identified from pre-admission medical records with pre-pregnancy BMI $\geq$ 30.0 (maternal obesity) or 18.5-25.0 (normal weight).

Pregnant women undergoing elected C-sections at ≥37 weeks gestation were included, to minimize confounding events during the labor. Patient exclusion criteria included pregnant women with preterm rupture of membranes, labor, multiple gestations, pregestational diabetes, hypertensive disorders, cigarette smokers, infection of human immunodeficiency virus or hepatitis B virus, and chronic drug use. Demographic and phenotypic information was recorded, including maternal and paternal age, ethnicity, gestational weight gain, gestational age, parity, and gravidity. For newborns, Apgar scores were documented at 1 minute and 5 minutes post-delivery. The Apgar score serves as a comprehensive assessment of a newborn's health, with a normal range considered to be between 7 to 10 [18].

Sample preparation and methylation profiling

The baby cord blood sample was collected in the operating room under sterile conditions at the time of the C-section (Pall Medical Cord Blood Collection Kit containing 25ml citrate phosphate dextrose). The umbilical cord was first cleansed with chlorhexidine swabs before cord blood collection. The total volume of collected blood was measured and recorded before aliquoting to conical tubes for centrifugation. The tubes were centrifuged at 200g for 10 min, and plasma was collected. The plasma volume was replaced with 2% FBS/PBS. Negative selection reagents were added to the blood and incubated for 20 min (Miltenyi Biotec, Auburn, CA). The cord blood was diluted with an equal volume of 2% FBS/PBS. A 20ml aliquot of the diluted blood was layered over a density gradient (15ml Lymphoprep) and centrifuged at 1200g for 20 min. The top layer containing an enriched population of stem cells was collected, centrifuged at 300g for 8 min, and then washed in 2% FBS/PBS. Red blood cells were lysed using ammonium chloride (9:1) with incubation on ice for 10 min, washed twice, and then resuspended in 100µl of 2% FBS/PBS.

Cells were stained with Lineage FITC and CD34 APC for 45 min on ice, washed twice, and then sorted using the BD FacsAria III. Hematopoietic stem cells (CD34+/CD38-/Lin-) were collected and stored at -80°C until DNA/RNA extraction.

DNA and RNA were extracted simultaneously using the AllPrep DNA/RNA extraction kit (Qiagen). DNA purity and concentration were quantified in Nanodrop 2000 and Picogreen assay. Bisulfite conversion of 500 ng DNA was performed using the EZ DNA Methylation kit (Zymo), followed by sample processing for Infinium HumanMethylation450 bead chips (Illumina) according to the manufacturer's instructions. Bead chips were analyzed at the Genomics Shared Resource at the University of Hawaii Cancer Center.

Bulk RNA sequencing

A total of 50 RNA samples were prepared for bulk RNA Sequencing. RNA concentration and RIN score were assayed using Nanodrop 2000 and Agilent Bioanalyzer. A total of 200 ng of high-quality RNA (RIN≥7) was subjected to library construction (polyA) and sequenced on HS4000 (2x100) at the Yale Center for Genome Analysis, Connecticut to obtain at least 25M paired reads per sample.

Methylation data pre-processing

The overall preprocessing workflow is shown in **Supplemental Figure 1**. R version 3.6.3 was used for all analyses. As the first step of quality assessment, sex chromosome methylation patterns were analyzed to check for potential sex mismatches between reported and inferred sex using the getSex() function in minfi[19]. No samples with discrepancies between reported and inferred sex were identified or flagged for exclusion **Supplemental Figure 2A**. Raw intensity

data (.idat) were extracted using the 'ChAMP' package (version 2.16.2) in R with *champ.load()* function [19–22]. For the filtration step, background controls were subtracted from the data, and raw data that did not pass detection P-value of 0.05 were removed. CpG sites whose probes had known underlying SNPs and association with XY chromosomes were removed from analysis due to potential confounding. The quality controls included checking the raw density distribution, multi-dimensional scaling (MDS) plot, and median intensity values of methylated and unmethylated probes to identify potential outliers or poorly performing samples (**Supplemental Figure 2B-D**). After BMIQ normalization using *champ.norm()* function [23], the batch effect (including slide and array) due to non-biological technical variation caused by experiment handling was removed using the ComBat function in the ChAMP package, confirmed by the singular value decomposition (SVD) plot (**Supplemental Figure 2E**). A total of 1,992 cross-hybridizing probes were removed using the probe list from ExperimentHub (query id 'EH3129') as reported in Chen et al. 2013 [24]. For each CpG site, the methylation score was initially calculated as the beta value, a fluorescence intensity ratio between 0 and 1. To reduce the heteroskedasticity for downstream statistical analysis, the M-values were transformed from beta-values using lumi (ver 3.1.4) in R [25–28]. A total of 408,773 CpG sites remained for downstream analysis after probe filtering, quality control, normalization, batch correction, and cross-hybridizing probes removal.

Source of variation analysis and confounding adjustment

To eliminate potential confounding factors of pre-pregnant maternal obesity among the 13 clinical factors, we conducted a source of variation analysis with a collection of ANOVA tests to identify the clinical factors that significantly contribute to the methylation level variation, as

done before [29,30]. The variables with F statistics greater than 1 (the error value) were determined as confounders and subjected to confounding adjustment. These factors include the baby's sex, net weight gain during the pregnancy, maternal age, maternal ethnicity, paternal ethnicity, gravidity, and gestational age. To adjust for confounding effects, a multivariate regression model is built using the 'limma' package to fit methylation M values of each CpG site, using the confounding factors above. The remaining residuals on the M values were considered to be confounding adjusted for the subsequent bioinformatics analysis of DNA methylation. To assess the bias and inflation in the differential methylation findings, we used Bayesian method "bacon" to calculate the genomic inflation (lambda) values before and after the confounder adjustment [31]. Additional surrogate variable analysis (sva) and randomly shuffled null lambda calculation were performed to determine the need for inflation adjustment [32]. The null model lambda was 0.96. No surrogate variables were identified for correction in the adjusted model. Thus the observed inflation reflects the true biological signal rather than systematic bias, and no further inflation correction was performed on this confounder adjusted model.

Bioinformatics analysis of differential methylation (DM)

A moderated t-test from the 'limma' R package (version 3.42.2) [33] was used for detecting DM CpG sites between healthy controls and cases with M values. The p-values were adjusted for multiple hypotheses testing using Benjamini-Hochberg FDR. CpG sites with FDR <0.05 were considered statistically significant. To minimize the effect of the gestational age, CpG sites located within the gestational-age-related differentially methylated regions (DMR) were removed. A total of 130 DMRs related to gestational age were identified using linear regression analysis performed with bumphunter [34] across eight public datasets including a total of 248

patients.: GSE31781 [35], GSE36829 [36], GSE59274 [35,37], GSE44667 [38], GSE74738 [39], GSE49343 [40], GSE69502 [41], and GSE98224 [42,43]. The complete list of DMRs was included in **Supplemental Table 1**. Hypermethylation and hypomethylation states were defined by the values of log2 Fold Change (log_2FC) of M values in cases compared to controls: hypermethylation if bigger than 0, and hypomethylation if less than 0. Corresponding genes and feature locations of these differential CpG sites were annotated using IlluminaHumanMethylation450kanno.ilmn12.hg19 (ver 0.6.0) [44] .

KEGG pathway enrichment analysis

'gometh' function from R package "missMethyl" (version 1.26.1) [45–48] was used for KEGG pathway enrichment [49–51] with DNA methylation data. DM sites were used for pathway enrichment within five supergroups from KEGG pathways: Metabolism, Genetic Information Processing, Environmental Information Processing, Cellular Processes, and Organismal Systems. Pathways with adjusted p-values less than 0.05 were considered significant. Pathway scores for protein pathways (KEGG: Transcription, Translation, Folding, sorting and degradation) and immune pathways (KEGG: Immune system) were calculated with averaged beta values from the promoter region CpG sites. To validate the enrichment of significant CpGs in specific pathways, we used the hypergeometric test, which calculates the probability of observing k or more significant CpGs in a pathway by chance, given the total CpGs on the Illumina array (N), the total CpGs in the pathway (K), and the total significant CpGs identified in our study (n). The formula is: $P(X \geq k) = 1 - \sum_{i=0}^{k-1} \frac{\binom{K}{i}\binom{N-K}{n-i}}{\binom{N}{n}}$ where $\frac{a}{b}$ represents the binomial coefficient.

Weighted co-expression network analysis

Firstly, we adjusted all beta values with clinical confounders, then summarized the DM CpG sites at the gene level by averaging the beta values in the promoter regions (those in the TSS200 and TSS1500 promoter regions). Next, we transformed adjusted beta values to adjusted M values for the downstream adjacency matrix construction. We used adjusted M values for the weighted gene co-expression network analysis (WGCNA) with R package 'WGCNA' (version 1.70-3) [52,53]. The soft threshold for the weighted adjacency matrix with an adjusted $R^2>0.8$ was 7. The topological overlap matrix was constructed for hierarchical clustering. Modules were identified by the dynamic tree-cut algorithm. The networks were exported to Cytoscape with an edge weight greater than 0.03 in each module. The genes with the highest betweenness and degree in the WGCNA network were identified as the hub genes for different modules.

Protein-protein interaction network analysis

For the protein-protein interaction (PPI) network analysis, DM genes are used as the inputs and were mapped on the STRING database (version 10) [54]. Significantly functionally associated protein pairs were identified using PANDA (Preferential Attachment based common Neighbor Distribution derived Associations) (version 0.9.9) [55]. KEGG pathways associated with these protein pairs (in terms of genes) were found using PANDA. The bipartite network graph was visualized using Cytoscape (version 3.8.1) [56].

Stemness score computation

The stemness score was based on Shannon entropy and scaled plasticity, as proposed previously [57]. Shannon entropy has been widely applied in developmental biology, particularly in stem cell research [58–60]. The formulas are shown below:

$$Entropy = \sum_{i=1}^{N} \frac{-\frac{CpG_i}{\sum_{i=1}^{N} CpG_i} log\left(\frac{CpG_i}{\sum_{i=1}^{N} CpG_i}\right)}{log(N)}$$

$$StemnessScore = \frac{Entropy - min(Entropy)}{max(Entropy) - min(Entropy)}$$

N is the total number of CpG sites. CpG is represented by the beta value on each CpG probe. The stemness score was calculated for all samples using all remaining 408,773 CpG sites after the preprocessing. A Wilcoxon rank test was performed between the stemness scores of the healthy and maternally obese groups.

Bulk RNA-seq data processing

The Illumina universal adapter regions of raw RNA-seq data were first trimmed using BBMap (version 38.91) [61]. All raw sequences passed the quality control using fastqc (version 0.11.8) [62]. The trimmed .fastq files were aligned by STAR (version 2.7.0f) [63] to the human Ensembl genome (Homo_sapiens.GRCh38.dna.primary_assembly.fa) and Ensembl annotation (Homo_sapiens.GRCh38.94.gtf). The gene expression counts were calculated using featureCount [64] from Subread (ver 1.6.4) [65].

Differential expression (DE) of RNA-Seq data

The limma voom transformation was used on RNA-seq data to model the mean-variance relationship of the log2 counts [66], supporting the empirical Bayes analysis pipeline in limma

(**Supplemental Figure 3A**). Source of variance analysis was performed to find the clinical confounders with ANOVA tests. The significant confounders included: Maternal_Age, baby sex, hemoglobin, sample group, net weight gain, maternal ethnicity, gravidity and parity (**Supplemental Figure 3B**). The statistically significant DE genes between healthy controls and maternally obese cases were found with confounder adjustment using the 'DESeq2' (version 1.26.0) [67] and 'limma-voom' function from 'limma' package [33]. The p-values were adjusted for multiple hypotheses testing using BH adjustment. No significant differential genes were found with adjusted p-values less than 0.05.

Correlation analysis between bulk RNA-seq and methylation data

A subset of 47 patients have done both methylation and RNA-seq assays. Pearson correlation coefficients (PCC) were calculated between gene expression and methylation beta values from the promoter regions, among the same patients. As mostly a negative correlation between gene expression and DNA methylation in the promoter region is expected, genes with a high negative correlation (PCC<-0.2) were used for pathway enrichment using TOPPFUN [68–70]. Top genes of interest were selected with the absolute value Fold Change>1.5 in gene expression and gene-methyl correlation <-0.3 for hyper- and hypo-methylated CpG sites.

Metabolomics analysis

Metabolomics data were acquired from a previously published study involving 87 patients in the same cohort from three batches (metabolomics workbench study ID ST001114) [71]. Targeted metabolites were generated with LC-MS, and untargeted metabolites were generated with GC-MS. After the removal of compounds missing in more than 10% of samples, a total of 185

metabolites remained, including 10 amino acids (AA), 40 acylcarnitines (C), 35 acyl/acyl phosphatidylcholines (PC aa), 38 acyl/alkyl phosphatidylcholines (PC ae) and 62 untargeted metabolites. The raw metabolite data were log-transformed, standardized, normalized using variance stabilization normalization (VSN), and batch corrected with ComBat function in sva pacakge[72]. Differential metabolites were identified by limma, with clinical confounders adjustment.

Multi-omics integration on metabolomics, epigenomics, and transcriptomics

A subset of 42 patients have the matched methylation, gene expression, and metabolomics data. We applied multi-omics integration with Data Integration Analysis for Biomarker discovery using Latent cOmponents (DIABLO) implemented in the mixOmics package [73]. DIABLO finds the correlated consensus latent variables among different omics in the supervised manner. Top DIABLO features for each omic were selected based on the loading values. We integrated the pathway-level methylation, gene, and metabolite interaction using pathview [74].

Evaluation of maternal pre-pregnancy obesity biomarkers in cancer prediction

We collected Infinium HumanMethylation450 data for a total of 14 cancer datasets (adjacent normal samples > 10): BLCA, BRCA, COAD, ESCA, HNSC, KIRC, KIRP, LIHC, LUAD, LUSC, PAAD, PRAD, THCA, UCEC from The Cancer Genome Atlas (TCGA data portal: https://portal.gdc.cancer.gov/). In total, 6428 samples were obtained, consisting of 5715 tumor samples and 713 adjacent normal tissues.

To build the obesity classification model with maternal obesity biomarkers, we selected 61 hypermethylated CpG sites from the promoter regions of the genes involved in the top five

significant pathways based on the missMethyl KEGG enrichment results. This includes cell cycle, ribosome, nucleocytoplasmic transport, ribosome biogenesis in eukaryotes, and mTOR signaling pathway. We split the 72 maternal obesity and control samples at a ratio of 80/20 with 5-fold cross-validation, then constructed a series of classification methods using the *lilikoi* R package, where Random Forest (RF) was the winning model [75,76]. Next, we applied this RF obesity model on 14 TCGA dataset to perform cancer/normal sample prediction. We report accuracy, balanced accuracy, and F1 score for model performance as done before [75].

Results

Overview of study design and cohort characteristics

This study aims to investigate the intergenerational effect of pre-pregnancy maternal obesity on offspring. A total of 72 patients who elected to deliver full-term babies through C-sections were recruited from Kapiolani Medical Center for Women and Children in Honolulu, Hawaii from 2016 to 2018. This cohort reflects the multi-ethnic population character of Hawaii, including Asian (N=29), Caucasian (N=15), and Native Hawaiian and Pacific Islanders or NHPIs (N=28). Among them, 38 deliveries are in the healthy control group and 34 are cases with pre-pregnancy maternal obesity. We excluded natural virginal births, to avoid its potential confounding effect on multi-omics profiles. We also carried out stringent recruitment selection criteria, including matching the mothers' ages as much as possible, as well as similar net gestational weight gain to minimize its confounding effect over maternal pre-pregnancy maternal obesity. The overall study design is shown in **Figure 1**. Briefly, upon collecting the blood samples, umbilical cord blood hematopoietic stem cells (uHSCs) were enriched by FACS sorting with CD34+CD35-

LIN- markers (see **Methods**). We extracted DNA and RNA from these uHSCs for Illumina 450k array based DNA methylation and bulk RNA-Seq sequencing respectively. The plasma from these cord blood samples was subject to untargeted metabolomics assays using GC-MS and targeted metabolomics assays using LC–MS [71]. Given the rationale that DNA methylation could be the mediator for exerting the intergenerational effect of maternal obesity [77,78], we carried out multi-omics data integration analysis in the DNA methylation-centric manner.

The demographic details and clinical information of these patients are summarized in **Table 1**. The distributions of the most representative variables are shown in **Figure 2**. Among categorical demographic variables, the distribution of baby sex had no statistical difference between obese and health groups, whereas the ethnicity distributions among mothers and fathers, parity and gravidity are statistically different (P<0.05) between the two groups (**Figure 2A-2E**). Besides maternal pre-pregnancy BMI, other maternal parameters such as maternal age, gestational week, net weight gain and hemoglobin are also not statistically significantly different between the two groups per study design (**Figure 2F-2I, Table 1**). While mothers of Asian ethnicity are the majority in the control group, NHPIs account for the majority of the maternal-obese group, revealing the health disparity issue known in the state of Hawaii [79]. Moreover, the control group has lower parities and gravidities, compared to the cases. Babies born to obese mothers show significantly higher (P<0.05) body weights compared to the control group, as expected [80]. Other parameters including the baby gender, head circumference, body length, and APGAR score at 5 min after birth are not statistically significantly different between case and control groups (**Figure 2J-2M**).

Global hypermethylation pattern revealed by CpG level methylation analysis

Quality control of methylation data showed no significant sample outliers and no remaining batch effect after ComBAT correction (**Supplemental Figure 2D**). For scientific rigor, it is critical to adjust for confounding in DNA methylation association analysis [81]. Thus we performed the source of variance (SOV) analysis on the beta values of the DNA methylation with respect to physiological and phenotypic information, in order to assess potential confounding factors systematically [29,30,81]. As shown in **Figure 3A**, marginal F-statistics in the SOV analysis show that the dominating contribution to DNA methylation variation is maternal pre-pregnancy obesity status, confirming the quality of the study design which aimed to minimize other confounders' effect. The other minor confounding factors include baby sex, maternal age, maternal ethnicity, net weight gain during pregnancy, paternal ethnicity, gravidity, and gestational age (F-statistics>1). After adjusting these factors by linear regression, all have reduced F-statistics of less than 0.5 (**Figure 3B**) except maternal pre-pregnancy obesity, confirming the success of confounding removal. The quantile-quantile (QQ) plot and genomic inflation factor were used to assess the confounder adjusting model (**Supplemental Figure 4**). A decrease in genomic inflation factor (lambda) was observed with adjustment of confounders. Although the adjusted model had lambda of 1.28, no surrogate variables were identified for correction in the adjusted model. Therefore the observed inflation reflects mostly the true biological signal, and no further inflation correction was performed.

Next, we conducted differential methylation (DE) analysis on the confounding adjusted DNA methylation data (**Methods**). We observed a global hypermethylation pattern in pre-pregnancy obese samples, with 10,211 hypermethylated vs. 5,362 hypomethylated CpG sites (**Figure 3C**).

The top 20 differentially hypermethylated and hypomethylated CpG sites are reported in **Table 2**, respectively. These CpG sites are related to a wide variety of biological functions, including inflammation (CD69, ADAM12), transcription factors (ZNF222, HMGN4, LHX6, TAF3), proliferation and apoptosis (HDAC4, DHRS4, LRCH3, SAFB2, CRADD, EBF3, PRKAR1B). Some top DM CpG sites are directly associated with obesity, including HDAC4 [82] and PLEC1[83].

We further examined the distributions of these differentially methylated sites, relative to the CpG island regions and promoter proximity (**Figure 3D-E**). A big fraction (42.2%) of the DM sites are located in CpG islands [84,85], significantly higher than that from the Illumina 450K annotation (P<2E-16). CpG islands are more frequent in the hypermethylated sites (43.8%) than in the hypomethylated sites (39.1%), which is consistent with the global hypermethylation pattern. Relative to gene localization, DM sites are most frequent (39.3%) in the promoter regions (including 18.7% and 20.6% in TSS200 and TSS1500 respectively) as expected.

Functional analyses reveal the association between maternal obesity and cell cycle, immune function and metabolic changes in the cord blood of offspring

To investigate the biological functions related to the epigenome alternation, we conducted systematic analysis of DM sites employing multiple methods: KEGG pathway enrichment analysis, Weighted Gene Co-expression Network Analysis (WGCNA), and Protein-Protein Interaction (PPI) network analysis.

KEGG pathway enrichment analysis on hypermethylated CpG sites identified five significant pathways with hypergeometric FDR<0.05 (**Figure 4A)**, including the cell cycle, ribosome,

nucleocytoplasmic transport, ribosome biogenesis in eukaryotes, and mTOR signaling pathway. Cell cycle, ribosome, and nucleocytoplasmic transport pathways are essential to normal cell functioning. mTOR signaling pathway coordinates the nutrient-mediated metabolism, immune responses and cell cycle progression, and dysregulation of mTOR could lead to various diseases such as cancer and obesity [86]. There was no significantly enriched pathway emerging from hypomethylated CpG sites. The maternally obese group shows significantly higher methylation levels in KEGG protein synthesis and immune system pathway collections compared to the control group, indicating repression in immune response as well as translation and protein synthesis (**Figure 4B-C**). Similarly, we further explored the differential potential, or stemness, of umbilical cord Hematopoietic Stem Cells (uHSCs). We first confirmed the homogeneity of uHSCs by single-cell RNA sequencing UMAP plot (**Supplemental Figure 5**). We calculated the cell stemness scores using the DNA methylation beta values similar to others [87]. uHSCs derived from the maternally obese group exhibit significantly elevated stemness scores ($P<0.01$) in comparison to the control group **(Figure 4D)**, confirming the results in KEGG pathway analysis.

Next, we applied WGCNA to cluster co-regulation of gene-level methylation, by averaging CpG sites to affiliated genes (see **Methods**). Five co-expression modules are identified, using the M-values adjusted for clinical confounders (**Supplemental Figure 6A)**, and all modules show positive correlations with maternal obesity except one. The largest turquoise module (**Figure 4E**) is related to cell cycle, protein synthesis, and transport and vesicle trafficking pathways through pathway enrichment analysis. Some hub genes in this module are identified, including INTU, ANAPC7, and AGBL5. These genes were reported essential for maintaining cell polarity (INTU)[88], proliferation (ANAPC7) [89] and glycemic control (AGBL5) [90]. The brown module

(**Figure 4F**) is enriched with immune response pathways, in which TLR6 is identified as a hub gene. The other yellow module is related to ion homeostasis, and the gray module is related to the p53 pathway, apoptosis, cell senescence, and ER stress (**Supplemental Figure 6B)**. The only negatively correlated blue module is associated with axon guidance and VEGF signaling pathway (**Supplemental Figure 6B**).

Furthermore, we examined the PPI network, using the gene-level DNA methylation as surrogates (**Figure 4G**). The PPI analysis identifies 14 unique pathways (FDR < 0.05) predominantly associated with hypermethylated CpG sites in the TSS200 and TSS1500 regions. The top five largest pathways included ribosome, proteasome, cell cycle, axon guidance, RNA polymerase, and neuroactive ligand-receptor interaction. Taken all three types of systematic analyses together, cell cycle, immune function and protein synthesis are ubiquitously highlighted, suggesting that these biological functions in cord blood stem cells are negatively impacted by maternal obesity.

Multi-omics analysis reveals disruptions in cell cycle and metabolic pathways

To systematically investigate the epigenetic, transcriptomic, and metabolomic alterations induced by maternal obesity, we performed multi-omics integration analysis on this cohort. We employed DIABLO, a supervised integration method that extracts features associated with maternal obesity, based on the correlations in the embedding space [73]. **Figure 5A-C** shows that methylation data provide the clearest separation between obese and control groups, confirming the value of the earlier DNA methylation-centered analysis.

The top 25 features from each omic with the highest feature weights (loadings) following integrated canonical correlation analysis are demonstrated in **Figure 5D-F**. The methylation features with the highest weights related to maternal obesity include CpG sites involved in cell-cycle control, glucose metabolism, and adipogenesis (FOXO1 [91]), DNA repair (LIG3, SMUG1), erythropoietin pathway and differentiation (EPO, CSNK2A1, CSF1), which are hypermethylated in the obese group. Hypomethylation of LEP (encoding leptin) was also observed as a top feature, aligning with prior findings that maternal obesity is associated with elevated maternal leptin levels, a known marker of adipose tissue [92]. These featured CpG sites indicate repression in fat metabolism and DNA repair and reduced differentiation potential. In the transcriptomic space, many genes related to mRNA splicing (SRRM1, IGF2BP1, IGF2BP2, CNOT4) have increased expression levels due to maternal obesity. Among the metabolite features, essential sugars (glucose, xylose), poly-unsaturated fatty acids (oleic acids, DHA, arachidonic acid), and phosphatidylcholine (PCs) are mostly decreased in the obese group; whereas most acylcarnitines (C) are elevated. The metabolic changes show an overall accumulation of saturated fatty acid, but repression of fat breakdown, glucose, and unsaturated fatty acid generation. As poly-unsaturated fatty acids (eg. arachidonic acid) have important anti-inflammatory effects, the results indicate a pro-inflammatory environment in offspring born of pre-pregnant obese mothers.

The maternal obesity classification model is predictive of KIRC, LUSC, and PAAD cancers in TCGA

Maternal pre-pregnancy obesity may predispose a higher risk of cancer and other diseases in babies' later life, via epigenetic modification [12,17]. To check this assumption, we built maternal

obesity random forest classification models using a total of 63 hypermethylated promoter region marker CpG sites obtained from top KEGG pathways which overlapped with the 14 TCGA cancer data that had sufficient numbers (n>10) of adjacent normal samples (**Supplemental Table 2**). The maternal obesity random forest model resulted in balanced accuracy of 0.93 on the obesity training data. We then applied this obesity classification model to predict the known adjacent normal and tumor tissue labels from DNA methylation data of 14 TCGA cancers, each of which has sufficient (n>10) tumor adjacent normal samples (**Figure 6**). This allows us to assess if the maternal obesity DNA methylation markers are associated with cancers. As shown in **Figure 6A**, three cancer types have good prediction balanced accuracy (Bal acc)of at least 0.7: LUSC (0.87), PAAD (0.83), KIRC (0.71), and two additional cancers reached 0.6: BRCA (0.60) and KIRP (0.63). The other metrics, including overall accuracy and F-1 scores are shown in **Figure 6B**. Thus, these results show that CpGs epigenetic markers associated with maternal obesity are also potentially associated with tumorigenesis in lung, breast, pancreas and kidney. Our result preliminarily supports that maternal pre-pregnancy obesity may predispose offspring to increased risks in certain cancers later in life through epigenetic modifications.

Discussion

Maternal obesity is one of the most urgent health concerns worldwide. Pre-pregnancy maternal obesity could cause various pregnancy-related complications and predispose offspring to cardiometabolic complications and chronic diseases in the long term [9]. Multiple cross-continental large cohort meta-analyses have shown that maternal obesity is directly associated with offspring's risk of obesity, coronary heart disease, insulin resistance, and adverse

neurodevelopmental outcomes based on longitudinal observational studies [9,93,94]. To directly pinpoint the molecular level changes in offspring by maternal pre-pregnancy obesity, we used cord blood stem cells as the studying material, which serve as a great surrogate revealing the newborn's metabolism and immune system changes at the time of birth [95]. The current study expands on previous effects and investigates the direct impact of maternal obesity on uHSCs programming, the progenitor of the immune cell population, using a multi-omics (epigenetic, gene expression, and metabolite) analysis approach from a unique multi-ethnic cohort.

Centered around methylation changes, three complimentary functional analysis approaches (KEGG, WGCNA, and PPI) consistently demonstrated that maternal obesity impacts multiple biological functions including hypermethylation in promoters of genes involved in cell cycle, ribosome biogenesis, and mTOR signaling pathways. Moreover, mTOR signaling pathway also plays a crucial role in metabolism and cell cycle regulation, disruption in this pathway leads to insulin resistance and long-term diseases [96]. We observed a significant increase in stemness scores among uHSCs affected by maternal obesity, aligning with expected downregulation in the cell cycle gene expression due to observed hypermethylation in the promoters of these genes. Higher stemness scores indicate enhanced quiescence, shifting the balance between stem cell maintenance and differentiation towards the former. Unlike adult HSCs, fetal/neonatal HSCs typically exhibit higher proliferation and self-renewal capabilities, crucial for blood cell regeneration and innate immune system development [97]. Our findings provide strong epigenetic evidence that maternal obesity compromises the maturation processes in neonatal uHSCs, which may predispose newborns to immunological disorders.

The subsequent multi-omics integration analysis expanded conclusions from methylation analysis to additional metabolomics readouts that are also linked to biological functions eg. cell cycle and inflammatory pathway. We thus propose the conceptual model to illustrate the effect of maternal pre-pregnancy obesity (**Figure 7**). Maternal obesity leads to nutrient deficiency with lower levels of essential amino acids and fatty acids in the newborn blood and disrupts the lipid metabolism homeosis in offspring. These metabolite changes further induce cell membrane instability and repress cell cycle progression, cell proliferation[98], enhancing the dysregulation of these functions preexisting at the methylation level. Lipid dysregulation may also enhance the pro-inflammatory environment, which in turn induces complications in offspring later in life, such as cardiovascular diseases. Such a proposed model is also consistent with and further strengthens previous studies at the metabolomics or epigenome levels. For example, previous metabolomics studies of cord blood showed metabolic derangement predisposes newborns to cardiometabolic and endocrine diseases, and disrupt the normal hormone function and neonatal adiposity [92,99]. Previous epigenome-wide association study (EWAS) with cord blood found a strong association between DNA methylation pattern and postnatal BMI trajectory until adolescent [100].

We also tested if maternal pre-pregnancy obesity can provide quantitative support to the long-speculated theory of the utero origin of cancers[10,11]. In particular, some researchers hypothesized there exist higher stem cell burdens in newborn babies born from obese mothers [12]. Here we provide evidence that such stem cell burden is highly likely due to intergenerational DNA methylation modification on some key biological functions (cell cycle, ribosome function, and immune response) in the uHSCs. We built a random forest model trained on 61 maternal obesity-associated CpG markers in uHSCs and applied it to predict tumor and normal tissues across 14

TCGA cancer types, without prior cancer-specific training. This model achieved decent balanced accuracy above 0.6 for 5 out of 14 TCGA cancers investigated: LUSC, PAAD, BRCA KIRP and KIRC, reflecting its cross-context predictive potential. These cancers, characterized by inflammation, immune dysregulation, and epigenetic disruption, align with pathways enriched in the obesity-associated markers [101,102]. Uncontrolled cell division, immune evasion, and chronic inflammation are well-established hallmarks of cancer [103], and these featured 61 CpG sites were implicated in relevant biological pathways that were intimately connected with cancer development. Aforehand results revealed a significant increase in stemness scores among uHSCs affected by maternal obesity, which aligns with the observed hypermethylation and subsequent downregulation of key cell cycle genes. This heightened stemness may predispose these cells to malignant transformation if these epigenetic modifications persist, leading to an elevated stem cell burden, disrupting normal cell cycle control, weakening immune surveillance, and ultimately increasing susceptibility to cancer. While performance was lower for other cancers, likely due to the small sample size limiting the detection of additional CpG biomarkers, tissue-specific methylation variability and microenvironment differences [104], the maternal obesity model implicates its biological relevance across diverse cancers.

This study is the first to directly examine the granular changes in the stem cell population of cord blood from babies of pre-pregnant obese mothers using a multi-omics approach. Previously, the association between maternal obesity and epigenetic modifications has been investigated across various tissue types (e.g., adipose tissue, liver, cord blood) and species (e.g., human, mouse) [105]. We cross-checked our findings with these reports, many of which align providing further validation of their biological significance. For example, 33 CpGs across 20 genes, such as those in TAPBP (cg17621507, cg23922433, cg27385940), TNFAIP8 (cg18689486, cg07376834,

cg03723497,cg21130861), and AGPAT1 (cg09043226, cg25733934, cg08049198, cg18191873) in our study are consistent with the cord blood leukocyte DNA methylation study from Martin et al with the same study objective [106]. TAPBP, TNFAIP8, and AGPAT1 play roles in immune function, transcriptional regulation, and lipid metabolism respectively. Additionally, our analysis also identified different CpG probes within the same genes previously associated with maternal obesity, offering additional insights into their epigenetic regulation. For instance, we observed different CpGs in HLA-E (cg01462744, cg02942965, cg26175526), ALPK1 (cg04779144, cg10855342), and PTEN (cg09472211). These genes were also reported from the Boston Birth Cohort study [107]. We identified different hypermethylation sites on MYT1L (cg05786278, cg17519749, cg21239227) and IGF1R (cg01284192, cg06596307, cg08138544, cg16918683, cg26577252), genes; these genes also showed high methylation levels in the cord blood (on different CpG sites) reported by Josefson et al [108]. Additionally, in our gene expression and methylation correlation analysis of uHSC, HOXA9 and HOXA5 emerged as the top genes (**Supplemental Figure 3D**), displaying strong correlations between expression and methylation levels. These hypomethylated genes (HOX family genes), along with 25 additional commonly identified genes, are consistent with the finding in the DNA methylation study on leukocytes of cord blood samples [109], linking them to maternal lipid and cholesterol levels. In our study, HDAC4 and PLEC1 stand out for their strong associations with obesity-related traits among the top differentially methylated CpG sites. Hypermethylation of cg05995464 in HDAC4 was previously reported to be associated with childhood obesity [110]. PLEC1 is a critical gene for extracellular matrix remodeling in adipose tissue, and hypomethylation of cg20784950 in PLEC1 is evident in our study. Lower PLEC1 methylation was previously correlated with higher BMI

and obesity status [83,111]. Together, these comparisons underscore the robust and overlapping epigenetic patterns associated with maternal pre-pregnancy obesity.

There are some caveats of this study related to the study design. First, this is a single-site study with a relatively small sample size, and along with some genomic inflation the statistical power of the findings is limited. This is especially the case for the gene expression omic layer, where individual DE genes are lacking. This may have limited maternal obesity CpG biomarker identification, which resulted in positive risk associations in some, but not all of the 14 TCGA cancers, in the classification model (**Figure 6**). When the budget allows, a large-scale multi-site population study is desirable. Secondly, we use the stem cell population in the cord blood as the surrogate for “stemness” property investigation, to link the offspring’s disease with maternal obesity. It is most feasible and practical to collect cord blood cells, and the painstaking measurements of the uHSC population avoid blood cell type heterogeneity issues, which may confound the cord blood DNA methylation result significantly [81]. However, this approach may very well be simplified and biased, as stem cells exist in many body parts of babies. Therefore, extrapolations from uHSC need to be cautioned. Further, our phenotypic data collection focused on the physiological and demographic information and missed socioeconomic data. Thirdly, environmental, lifestyle or social determinants may act as confounders and influence the observed outcomes, which are not measured nor adjustable in the study, per the protocol. Some of these measurements, such as lifestyle and health insurance, can be mitigated by incorporating electronic health record data, similar to what we have done [112,113]. Additionally, an important aspect of maternal-offspring study is to longitudinally follow them up for health outcomes later in life. The IRB for this study was not designed for such an investigation, unfortunately.

Nevertheless, this uHSC multi-omics study provides a critical initial lens peeking into the immune-metabolic mechanisms, which serves as the foundation for all the possible expansion work mentioned above.

Conclusion

In summary, this newborn study demonstrates the direct impact of maternal pre-pregnancy obesity and on newborn blood at the multi-omics level, which includes increased cell cycle arrest, impairment in the uHSCs differentiation capacity, more inflammation, and disruption in lipid metabolism. We also showed maternal obesity-associated epigenetic modifications are closely related to cancer markers, which could potentially help mitigate the intergenerational health risks.

Disclosure of use of AI-assisted tools including generative AI

During the preparation of this work the author(s) used GPT-4.0 in order to improve the readability. Prompts used in GPT-4.0 include “help me improve my writing in a more logical and professional way” and “help me correct the grammar” along with a paragraph of the author's own writing. After using this tool/service, the author(s) reviewed and edited the content thoroughly and take(s) full responsibility for the content of the publication.

Data availability statement

DNA methylation data and bulk RNA-seq data generated in this study have been submitted and will be available through the National Institutes of Health Gene Expression Omnibus (GEO) with the accession number GSE273075 (GEO reviewer token: upmtkoygrlwtrmb). Other datasets

used in this project for the analysis and validation purpose are publicly available. The placenta datasets used in this article are available in the GEO repository with accession numbers GSE31781, GSE36829, GSE59274, GSE44667, GSE74738, GSE49343, GSE69502, and GSE98224. Cord blood metabolomics data used in this article is available in metabolomics workbench with study ID ST001114. Cancer methylation datasets for BLCA, BRCA, COAD, ESCA, HNSC, KIRC, KIRP, LIHC, LUAD, LUSC, PAAD, PRAD, THCA, UCEC are available in The Cancer Genome Atlas (TCGA data portal: https://portal.gdc.cancer.gov/).

Availability of source code and requirements

Project name: COBRE Hawaii Maternal Obesity Study

Project home page: https://github.com/lanagarmire/COBRE_methyl

Operating system(s): Windows, macOS, Linux

Programming language: R, Python

Other requirements: R ≥ 4.1.0

License: GNU-GPL-3.0

Code to produce the analyses in this manuscript are available through GitHub (https://github.com/lanagarmire/COBRE_methyl)

Acknowledgments

This research was supported by grants R01 LM012373 and R01 LM012907 awarded by NLM, and R01 HD084633 awarded by NICHD to L.X. Garmire, as well as in part by the NCI Cancer Center Support Grant (CCSG) number P30 CA071789 awarded to Genomics and Bioinformatics Shared Resource (RRID:SCR_019085). This research was supported in part by training funding

provided by the NIH grant T32 GM141746 and Advanced Proteogenomics of Cancer (T32 CA140044).

Author contributions

LG envisioned this project, obtained the funding, supervised the study and revised the manuscript. YD performed the data analysis, generated the figures, and wrote the initial manuscript. YS collected TCGA data and built the machine learning models. RS consented patients and obtained the samples from the hospital, with coordination from PB. DT and SW coordinated with the patient recruitment and study. PB coordinated all the multi-omics assays. PB, CL, and FA designed the DNA methylation assays. AG performed FACS sorting of cord blood cells. ALJ performed the Illumina Meth 450 assay, MT supervised the Genomics Shared Resource analyses and provided a critical review of the manuscript. All authors have read the manuscript.

Conflicts of interest

None

References

1. Leddy, M. A., Power, M. L. & Schulkin, J. The impact of maternal obesity on maternal and fetal health. *Rev. Obstet. Gynecol.* **1**, 170–178 (2008).
2. Hjalgrim, L. L. *et al.* Birth weight as a risk factor for childhood leukemia: a meta-analysis of 18 epidemiologic studies. *Am. J. Epidemiol.* **158**, 724–735 (2003).
3. Harder, T., Plagemann, A. & Harder, A. Birth weight and risk of neuroblastoma: a meta-analysis. *Int. J. Epidemiol.* **39**, 746–756 (2010).
4. Cnattingius, S., Lundberg, F., Sandin, S., Grönberg, H. & Iliadou, A. Birth characteristics and risk of prostate cancer: the contribution of genetic factors. *Cancer Epidemiol. Biomarkers Prev.* **18**, 2422–2426 (2009).
5. Eriksson, M. *et al.* The impact of birth weight on prostate cancer incidence and mortality in a population-based study of men born in 1913 and followed up from 50 to 85 years of age. *Prostate* **67**, 1247–1254 (2007).
6. Michos, A., Xue, F. & Michels, K. B. Birth weight and the risk of testicular cancer: a meta-analysis. *Int. J. Cancer* **121**, 1123–1131 (2007).
7. Silva, I. dos S., De Stavola, B., McCormack, V. & Collaborative Group on Pre-Natal Risk Factors and Subsequent Risk of Breast Cancer. Birth size and breast cancer risk: re-analysis of individual participant data from 32 studies. *PLoS Med.* **5**, e193 (2008).
8. Van Cleave, J., Gortmaker, S. L. & Perrin, J. M. Dynamics of obesity and chronic health

conditions among children and youth. *JAMA* **303**, 623–630 (2010).

9. Godfrey, K. M. *et al.* Influence of maternal obesity on the long-term health of offspring. *The lancet. Diabetes & endocrinology* **5**, (2017).
10. Barker, D. J. The origins of the developmental origins theory. *J. Intern. Med.* **261**, (2007).
11. Barker, D. J. In utero programming of cardiovascular disease. *Theriogenology* **53**, (2000).
12. Qiu, L. *et al.* Novel measurements of mammary stem cells in human umbilical cord blood as prospective predictors of breast cancer susceptibility in later life. *Ann. Oncol.* **23**, 245–250 (2012).
13. Savarese, T. M. *et al.* Correlation of umbilical cord blood hormones and growth factors with stem cell potential: implications for the prenatal origin of breast cancer hypothesis. *Breast Cancer Res.* **9**, R29 (2007).
14. Marshall, G. M. *et al.* The prenatal origins of cancer. *Nat. Rev. Cancer* **14**, 277–289 (2014).
15. Fábián, Á., Vereb, G. & Szöllősi, J. The hitchhikers guide to cancer stem cell theory: markers, pathways and therapy. *Cytometry A* **83**, 62–71 (2013).
16. Tan, B. T., Park, C. Y., Ailles, L. E. & Weissman, I. L. The cancer stem cell hypothesis: a work in progress. *Lab. Invest.* **86**, 1203–1207 (2006).
17. Strohsnitter, W. C. *et al.* Correlation of umbilical cord blood haematopoietic stem and progenitor cell levels with birth weight: implications for a prenatal influence on cancer risk. *Br. J. Cancer* **98**, 660–663 (2008).
18. Apgar, V. A Proposal for a New Method of Evaluation of the Newborn Infant. *Anesthesia & Analgesia* **32**, 260 (1953).
19. Aryee, M. J. *et al.* Minfi: a flexible and comprehensive Bioconductor package for the analysis of Infinium DNA methylation microarrays. *Bioinformatics* **30**, 1363 (2014).

20. Morris, T. J. *et al.* ChAMP: 450k Chip Analysis Methylation Pipeline. *Bioinformatics* **30**, 428–430 (2014).
21. Fortin, J.-P., Triche, T. J., Jr & Hansen, K. D. Preprocessing, normalization and integration of the Illumina HumanMethylationEPIC array with minfi. *Bioinformatics* **33**, 558–560 (2017).
22. Zhou, W., Laird, P. W. & Shen, H. Comprehensive characterization, annotation and innovative use of Infinium DNA methylation BeadChip probes. *Nucleic Acids Res.* **45**, e22 (2017).
23. Teschendorff, A. E. *et al.* A beta-mixture quantile normalization method for correcting probe design bias in Illumina Infinium 450 k DNA methylation data. *Bioinformatics* **29**, 189–196 (2013).
24. Chen, Y. A. *et al.* Discovery of cross-reactive probes and polymorphic CpGs in the Illumina Infinium HumanMethylation450 microarray. *Epigenetics* **8**, (2013).
25. Du, P., Kibbe, W. A. & Lin, S. M. nuID: a universal naming scheme of oligonucleotides for illumina, affymetrix, and other microarrays. *Biol. Direct* **2**, 16 (2007).
26. Lin, S. M., Du, P., Huber, W. & Kibbe, W. A. Model-based variance-stabilizing transformation for Illumina microarray data. *Nucleic Acids Res.* **36**, e11 (2008).
27. Du, P., Kibbe, W. A. & Lin, S. M. lumi: a pipeline for processing Illumina microarray. *Bioinformatics* **24**, 1547–1548 (2008).
28. Du, P. *et al.* Comparison of Beta-value and M-value methods for quantifying methylation levels by microarray analysis. *BMC Bioinformatics* **11**, 587 (2010).
29. He, B. *et al.* The maternal blood lipidome is indicative of the pathogenesis of severe preeclampsia. *J. Lipid Res.* **62**, (2021).

30. Chen, Y. *et al.* Maternal plasma lipids are involved in the pathogenesis of preterm birth. *Gigascience* **11**, (2022).
31. van Iterson, M., van Zwet, E. W. & Heijmans, B. T. Controlling bias and inflation in epigenome- and transcriptome-wide association studies using the empirical null distribution. *Genome Biology* **18**, 1–13 (2017).
32. Leek, J. T., Johnson, W. E., Parker, H. S., Jaffe, A. E. & Storey, J. D. The sva package for removing batch effects and other unwanted variation in high-throughput experiments. *Bioinformatics (Oxford, England)* **28**, (2012).
33. Ritchie, M. E. *et al.* limma powers differential expression analyses for RNA-sequencing and microarray studies. *Nucleic Acids Res.* **43**, e47 (2015).
34. Jaffe, A. E. *et al.* Bump hunting to identify differentially methylated regions in epigenetic epidemiology studies. *Int. J. Epidemiol.* **41**, 200–209 (2012).
35. Novakovic, B. *et al.* Evidence for widespread changes in promoter methylation profile in human placenta in response to increasing gestational age and environmental/stochastic factors. *BMC Genomics* **12**, (2011).
36. GEO Accession viewer. https://www.ncbi.nlm.nih.gov/geo/query/acc.cgi?acc=GSE36829.
37. Chu, T. *et al.* Comprehensive analysis of preeclampsia-associated DNA methylation in the placenta. *PLoS One* **9**, (2014).
38. Blair, J. D. *et al.* Widespread DNA hypomethylation at gene enhancer regions in placentas associated with early-onset pre-eclampsia. *Mol. Hum. Reprod.* **19**, (2013).
39. Hanna, C. W. *et al.* Pervasive polymorphic imprinted methylation in the human placenta. *Genome Res.* **26**, (2016).
40. Blair, J. D., Langlois, S., McFadden, D. E. & Robinson, W. P. Overlapping DNA

methylation profile between placentas with trisomy 16 and early-onset preeclampsia. *Placenta* **35**, (2014).

41. Price, E. M. *et al.* Profiling placental and fetal DNA methylation in human neural tube defects. *Epigenetics Chromatin* **9**, (2016).

42. Leavey, K., Wilson, S. L., Bainbridge, S. A., Robinson, W. P. & Cox, B. J. Epigenetic regulation of placental gene expression in transcriptional subtypes of preeclampsia. *Clin. Epigenetics* **10**, (2018).

43. Wilson, S. L., Leavey, K., Cox, B. J. & Robinson, W. P. Mining DNA methylation alterations towards a classification of placental pathologies. *Hum. Mol. Genet.* **27**, (2018).

44. Hansen, K. D. IlluminaHumanMethylation450kanno. ilmn12. hg19: annotation for Illumina's 450k methylation arrays. *R package version 0.6. 0.*

45. Maksimovic, J., Gordon, L. & Oshlack, A. SWAN: Subset-quantile within array normalization for illumina infinium HumanMethylation450 BeadChips. *Genome Biol.* **13**, R44 (2012).

46. Phipson, B. & Oshlack, A. DiffVar: a new method for detecting differential variability with application to methylation in cancer and aging. *Genome Biol.* **15**, 465 (2014).

47. Maksimovic, J., Gagnon-Bartsch, J. A., Speed, T. P. & Oshlack, A. Removing unwanted variation in a differential methylation analysis of Illumina HumanMethylation450 array data. *Nucleic Acids Res.* **43**, e106 (2015).

48. Phipson, B., Maksimovic, J. & Oshlack, A. missMethyl: an R package for analyzing data from Illumina's HumanMethylation450 platform. *Bioinformatics* **32**, 286–288 (2016).

49. Kanehisa, M. & Goto, S. KEGG: kyoto encyclopedia of genes and genomes. *Nucleic Acids Res.* **28**, 27–30 (2000).

50. Kanehisa, M. Toward understanding the origin and evolution of cellular organisms. *Protein Sci.* **28**, 1947–1951 (2019).

51. Kanehisa, M., Furumichi, M., Sato, Y., Kawashima, M. & Ishiguro-Watanabe, M. KEGG for taxonomy-based analysis of pathways and genomes. *Nucleic Acids Res.* (2022) doi:10.1093/nar/gkac963.

52. Langfelder, P. & Horvath, S. WGCNA: an R package for weighted correlation network analysis. *BMC Bioinformatics* **9**, 559 (2008).

53. Langfelder, P. & Horvath, S. Fast R Functions for Robust Correlations and Hierarchical Clustering. *J. Stat. Softw.* **46**, (2012).

54. Szklarczyk, D. *et al.* The STRING database in 2021: customizable protein-protein networks, and functional characterization of user-uploaded gene/measurement sets. *Nucleic Acids Res.* **49**, D605–D612 (2021).

55. Li, H. *et al.* PAND: A Distribution to Identify Functional Linkage from Networks with Preferential Attachment Property. *PLoS One* **10**, e0127968 (2015).

56. Shannon, P. *et al.* Cytoscape: a software environment for integrated models of biomolecular interaction networks. *Genome Res.* **13**, 2498–2504 (2003).

57. He, B. & Garmire, L. X. ASGARD: A Single-cell Guided pipeline to Aid Repurposing of Drugs. *ArXiv* (2021).

58. MacArthur, B. D. & Lemischka, I. R. Statistical mechanics of pluripotency. *Cell* **154**, 484–489 (2013).

59. Martínez, O. & Reyes-Valdés, M. H. Defining diversity, specialization, and gene specificity in transcriptomes through information theory. *Proc. Natl. Acad. Sci. U. S. A.* **105**, 9709–9714 (2008).

60. Kannan, S., Farid, M., Lin, B. L., Miyamoto, M. & Kwon, C. Transcriptomic entropy benchmarks stem cell-derived cardiomyocyte maturation against endogenous tissue at single cell level. *PLoS Comput. Biol.* **17**, e1009305 (2021).
61. Bushnell, B. BBMap: A Fast, Accurate, Splice-Aware Aligner. (2014).
62. Andrews, S. FastQC: a quality control tool for high throughput sequence data. Available online. *Retrieved May*.
63. Dobin, A. *et al.* STAR: ultrafast universal RNA-seq aligner. *Bioinformatics* **29**, 15–21 (2013).
64. Liao, Y., Smyth, G. K. & Shi, W. featureCounts: an efficient general purpose program for assigning sequence reads to genomic features. *Bioinformatics* **30**, 923–930 (2014).
65. Liao, Y., Smyth, G. K. & Shi, W. The Subread aligner: fast, accurate and scalable read mapping by seed-and-vote. *Nucleic Acids Res.* **41**, e108 (2013).
66. Law, C. W., Chen, Y., Shi, W. & Smyth, G. K. voom: precision weights unlock linear model analysis tools for RNA-seq read counts. *Genome Biology* **15**, 1–17 (2014).
67. Love, M. I., Huber, W. & Anders, S. Moderated estimation of fold change and dispersion for RNA-seq data with DESeq2. *Genome Biol.* **15**, 550 (2014).
68. Chen, J., Xu, H., Aronow, B. J. & Jegga, A. G. Improved human disease candidate gene prioritization using mouse phenotype. *BMC Bioinformatics* **8**, 392 (2007).
69. Chen, J., Aronow, B. J. & Jegga, A. G. Disease candidate gene identification and prioritization using protein interaction networks. *BMC Bioinformatics* **10**, 73 (2009).
70. Chen, J., Bardes, E. E., Aronow, B. J. & Jegga, A. G. ToppGene Suite for gene list enrichment analysis and candidate gene prioritization. *Nucleic Acids Res.* **37**, W305–11 (2009).

71. Schlueter, R. J. *et al.* Prepregnant Obesity of Mothers in a Multiethnic Cohort Is Associated with Cord Blood Metabolomic Changes in Offspring. *J. Proteome Res.* **19**, 1361–1374 (2020).

72. Johnson, W. E., Li, C. & Rabinovic, A. Adjusting batch effects in microarray expression data using empirical Bayes methods. *Biostatistics* **8**, 118–127 (2006).

73. Singh, A. *et al.* DIABLO: an integrative approach for identifying key molecular drivers from multi-omics assays. *Bioinformatics* **35**, 3055 (2019).

74. Luo, W. & Brouwer, C. Pathview: an R/Bioconductor package for pathway-based data integration and visualization. *Bioinformatics* **29**, 1830–1831 (2013).

75. Fang, X. *et al.* Lilikoi V2.0: a deep learning-enabled, personalized pathway-based R package for diagnosis and prognosis predictions using metabolomics data. *Gigascience* **10**, (2021).

76. Al-Akwaa, F. M., Yunits, B., Huang, S., Alhajaji, H. & Garmire, L. X. Lilikoi: an R package for personalized pathway-based classification modeling using metabolomics data. *GigaScience* **7**, (2018).

77. Heard, E. & Martienssen, R. A. Transgenerational epigenetic inheritance: myths and mechanisms. *Cell* **157**, 95–109 (2014).

78. King, S. E. & Skinner, M. K. Epigenetic Transgenerational Inheritance of Obesity Susceptibility. *Trends Endocrinol. Metab.* **31**, 478–494 (2020).

79. Morisako, A. K., Tauali‘i, M., Ambrose, A. J. H. & Withy, K. Beyond the Ability to Pay: The Health Status of Native Hawaiians and Other Pacific Islanders in Relationship to Health Insurance. *Hawaii J. Med. Public Health* **76**, 36 (2017).

80. Heslehurst, N. *et al.* The association between maternal body mass index and child obesity:

A systematic review and meta-analysis. *PLoS Med.* **16**, e1002817 (2019).
81. Liu, W. *et al.* Severe preeclampsia is not associated with significant DNA methylation changes but cell proportion changes in the cord blood - caution on the importance of confounding adjustment. *medRxiv* 2023.08.31.23294898 (2023) doi:10.1101/2023.08.31.23294898.
82. Abu-Farha, M. *et al.* Proteomics Analysis of Human Obesity Reveals the Epigenetic Factor HDAC4 as a Potential Target for Obesity. *PLoS One* **8**, (2013).
83. Rönn, T. *et al.* Impact of age, BMI and HbA1c levels on the genome-wide DNA methylation and mRNA expression patterns in human adipose tissue and identification of epigenetic biomarkers in blood. *Human molecular genetics* **24**, (2015).
84. Lim, W.-J., Kim, K. H., Kim, J.-Y., Jeong, S. & Kim, N. Identification of DNA-Methylated CpG Islands Associated With Gene Silencing in the Adult Body Tissues of the Ogye Chicken Using RNA-Seq and Reduced Representation Bisulfite Sequencing. *Front. Genet.* **10**, 346 (2019).
85. Ching, T. *et al.* Genome-wide hypermethylation coupled with promoter hypomethylation in the chorioamniotic membranes of early onset pre-eclampsia. *Mol. Hum. Reprod.* **20**, 885–904 (2014).
86. Meng, D., Frank, A. R. & Jewell, J. L. mTOR signaling in stem and progenitor cells. *Development* **145**, (2018).
87. Guo, M., Bao, E. L., Wagner, M., Whitsett, J. A. & Xu, Y. SLICE: determining cell differentiation and lineage based on single cell entropy. *Nucleic Acids Res.* **45**, e54 (2017).
88. Dai, D. *et al.* Planar cell polarity effector gene Intu regulates cell fate-specific differentiation of keratinocytes through the primary cilia. *Cell Death Differ.* **20**, 130–138

(2012).

89. Liu, J. & Fuchs, S. Y. Cross-talk between APC/C and CBP/p300. *Cancer Biol. Ther.* **5**, (2006).

90. Corbi, S. C. T. *et al.* Expression Profile of Genes Potentially Associated with Adequate Glycemic Control in Patients with Type 2 Diabetes Mellitus. *Journal of diabetes research* **2017**, (2017).

91. Behl, T. *et al.* Exploring the Genetic Conception of Obesity via the Dual Role of FoxO. *Int. J. Mol. Sci.* **22**, (2021).

92. Kadakia, R. *et al.* Maternal pre-pregnancy BMI downregulates neonatal cord blood LEP methylation. *Pediatr. Obes.* **12 Suppl 1**, 57–64 (2017).

93. Yu, Z. *et al.* Pre-pregnancy body mass index in relation to infant birth weight and offspring overweight/obesity: a systematic review and meta-analysis. *PLoS One* **8**, e61627 (2013).

94. Sureshchandra, S., Marshall, N. E. & Messaoudi, I. Impact of pregravid obesity on maternal and fetal immunity: Fertile grounds for reprogramming. *J. Leukoc. Biol.* **106**, 1035–1050 (2019).

95. Levy, O. Innate immunity of the human newborn: distinct cytokine responses to LPS and other Toll-like receptor agonists. *J. Endotoxin Res.* **11**, 113–116 (2005).

96. Ong, P. S. *et al.* Judicious Toggling of mTOR Activity to Combat Insulin Resistance and Cancer: Current Evidence and Perspectives. *Front. Pharmacol.* **7**, (2016).

97. Mack, R., Zhang, L., Breslin, P. & Zhang, J. The fetal-to-adult hematopoietic stem cell transition and its role in childhood hematopoietic malignancies. *Stem cell reviews and reports* **17**, 2059 (2021).

98. Kwok, A. C. & Wong, J. T. Lipid biosynthesis and its coordination with cell cycle

progression. *Plant Cell Physiol.* **46**, (2005).

99. Denizli, M., Capitano, M. L. & Kua, K. L. Maternal obesity and the impact of associated early-life inflammation on long-term health of offspring. *Front. Cell. Infect. Microbiol.* **12**, 940937 (2022).

100. Meir, A. Y. *et al.* Umbilical cord DNA methylation is associated with body mass index trajectories from birth to adolescence. *EBioMedicine* **91**, 104550 (2023).

101. Gukovsky, I., Li, N., Todoric, J., Gukovskaya, A. & Karin, M. Inflammation, Autophagy, and Obesity: Common Features in the Pathogenesis of Pancreatitis and Pancreatic Cancer. *Gastroenterology* **144**, 1199 (2013).

102. Sanchez, A. *et al.* Transcriptomic signatures related to the obesity paradox in patients with clear cell renal cell carcinoma: a retrospective cohort study. *The Lancet. Oncology* **21**, 283 (2019).

103. Hanahan, D. & Weinberg, R. A. Hallmarks of Cancer: The Next Generation. *Cell* **144**, 646–674 (2011).

104. Jones, P. A. & Baylin, S. B. The epigenomics of cancer. *Cell* **128**, (2007).

105. Zhao, D. *et al.* Influence of maternal obesity on the multi-omics profiles of the maternal body, gestational tissue, and offspring. *Biomedicine & pharmacotherapy = Biomedecine & pharmacotherapie* **151**, (2022).

106. Martin, C. L. *et al.* Maternal pre-pregnancy obesity, offspring cord blood DNA methylation, and offspring cardiometabolic health in early childhood: an epigenome-wide association study. *Epigenetics* **14**, (2019).

107. Si, J. *et al.* Maternal pre-pregnancy BMI, offspring epigenome-wide DNA methylation, and childhood obesity: findings from the Boston Birth Cohort. *BMC Medicine* **21**, 1–13 (2023).

108. Josefson, J. L. *et al.* Newborn adiposity is associated with cord blood DNA methylation at IGF1R and KLF7. *Obesity* **32**, 1923–1933 (2024).

109. Waldrop, S. W. *et al.* Cord blood DNA methylation of immune and lipid metabolism genes is associated with maternal triglycerides and child adiposity. *Obesity* **32**, 187–199 (2024).

110. Li, Y. *et al.* Genome-wide analysis reveals that altered methylation in specific CpG loci is associated with childhood obesity. *Journal of cellular biochemistry* **119**, (2018).

111. Crujeiras, A. B. *et al.* An Epigenetic Signature in Adipose Tissue Is Linked to Nicotinamide N-Methyltransferase Gene Expression. *Molecular nutrition & food research* **62**, (2018).

112. Ballard, H. K., Yang, X., Mahadevan, A. D., Lemas, D. J. & Garmire, L. X. Five-Feature Models to Predict Preeclampsia Onset Time From Electronic Health Record Data: Development and Validation Study. *Journal of medical Internet research* **26**, (2024).

113. Zhu, H. *et al.* Discover overlooked complications after preeclampsia from three real-world medical record datasets of over 100,000 pregnancies. *medRxiv* 2023.12.05.23299296 (2024) doi:10.1101/2023.12.05.23299296.

114. Oesterreich, S. *et al.* High rates of loss of heterozygosity on chromosome 19p13 in human breast cancer. *Br. J. Cancer* **84**, 493–498 (2001).

115. Hong, E. A., Gautrey, H. L., Elliott, D. J. & Tyson-Capper, A. J. SAFB1- and SAFB2-mediated transcriptional repression: relevance to cancer. *Biochem. Soc. Trans.* **40**, 826–830 (2012).

116. Hammerich-Hille, S. *et al.* SAFB1 mediates repression of immune regulators and apoptotic genes in breast cancer cells. *J. Biol. Chem.* **285**, 3608–3616 (2010).

Figure legends

Figure 1. Overview of the study design and analysis. In the preparation step, cord blood plasma samples are collected for metabolome profiling and stem cell sorting. DNA and RNA extraction assays are performed on the enriched stem cells for the methylation and RNA-seq analyses. Downstream analyses are mainly focused on the methylation data. Bulk RNA-seq data were used for validations for methylation discoveries. (Created with BioRender.com)

Figure 2. Mother and newborns statistics of the multi-ethnic cohort from Hawaii. (**A-E**) Categorical variables including baby sex, maternal ethnicity, paternal ethnicity, parity and gravidity between control and obese groups are shown in the barplots. P-values using Chi-square test are annotated comparing control and obese groups. **(F-I**) The distributions of maternal age, gestation age, maternal net weight gain during pregnancy, and maternal hemoglobin between control and obese groups are compared. Mean and standard deviation are shown in boxplot. P-values using t-test are annotated. (**J-M**) The distributions of baby weight, baby head circumference, baby length, and APGAR score after 5 minutes of delivery between control and obese groups are compared. Mean and standard deviation are shown in boxplot. P-values using t-test are annotated.

Figure 3. DNA methylation analysis on uHSCs.
(**A-B**) Source of variance plot before and after confounding adjustment. F-statistics are reported for each clinical factor. F statistics greater than 1 are considered to have confounding effects in addition to the case/control difference due to pre-pregnancy maternal obesity. (**C**) Volcano plot

of -log(BH adjusted p-values) against logFC. The cutoff line for adjusted p-value < 0.05 is shown as the red horizontal line. The hyper/hypo threshold is shown as a blue vertical line where logFC=0. Non-significant methylation CpG sites after the differential analysis were shown in gray. Significant CpG sites are colored. (**D-E**) Normalized location distribution of differentially methylated CpG sites according to their CpG features in terms of isle regions and gene regions based on the chip annotation.

Figure 4. Pathway and network analysis. (**A**) KEGG pathway enrichment for hypermethylated CpG sites from promotor region. Enriched KEGG pathway names, adjusted p-values (-log10 transformed), and the size of enriched gene list are reported for CpG sites from TSS200+TSS1500 regions. The red dotted line shows the threshold cutoff for FDR at -log10(0.05). (**B-C**) Violin plots of averaged beta values for KEGG protein pathway collection and immune pathway collection with Wilcoxon P-values. (**D**) Violin plots of cell entropy scores between control and obese groups with Wilcoxon P-values. (**E-F**) WGCNA network analysis results. WGCNA modules are shown for both the control and the obese group. The top two modules with largest degrees are turquoise and brown modules. Each node represents a gene. Genes co-expressed in each module are annotated. (**G**) Protein-protein interaction (PPI) network. Bipartite graphs represent enriched KEGG pathways and associated genes with significant PPIs. Red nodes represent genes with hypermethylated CpG sites. Blue nodes represent genes with hypomethylated CpG sites. Yellow nodes represented the enriched KEGG pathways. Number of inter-pathway PPIs are annotated in the rectangular boxes.

Figure 5. Multi-omics integration analysis

(**A-C**) Omics-specific sample plots from DIABLO showing the separation of obese and control samples in methylation data, gene expression data, and metabolomics data respectively. (**D-F**) Importance plot of top 25 features in methylation, gene expression and metabolomics modalities with the highest loadings extracted from the embedding space. The color represents the condition which features contribute the most.

Figure 6. TCGA cancer classification by the maternal obesity classification model

(**A**) Receiver operating characteristic (ROC) curves on the 14 TCGA cancers, using the random forest classification model built with the CpG sites of genes in the top 5 pathways associated with maternal pre-pregnancy obesity. Balanced accuracy is shown for each cancer type. (**B**) Barplots showing the prediction performances on these TCGA datasets, using AUC, Balanced Accuracy, and F1 score.

Figure 7. A proposed conceptual model of maternal obesity's impact on neonatal development.

Tables

Table 1. Summary statistics of the study cohort.

		Control (n=38)	Case (n=34)
Maternal Age		31.3±5.6	31.6±4.9
Gestational Week		38.9±0.5	39.0±0.3
Net Weight Gain		32.0±11.6	30.9±14.6
Hemoglobin		11.6±1.6	11.0±1.4
Maternal Ethnicity	Asian	21	8
	Caucasian	11	4
	NHPI	6	22
Paternal Ethnicity	Asian	19	11
	Caucasian	11	2
	NHPI	8	21
Baby Sex	Female	17	21
	Male	21	13
Parity	0	7	3
	1	21	7
	2	9	11
	More	1	13
Gravidity	1	6	2
	2	15	5
	3	13	8
	4	3	6
	5	1	4
	More	0	9

Demographic and clinical statistics are reported for the control and maternally obese groups.

Table 2. Top 20 hypermethylated CpG sites and top 20 hypomethylated CpG sites.

CpG	Gene	Island	Group	logFC	P.Value	adj.P.Val	Type
cg12303247	SYT11	OpenSea	3'UTR	2.188	2.44E-05	6.08E-03	Hyper
cg16818768	PSMG1	Island	TSS1500	1.605	2.15E-05	5.74E-03	Hyper
cg05995465	HDAC4	OpenSea	5'UTR	1.604	1.64E-03	4.64E-02	Hyper
cg01937701	DHRS4	Island	TSS200	1.592	1.95E-10	2.43E-05	Hyper
cg22243583	DLEU1	S_Shore	Body	1.522	2.53E-06	2.00E-03	Hyper
cg16927136	RPL35A	OpenSea	TSS1500	1.507	2.38E-10	2.43E-05	Hyper
cg08899199	ST7	S_Shore	Body	1.4	7.32E-07	1.12E-03	Hyper
cg05054115	DHRS4	Island	TSS200	1.389	6.64E-08	3.71E-04	Hyper
cg12878710	LRCH3	Island	TSS200	1.387	1.26E-06	1.47E-03	Hyper
cg05130022	HMGN4	N_Shore	TSS200	1.386	1.51E-04	1.45E-02	Hyper

cg05643303	HOXC8	Island	TSS200	1.345	2.69E-05	6.34E-03	Hyper
cg07449543	CHORDC1	S_Shore	TSS200	1.342	6.31E-05	9.53E-03	Hyper
cg25016112	DENND3	OpenSea	Body	1.314	1.22E-03	4.00E-02	Hyper
cg09552166	MSL2	N_Shore	TSS200	1.296	2.29E-05	5.92E-03	Hyper
cg01003902	SAFB2	Island	TSS200	1.269	1.03E-08	1.41E-04	Hyper
cg11028445	FAM96A	N_Shore	TSS1500	1.265	1.97E-04	1.65E-02	Hyper
cg10317138	ADAM12	N_Shore	Body	1.229	5.05E-04	2.60E-02	Hyper
cg09757277	ZNF222	S_Shore	5'UTR	1.229	9.86E-08	4.24E-04	Hyper
cg04117338	CRADD	N_Shore	5'UTR	1.209	1.66E-03	4.67E-02	Hyper
cg07354583	CD69	OpenSea	Body	1.205	5.93E-07	1.01E-03	Hyper
cg04043455	EBF3	S_Shelf	Body	-2.031	6.11E-04	2.86E-02	Hypo
cg20784950	PLEC1	N_Shore	Body	-1.812	1.96E-05	5.45E-03	Hypo

cg09976051	AGA	N_Shore	Body	-1.516	1.67E-04	1.53E-02	Hypo
cg13862711	LHX6	Island	Body	-1.469	1.65E-03	4.65E-02	Hypo
cg16434331	SLC39A11	OpenSea	Body	-1.411	9.50E-08	4.24E-04	Hypo
cg05636467	EBF3	S_Shelf	Body	-1.335	1.65E-03	4.65E-02	Hypo
cg16858146	TAF3	S_Shelf	Body	-1.33	3.14E-05	6.80E-03	Hypo
cg24796644	MDGA1	Island	Body	-1.242	1.47E-05	4.79E-03	Hypo
cg11064039	PRKAR1B	Island	5'UTR	-1.227	1.58E-03	4.56E-02	Hypo
cg06833656	TBCD	OpenSea	Body	-1.219	2.67E-06	2.05E-03	Hypo
cg25430507	NXPH2	S_Shore	TSS1500	-1.152	2.08E-06	1.87E-03	Hypo
cg03485608	NXPH2	N_Shore	Body	-1.152	2.71E-06	2.05E-03	Hypo
cg00928596	MIR365-1	OpenSea	TSS200	-1.148	7.31E-05	1.03E-02	Hypo
cg12601963	NCRNA00200	Island	Body	-1.132	2.57E-06	2.02E-03	Hypo

cg22772691	SLC12A7	S_Shelf	Body	-1.123	1.79E-04	1.57E-02	Hypo
cg02584267	EBF3	OpenSea	Body	-1.121	2.38E-04	1.80E-02	Hypo
cg19282259	NCRNA00200	Island	TSS200	-1.104	3.48E-06	2.32E-03	Hypo
cg08010094	NXPH2	S_Shore	TSS1500	-1.094	1.04E-03	3.69E-02	Hypo
cg06916001	MIR365-1	OpenSea	TSS200	-1.088	5.73E-05	9.17E-03	Hypo
cg03721387	KRTAP24-1	OpenSea	3'UTR	-1.04	4.29E-06	2.53E-03	Hypo

logFC, p-values, BH adjusted p-values, and CpG annotations are reported for the top 20 differentially hypermethylated CpG sites ordered by the adjusted p-values by ‘limma’ packages. Hypermethylated CpG sites are defined as logFC>0, whereas hypomethylated CpG sites are defined as logFC<0.

Click here to access/download;Table;Table 1.xlsx

		Control (n=38)	Case (n=34)
Maternal Age		31.3±5.6	31.6±4.9
Gestational Week		38.9±0.5	39.0±0.3
Net Weight Gain		32.0±11.6	30.9±14.6
Hemoglobin		11.6±1.6	11.0±1.4
Maternal Ethnicity	Asian	21	8
	Caucasian	11	4
	NHPI	6	22
Paternal Ethnicity	Asian	19	11
	Caucasian	11	2
	NHPI	8	21
Baby Sex	Female	17	21
	Male	21	13
Parity	0	7	3
	1	21	7
	2	9	11
	More	1	13
Gravidity	1	6	2
	2	15	5
	3	13	8
	4	3	6
	5	1	4
	More	0	9

Click here to access/download;Table;Table 2.xlsx

CpG	Gene	Island	Group	logFC
cg12303247	SYT11	OpenSea	3'UTR	2.188
cg16818768	PSMG1	Island	TSS1500	1.605
cg05995465	HDAC4	OpenSea	5'UTR	1.604
cg01937701	DHRS4	Island	TSS200	1.592
cg22243583	DLEU1	S_Shore	Body	1.522
cg16927136	RPL35A	OpenSea	TSS1500	1.507
cg08899199	ST7	S_Shore	Body	1.4
cg05054115	DHRS4	Island	TSS200	1.389
cg12878710	LRCH3	Island	TSS200	1.387
cg05130022	HMGN4	N_Shore	TSS200	1.386
cg05643303	HOXC8	Island	TSS200	1.345
cg07449543	CHORDC1	S_Shore	TSS200	1.342
cg25016112	DENND3	OpenSea	Body	1.314
cg09552166	MSL2	N_Shore	TSS200	1.296
cg01003902	SAFB2	Island	TSS200	1.269
cg11028445	FAM96A	N_Shore	TSS1500	1.265
cg10317138	ADAM12	N_Shore	Body	1.229
cg09757277	ZNF222	S_Shore	5'UTR	1.229
cg04117338	CRADD	N_Shore	5'UTR	1.209
cg07354583	CD69	OpenSea	Body	1.205
cg04043455	EBF3	S_Shelf	Body	-2.031
cg20784950	PLEC1	N_Shore	Body	-1.812
cg09976051	AGA	N_Shore	Body	-1.516
cg13862711	LHX6	Island	Body	-1.469
cg16434331	SLC39A11	OpenSea	Body	-1.411
cg05636467	EBF3	S_Shelf	Body	-1.335
cg16858146	TAF3	S_Shelf	Body	-1.33
cg24796644	MDGA1	Island	Body	-1.242
cg11064039	PRKAR1B	Island	5'UTR	-1.227
cg06833656	TBCD	OpenSea	Body	-1.219
cg25430507	NXPH2	S_Shore	TSS1500	-1.152
cg03485608	NXPH2	N_Shore	Body	-1.152
cg00928596	MIR365-1	OpenSea	TSS200	-1.148
cg12601963	NCRNA00200	Island	Body	-1.132
cg22772691	SLC12A7	S_Shelf	Body	-1.123
cg02584267	EBF3	OpenSea	Body	-1.121
cg19282259	NCRNA00200	Island	TSS200	-1.104
cg08010094	NXPH2	S_Shore	TSS1500	-1.094
cg06916001	MIR365-1	OpenSea	TSS200	-1.088
cg03721387	KRTAP24-1	OpenSea	3'UTR	-1.04

P.Value	adj.P.Val	Type
2.44E-05	6.08E-03	Hyper
2.15E-05	5.74E-03	Hyper
1.64E-03	4.64E-02	Hyper
1.95E-10	2.43E-05	Hyper
2.53E-06	2.00E-03	Hyper
2.38E-10	2.43E-05	Hyper
7.32E-07	1.12E-03	Hyper
6.64E-08	3.71E-04	Hyper
1.26E-06	1.47E-03	Hyper
1.51E-04	1.45E-02	Hyper
2.69E-05	6.34E-03	Hyper
6.31E-05	9.53E-03	Hyper
1.22E-03	4.00E-02	Hyper
2.29E-05	5.92E-03	Hyper
1.03E-08	1.41E-04	Hyper
1.97E-04	1.65E-02	Hyper
5.05E-04	2.60E-02	Hyper
9.86E-08	4.24E-04	Hyper
1.66E-03	4.67E-02	Hyper
5.93E-07	1.01E-03	Hyper
6.11E-04	2.86E-02	Hypo
1.96E-05	5.45E-03	Hypo
1.67E-04	1.53E-02	Hypo
1.65E-03	4.65E-02	Hypo
9.50E-08	4.24E-04	Hypo
1.65E-03	4.65E-02	Hypo
3.14E-05	6.80E-03	Hypo
1.47E-05	4.79E-03	Hypo
1.58E-03	4.56E-02	Hypo
2.67E-06	2.05E-03	Hypo
2.08E-06	1.87E-03	Hypo
2.71E-06	2.05E-03	Hypo
7.31E-05	1.03E-02	Hypo
2.57E-06	2.02E-03	Hypo
1.79E-04	1.57E-02	Hypo
2.38E-04	1.80E-02	Hypo
3.48E-06	2.32E-03	Hypo
1.04E-03	3.69E-02	Hypo
5.73E-05	9.17E-03	Hypo
4.29E-06	2.53E-03	Hypo

Figure 1

Click here to access/download;Figure;Figure 1.png

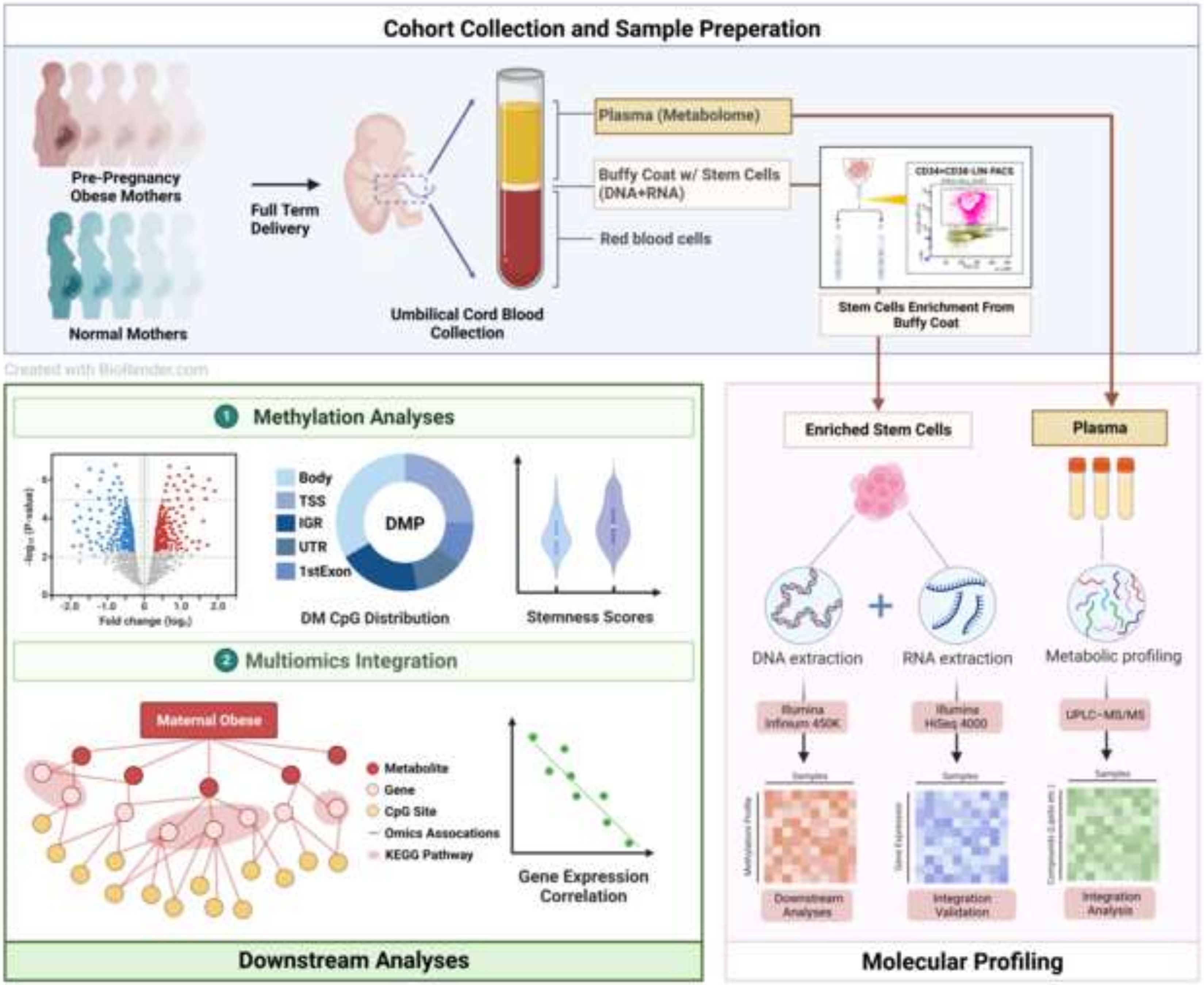

Figure 2

Click here to access/download;Figure;Figure 2.png

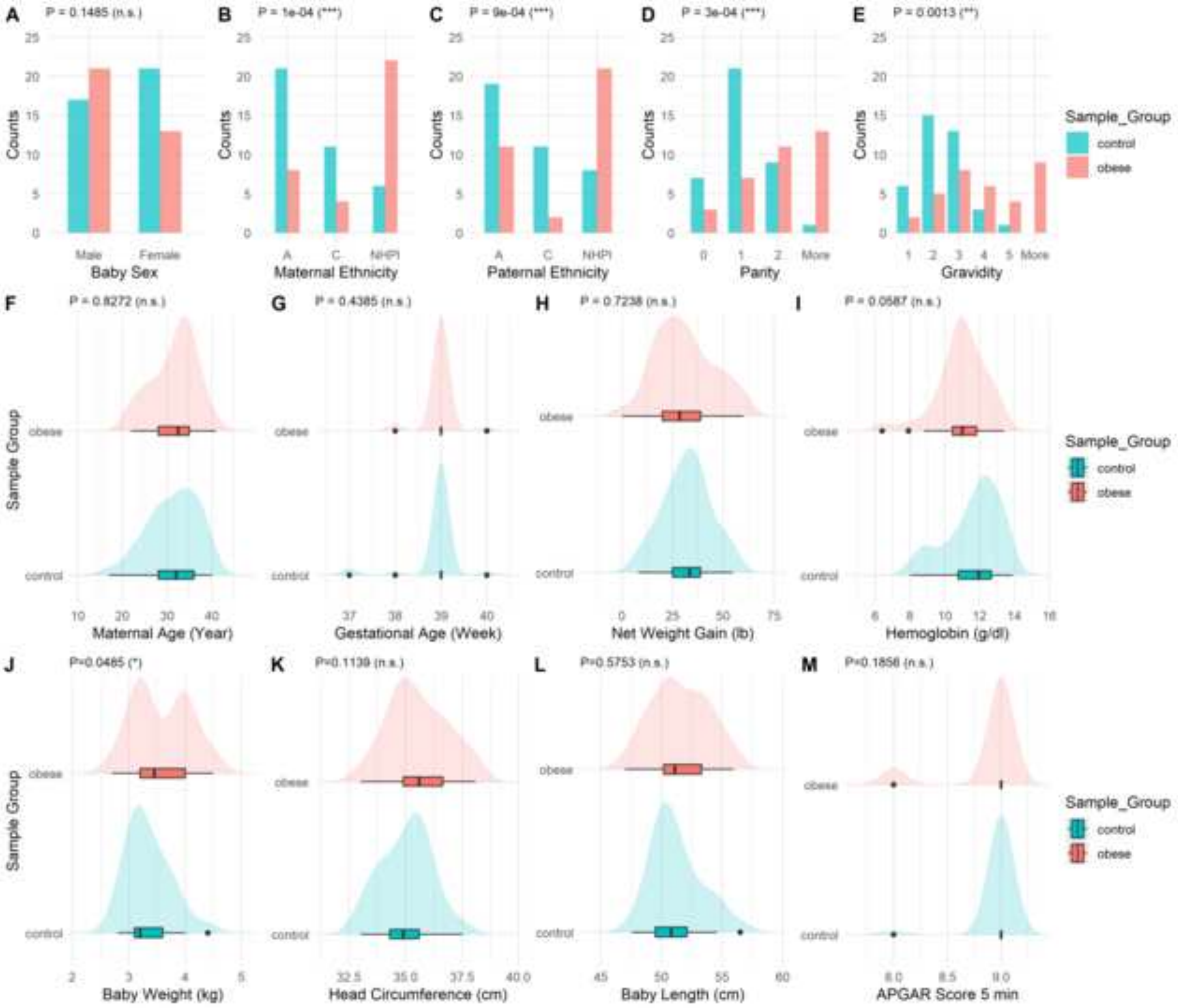

Figure 3

Click here to access/download;Figure;Figure 3.png

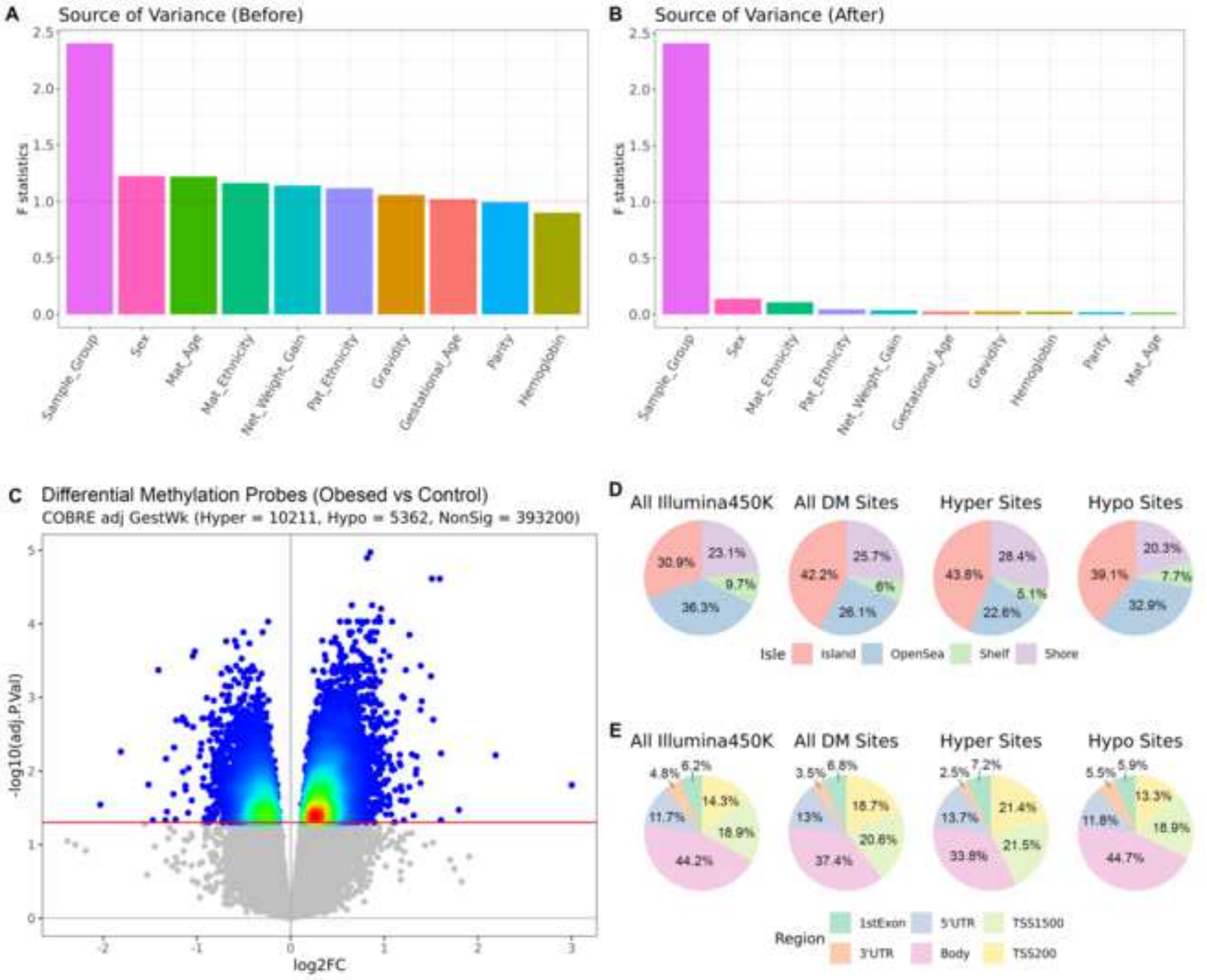

Figure 4

Click here to access/download;Figure;Figure 4.png

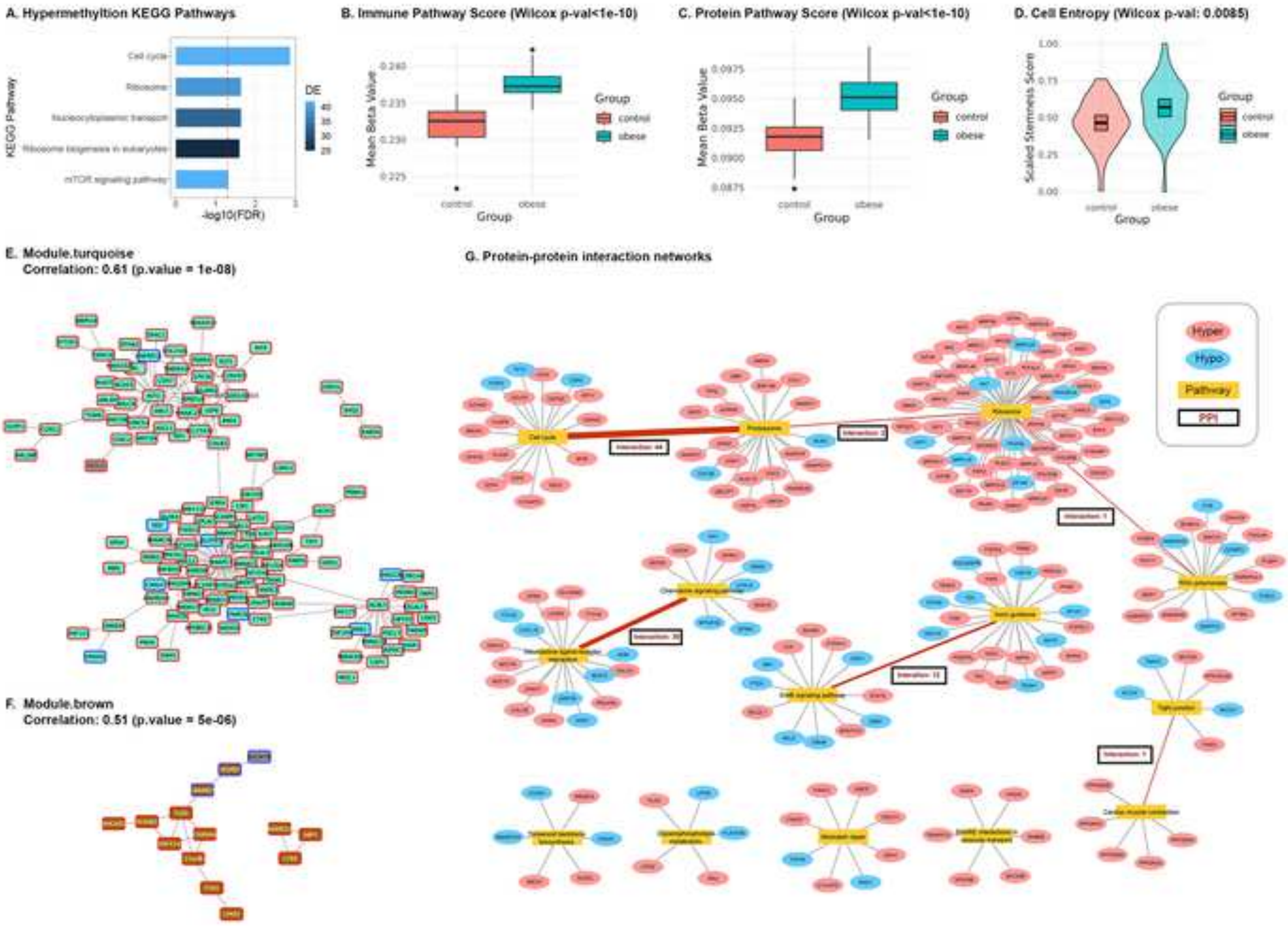

Figure 5

Click here to access/download;Figure;Figure 5.png

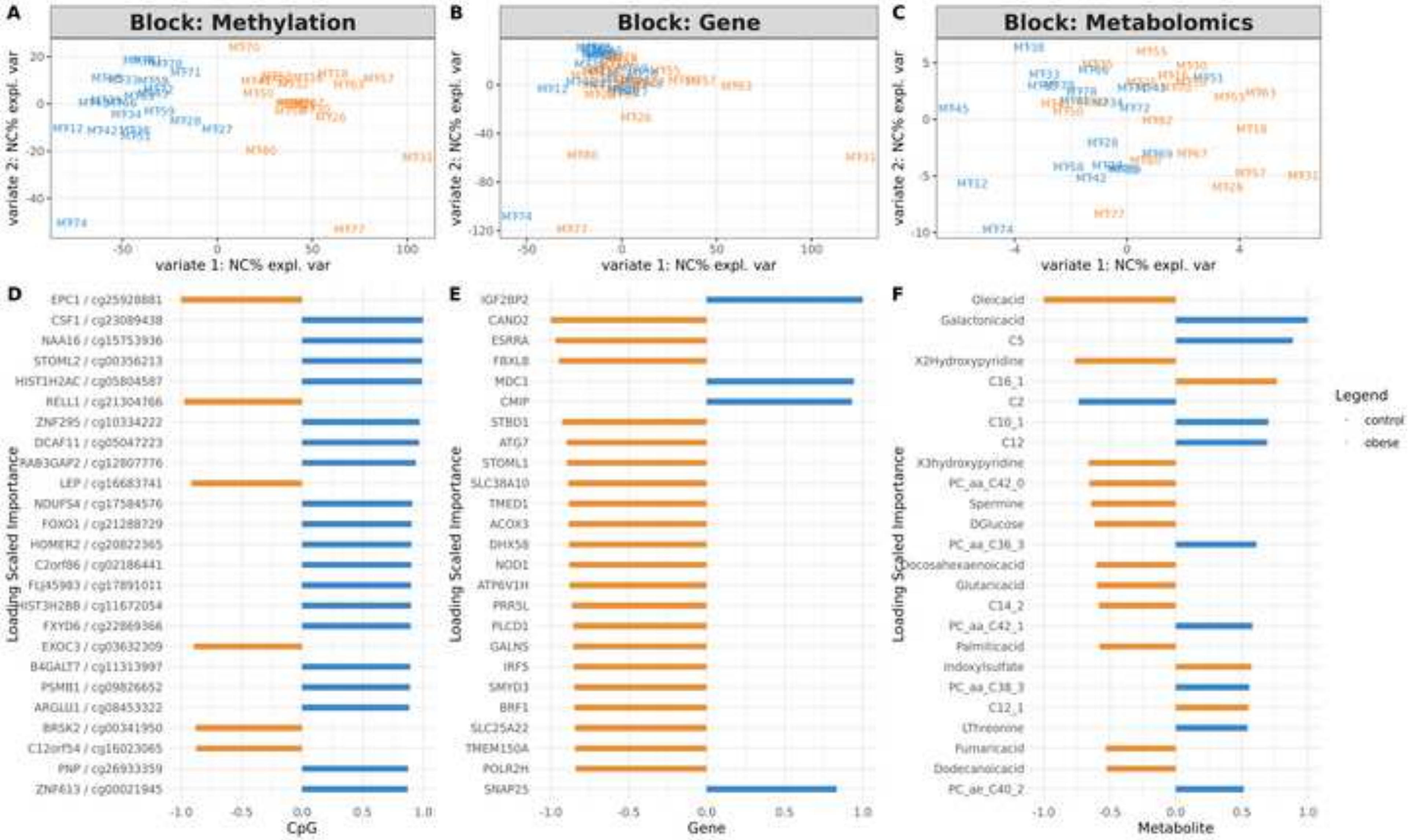

Figure 6

Click here to access/download;Figure;Figure 6.png

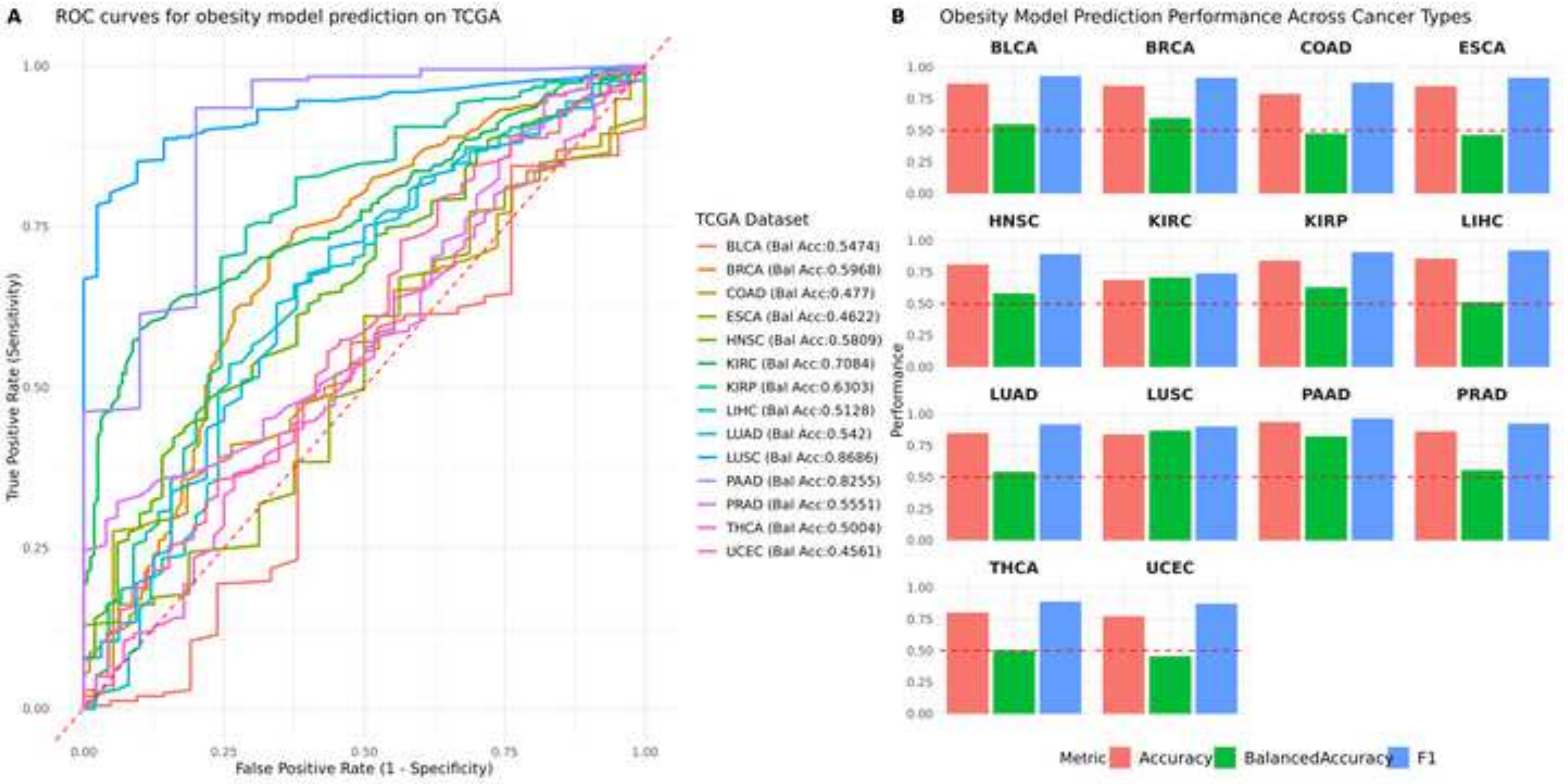

Figure 7

Click here to access/download;Figure;Figure 7.png

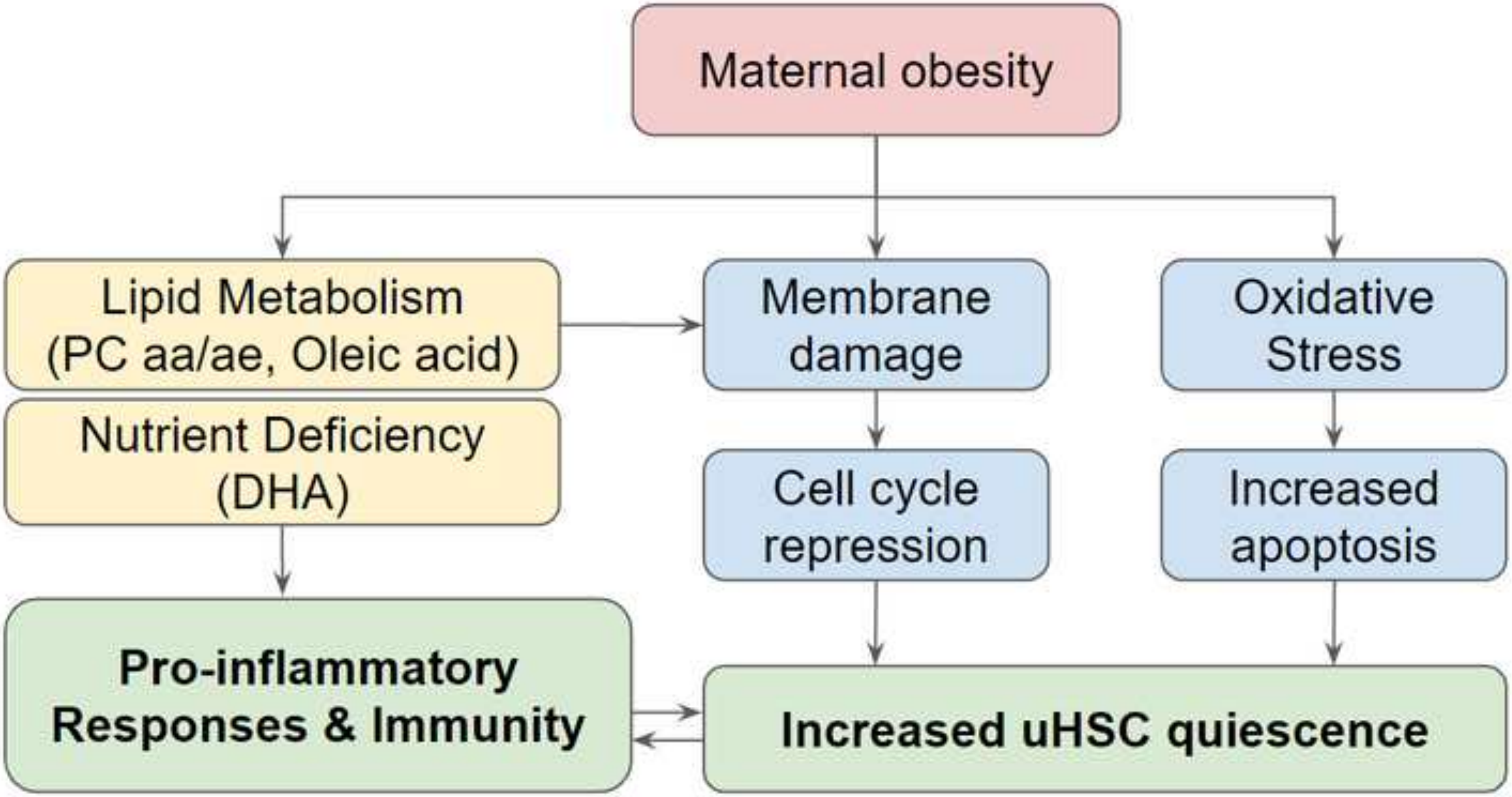

Supplemental figures and tables

Click here to access/download
Supplementary Material
Supplementary_Figures_Tables.docx

Cover letter
Click here to access/download;Personal Cover;CoverLetter_CordBloodMultiOmics_12_28_updated.docx

University of Michigan Medical School
Department of Computational Medicine & Bioinformatics

Dec 24th, 2024

Dear Dr Hans Zauner,

Thank you for passing on the reviewers reports on our initial manuscript GIGA-D-24-00395, which is now titled "Multi-omics Analysis of Umbilical Cord Hematopoietic Stem Cells from a Multi-ethnic Cohort of Hawaii Reveals the Intergenerational Effect of Maternal Pre-Pregnancy Obesity" at GigaScience. Please see attached manuscript revision (highlighted) along with the point-by-point answer to the reviewers.

We would like to explain the carefully thought study design, behind the limited sample size. First, we only recruited healthy patients who elected C-sections (rather than natural deliveries), to avoid confounding in laboring which would cause differences in gene expression and metabolomics profiles; Secondly, the controls all had similar ages and other clinical phenotypes, except pre-pregnant obesity. We intentially chose such stringent criteria, for the consideration of statistical rigor. Lastly, this study is a multi-omics study on pre-enriched cord blood stem cells, it is not a single-omic DNA methylatio study. Thus the cost is much higher than the DNA methylation study alone: FACS sorting for stem cell enrichment is $200/sample without accounting for the cost of antibodies; DNA methylation array is $300/sample; bulk RNA-Seq experiment is $250/sample; metabolomics assay is $200/sample; single-cell RNA-Seq was $5000/sample. The total cost was about $100,000 on the experiments.

We appreciate the comments from both reviewers, who really helped to improve the quality of the work. Major revisions include but are not limited to:

-additional QC steps with detailed description (mismatch, genomics inflation check, QQ plot, cross-hybridization CpG site removal etc)

-re-do the classification modeling using obesity samples, and re-do the prediction on TCGA samples

-comparison with previous DNA methylation results on maternal obesity

-address the limitation of this study (sample size etc) in the Discussion.

This is one of the rare multi-ethnic multi-omics studies that include polynesians and native Hawaiian, some of the nicest people in the world with ALOHA spirit. I owe them to report this study, which is about 10 years in the making. Thank you for your consideration. Please feel free to email me if you have any questions regarding this submission. lgarmire@umich.edu

University of Michigan Medical School, Department of Computational Medicine and Bioinformatics
2017 Palmer Commons, 100 Washtenaw Avenue, Ann Arbor MI 48109-2218
TELE: 734-615-5510 • FAX: 734-615-6553 •
www.ccmb.med.umich.edu

Lana Garmire, PhD
Associate Professor
Department of Computational Medicine and Bioinformatics
Medical School, University of Michigan, Ann Arbor, 48105, USA

Click here to access/download;Personal Cover;Gigascience_response_letter_to_reviewers_12_29_2024.d

Reviewer #1: Comments to the author: This novel multiomics study provides insights to biological pathways linking pre-pregnancy maternal obesity to offspring health, particularly cancer risk. Below, I have some comments, particularly related to potential confounders in an observational study such as this.

1. Title (and throughout the manuscript): "transgenerational" should be "intergenerational" since this study does not address germ line effects.

Thank you for the comment. We replaced “transgenerational” with “intergenerational.” as suggested.

2. Please add additional details on DNAm data QC. What was the source of batch effects removed? Did you drop cross-hybridizing probes? Were additional sample-level QC steps performed, including checking for sex mismatch and median intensity values?

Thanks for the suggestion. We added additional details on DNAm data QC in the Methods section “Methylation data pre-processing”. We also added a new **Supplemental Figure 1** showing our preprocessing workflow.

The source of batch effects includes 450K plate and array information. We removed the batch effect using ComBAT as described in the method section previously.

Thank you for pointing out additional QC steps, including sex mismatch, median intensity values and removal of cross-hybridizing probes. Below are the details:

Sex mismatch: No sex mismatch was found, shown by the contingency table. Sex was predicted using *minfi getSex* function, using median values of measurements on the X and Y chromosomes respectively.

		Prediction	
		Female	Male
**Obs**	Female	38	0
	Male	0	34

Median intensity values: Median intensity values of methylated probes and unmethylated probes are within the optimal range of Illumina 450K with intensities from 3000-7000. No significant outliers were found, neither the case nor control groups show clear separations, in the scatter plot (right).

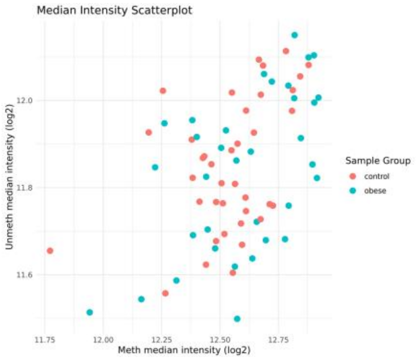

Drop cross-hybridizing probes: We obtain the cross-hybridization probes from DMRcate in ExperimentHub, with query id ‘EH3129’. After cross-checking, 1992 cross-hybridization probes were presented in the original beta matrix.

We redid the differential analysis (confounder adjusted) after removing cross-hybridization probes, using a new set of differential probes to redo all downstream analyses. In addition to the above suggested QC steps, we included additional QC results, such as the multi-dimensional scaling (MDS) plot, the distribution of methylation raw intensities, and the singular value decomposition (SVD) heatmap of methylation data with clinical confounders. They are in the new **Supplemental Figure 2.**

We updated **Figure 3** and **Figure 4** with the corrected result of differential probes removing cross-hybridizing probes. Since these probes are trivial for pathway and functional analysis, the main finding of this study did not change.

3. Did you adjust for confounders in the differential expression analysis?

Yes, the differential expression analysis was adjusted for the confounders based on the Source of Variance (SOV) result on gene expression data. The model includes the Sample_Group, Mat_Age, Sex, Hemoglobin, Sample_Group, Net_Weight_Gain, Mat_Ethnicity, Gravidity, Parity. The limma voom transformation and SOV plots are shown in the new **Supplemental Figure 3A-B**.

4. Do you have data on socioeconomic factors? Considering the factors that are unbalanced between the groups, this could also be a source of biological variation.

Unfortunately, we did not collect socioeconomic data from recruited patients. This is one of the weaknesses of this study, we address it in the discussion.

5. Please provide Q-Q plots and genomic inflation values for EWAS results. This will help to confirm if the confounder adjustment was successful.

The Q-Q plots before and after confounder adjustment are shown in **Supplemental Figure 4** and also attached below:

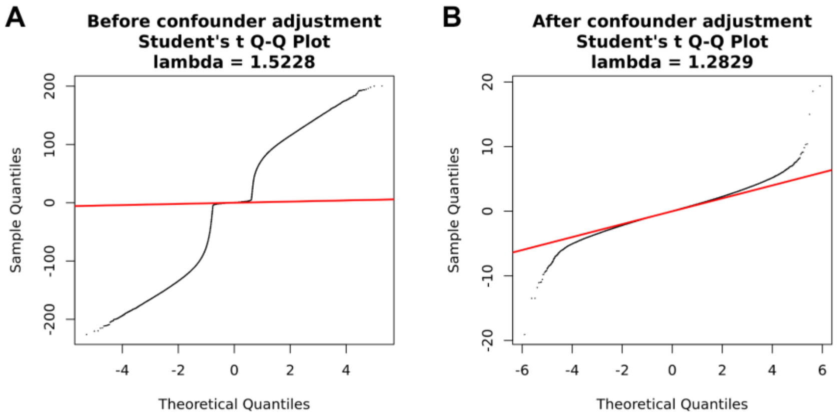

We used *bacon*, a Bayesian method for inflation control, to calculate genomic inflation values (λ) for our EWAS results. Before adjustment, λ was 1.52, and after confounder adjustment, it decreased to 1.28, demonstrating that adjusting for clinical confounders effectively reduced false positives in identifying differential CpG sites.

Since λ = 1.28 is moderately elevated, we performed sva analysis, but no surrogate variables were identified for correction. To ensure the observed inflation reflected true biological signal rather than systematic bias, we randomly shuffled sample group labels in the confounder-adjusted limma model. The resulting null λ was 0.96, confirming again there is no systematic bias.

We acknowledge that the observed genomic inflation reflects the complexity and variability inherent to EWAS in smaller cohorts and have outlined this as a limitation of our study. Future studies with larger sample sizes and replication datasets will be essential to validate these findings further.

6. Due to the unequal distribution of some factors between the groups, including ethnicity and parity, I am concerned about the effects of unmeasured confounders, particularly in analyses that did not include confounder adjustment. Can the authors address this limitation?

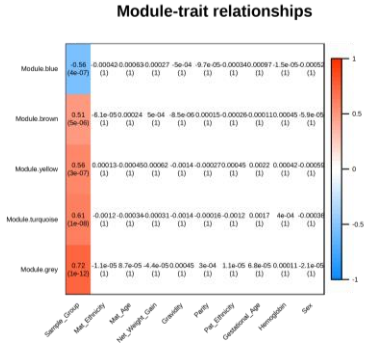

Thanks for the comment. All of our downstream analyses used confounder-adjusted CpGs as the input, therefore the confounding effects of ethnicity and parity were considered. For example, in WGCNA we specifically used adjusted beta values (residual of the confounder-only model). The WGCNA module-trait plot (**Supplemental Figure 6A**) which is attached below shows the success of confounder adjustment since all modules found are significantly related to the sample group only.

7. Do the differentially methylated CpGs overlap with CpGs previously associated with maternal pre-pregnancy obesity or BMI in the literature?

Thank you for the suggestion. We have added a paragraph in the Discussion comparing differentially methylated (DM) CpGs identified in our study with previously reported maternal pre-pregnancy obesity-associated CpGs. Quoted below:

"... Previously, the association between maternal obesity and epigenetic modifications has been investigated across various tissue types (e.g., adipose tissue, liver, cord blood) and species (e.g., human, mouse) [105]. We cross-checked our findings with these reports, many of which align providing further validation of their biological significance. For example, 33 CpGs across 20 genes, such as those in TAPBP (cg17621507, cg23922433, cg27385940), TNFAIP8

(cg18689486, cg07376834, cg03723497,cg21130861), and AGPAT1 (cg09043226, cg25733934, cg08049198, cg18191873) in our study are consistent with the cord blood leukocyte DNA methylation study from Martin et al with the same study objective [106]. TAPBP, TNFAIP8, and AGPAT1 play roles in immune function, transcriptional regulation, and lipid metabolism respectively. Additionally, our analysis also identified different CpG probes within the same genes previously associated with maternal obesity, offering additional insights into their epigenetic regulation. For instance, we observed different CpGs in HLA-E (cg01462744, cg02942965, cg26175526), ALPK1 (cg04779144, cg10855342), and PTEN (cg09472211). These genes were also reported from the Boston Birth Cohort study [107]. We identified different hypermethylation sites on MYT1L (cg05786278, cg17519749, cg21239227) and IGF1R (cg01284192, cg06596307, cg08138544, cg16918683, cg26577252), genes; these genes also showed high methylation levels in the cord blood (on different CpG sites) reported by Josefson et al [108]. Additionally, in our gene expression and methylation correlation analysis of uHSC, HOXA9 and HOXA5 emerged as the top genes (**Supplemental Figure 3D**), displaying strong correlations between expression and methylation levels. These hypomethylated genes (HOX family genes), along with 25 additional commonly identified genes, are consistent with the finding in the DNA methylation study on leukocytes of cord blood samples [109], linking them to maternal lipid and cholesterol levels. In our study, HDAC4 and PLEC1 stand out for their strong associations with obesity-related traits among the top differentially methylated CpG sites. Hypermethylation of cg05995464 in HDAC4 was previously reported to be associated with childhood obesity [110]. PLEC1 is a critical gene for extracellular matrix remodeling in adipose tissue, and hypomethylation of cg20784950 in PLEC1 is evident in our study. Lower PLEC1 methylation was previously correlated with higher BMI and obesity status [83,111]. Together, these comparisons underscore the robust and overlapping epigenetic patterns associated with maternal pre-pregnancy obesity."

8. Discussion: Can the authors address the strengths and limitations of this study? (e.g., target tissue vs. tissue studied, sample size, generalizability)

Thank you. We now include a new paragraph on the strengths and limitations of this study, quoted below:

There are some caveats of this study related to the study design. First, this is a single-site study with a relatively small sample size, and along with some genomic inflation the statistical power of the findings is limited. This is especially the case for the gene expression omic layer, where individual DE genes are lacking. This may have limited maternal obesity CpG biomarker identification, which resulted in positive risk associations in some, but not all of the 14 TCGA cancers, in the classification model (**Figure 6**). When the budget allows, a large-scale multi-site population study is desirable. Secondly, we use the stem cell population in the cord blood as the surrogate for "stemness" property investigation, to link the offspring's disease with maternal obesity. It is most feasible and practical to collect cord blood cells, and the painstaking measurements of the uHSC population avoid blood cell type heterogeneity issues, which may confound the cord blood DNA methylation result significantly [81]. However, this approach may very well be simplified and biased, as stem cells exist in many body parts of babies. Therefore, extrapolations from uHSC need to be cautioned. Further, our phenotypic data collection focused on the physiological and demographic information and missed socioeconomic data. Thirdly, environmental, lifestyle or social determinants may act as confounders and influence the observed outcomes, which are not measured nor adjustable in the study, per the protocol. Some of these measurements, such as lifestyle and health insurance, can be mitigated by incorporating electronic health record data, similar to what we have done [112,113]. Additionally, an

important aspect of maternal-offspring study is to longitudinally follow them up for health outcomes later in life. The IRB for this study was not designed for such an investigation, unfortunately. Nevertheless, this uHSC multi-omics study provides a critical initial lens peeking into the immune-metabolic mechanisms, which serves as the foundation for all the possible expansion work mentioned above.

9. Line 484: "ping-point" should be "pinpoint"

We have corrected the spelling in the revised manuscript.

10. Please define "NHPI" in the manuscript.

NHPI stands for Native Hawaiian and Pacific Islander. We now define it in the manuscript upon its first appearance.

Reviewer #2: Du et.al. investigated the association between maternal obesity and epigenetic markers in cord blood steam cells in a small pregnancy study. The manuscript has some weakness that are mostly related to the small sample size and the interpretation of the result considering this.

Abstract:

1. The decision to analyse the cell type CD34+/CD38-/Lin- is not well justified in the abstract.

We hypothesize that the intergenerational effect of maternal obesity is carried out by the epigenetic changes in the cord blood stem cell population. We modified the abstract to make it clearer.

2. The sentence in the results is not well justified: "Comprehensive functional analysis showed hypermethylation in promoters of genes involved in cell cycle, protein synthesis, immune signaling, and lipid metabolism"

We have clarified the sentence in the abstract. KEGG pathway enrichment, WGCNA, and PPI analyses revealed hypermethylated CpG sites were involved in critical biological processes, including cell cycle, protein synthesis, immune signaling, and lipid metabolism.

3. There is a strong bias in which CpGs are included in the Illumina arrays, which does include cell cycle, protein synthesis etc, so given a set of randomly picked CpGs, they would often tend to cluster in those processes.

We appreciate the reviewer's concern regarding biases in CpG inclusion on Illumina arrays. Recognizing the background CpG biases, we conducted the hypergeometric tests, which estimate pathway enrichment given the background biases. After FDR adjustment on the hypergeometric test result, the p-values for the identified pathways (e.g., cell cycle, protein synthesis, etc.) are still statistically significant. This confirms that the enrichment of significant CpGs in these pathways is unlikely to occur by chance and reflects meaningful biological

associations. We have added a new hypergeometric test-based pathway enrichment step in the revised Methods section titled “KEGG Pathway Enrichment Analysis”.

	Pathway	N_gene	N_CpG	N_TSS_CpG	DE	FDR	sig_cpg	hyper_geo_p_value	hypergeo_adjusted_p_value
	<chr>	<dbl>	<dbl>	<dbl>	<int>	<dbl>	<int>	<dbl>	<dbl>
**path:hsa03008**	Ribosome biogenesis in eukaryotes	109	1001	421	25	0.023608726	38	3.497122e-26	3.121506e-25
**path:hsa03010**	Ribosome	158	1620	827	39	0.021515098	46	1.668410e-22	1.116908e-21
**path:hsa03013**	Nucleocytoplasmic transport	108	1498	640	33	0.021515098	47	4.615489e-28	4.836230e-27
**path:hsa04110**	Cell cycle	126	3105	985	42	0.001224057	63	1.232111e-33	3.299319e-32
**path:hsa04150**	mTOR signaling pathway	156	4172	1049	42	0.044716016	89	5.659943e-57	6.820231e-55

4. The notion that the CpGs associated with maternal obesity would highly predict between cancer and controls (in cross sectional samples) is a bit absurd, since almost any set of randomly picked CpGs would predict very well between those two groups.

Thank you for the comment. The previous test using CpGs associated with maternal obesity to predict TCGA cancers was loosely done: we did not build the obesity model using the CpGs associated with maternal obesity first, rather, we only used these CpGs from the key pathways as features to build classification models in TCGA. As a result, it is potentially misleading as the reviewer pointed out. Now we changed the model to be a strictly obesity-based classification model using the CpGs in these key pathways and then applied this model (with the same features and feature importance weights) on the TCGA pan-cancers data to classify the tumor and adjacent normal. Three cancer datasets showed high balanced accuracy for prediction: LUSC (0.8686), PAAD (0.8255), KIRC (0.7084). The details of model building, result, and limitation are addressed in the Method, Result and Discussion sections.

Methods

5. The word transgenerational is not well justified in the analysis, since they are mainly looking at an exposure (Maternal BMI) on the biomarker in the newborn. For it to be truly transgenerational, it has to go through generations (possibility even grandchildren).

Thank you for the comment on the word of choice. We will replace the word “transgenerational” with “intergenerational”.

6. The genomic inflation factor is not calculated, which would give an impression of the study power, or lack thereof.

The Q-Q plots before and after confounder adjustment are shown in **Supplementary Figure 4.**

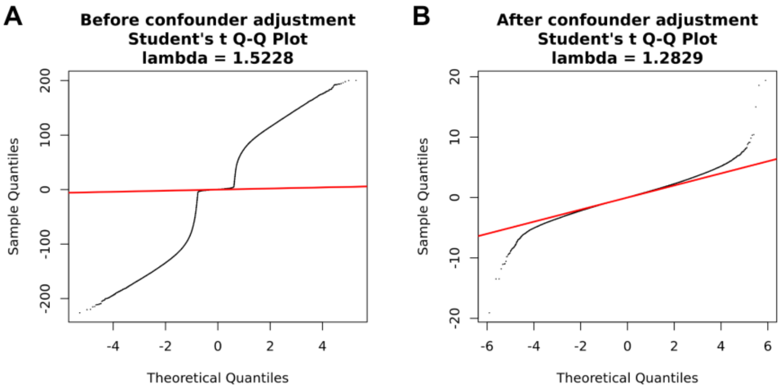

We used *bacon*, a Bayesian method for inflation control, to calculate genomic inflation values (λ) for our EWAS results. Before adjustment, λ was 1.52, and after confounder adjustment, it reduced to 1.28, indicating that adjusting for clinical confounders effectively reduced false positives in identifying differential CpG sites.

Given that λ = 1.28 is moderately elevated, we performed sva analysis, but no surrogate variables were identified for correction. To ensure the observed inflation reflected the true biological signal rather than systematic bias, we randomly shuffled sample group labels in the confounder-adjusted limma model. The resulting null λ was 0.96, confirming the no systematic bias in our results.

We recognize that the observed genomic inflation may partly reflect the inherent challenges of working with smaller cohorts in EWAS studies, and weakness is addressed in the discussion.

Discussion

7. The very small sample size should be mentioned as a potential weakness of the study.

Good point. This is added to the limitation part of the Discussion section. We also emphasize the need for large multi-site, independent replication cohorts in future studies to further confirm our findings.

While recognizing the sample size is modest, we would like to explain to this reviewer about the study design that led to this modest sample size. First, we only recruited healthy patients who elected C-sections (rather than natural deliveries), to avoid confounding in laboring which would cause differences in gene expression and metabolomics profiles; Secondly, the controls all had similar ages and other clinical phenotypes, except pre-pregnant obesity. We intentionally chose such stringent criteria, for the consideration of statistical rigor. Lastly, this study is a multi-omics study on pre-enriched cord blood stem cells, it is not a single-omic DNA methylation study. Thus, the cost is much higher than the DNA methylation study alone: FACS sorting for stem cell enrichment is $200/sample without accounting for the cost of antibodies; DNA methylation array is $300/sample; bulk RNA-Seq experiment is $250/sample; metabolomics assay is $200/sample; single-cell RNA-Seq was $5000/sample. The total cost was about $100,000 on the experiments alone.
